# The proteome of the calcified layer organic matrix of turkey (*Meleagris gallopavo*) eggshell

**DOI:** 10.1186/1477-5956-11-40

**Published:** 2013-08-27

**Authors:** Karlheinz Mann, Matthias Mann

**Affiliations:** 1 Max-Planck-Institut für Biochemie, Abteilung Proteomics und Signaltransduktion, D-82152, Martinsried, Am Klopferspitz 18, Germany

## Abstract

**Background:**

Chicken eggshell mineralization is a prominent model for biomineralization not only because of its importance for avian reproduction but also because of the commercial interest associated with eggshell quality. An analysis and comparison of the protein constituents of eggshells of several species would contribute to a better understanding of the shell mineralization process. The recent publication of the turkey genome sequence now provides a basis for the in-depth analysis of the turkey eggshell proteome.

**Results:**

Proteomic analysis of turkey acid-soluble and acid-insoluble organic eggshell matrix yielded 697 identified proteins/protein groups. However, intensity-based absolute quantification (iBAQ) results indicated that the 47 most abundant identified proteins already constituted 95% of the total turkey eggshell matrix proteome. Forty-four of these proteins were also identified in chicken eggshell matrix previously. Despite these similarities there were important and unexpected differences. While ovocleidin-116 and ovocalyxin-36 were major proteins constituting approximately 37% of the identified proteome, other members of the group of so-called eggshell-specific proteins were not identified. Thus ovocalyxin-21 and ovocalyxin-32 were missing among matrix proteins. Conversely, major turkey eggshell proteins were not detected in chicken, such as the bone protein periostin, the mammalian counterpart of which is involved in many aspects of bone metabolism and which represented 10-11% of the total identified proteome.

**Conclusions:**

Even members of the same avian family show important differences in eggshell matrix composition and more studies on the proteome and the transcriptome level will be necessary to identify a common toolkit of eggshell mineralization and to work out species differences among functional eggshell protein sets and their role in eggshell production.

## Background

The avian eggshell is a bioceramic formed of calcium carbonate and an organic matrix pervading and enveloping the calcite crystals. Eggshell formation is the last step of egg production, a process most comprehensively studied in the chicken due to its commercial importance [1,2]. Egg production starts in the ovary by massive yolk accumulation for six to ten days. Most yolk components are produced in the liver, secreted into the blood stream and transported to the ovary where they are taken up by receptor-mediated transport at the oolemma, the plasma membrane of the egg cell [3]. After ovulation the egg, the bulk of which is yolk covered by a proteinaceous inner vitelline membrane, enters the oviduct to start an approximately 22 h-long journey driven by peristaltic movements of the oviduct wall. In the first section of the oviduct, the infundibulum, the egg is covered by the outer vitelline membrane. Egg white (albumen) production takes place in the next section of the oviduct, the magnum. Eggshell formation starts in the white isthmus by assembly of the eggshell membranes from soluble components secreted by cells lining the oviduct. First small calcite accretions form on specialized, regularly spaced nucleation sites on the external shell in the red isthmus [4]. Bulk mineralization takes place in the eggshell gland (uterus), where the egg stays for 16-17 h. The final step in eggshell assembly is the deposition of the cuticle, which also covers the openings of eggshell pores traversing the calcified layer. Eggshell calcification is thought to be controlled by matrix proteins secreted by epithelial cells lining isthmus and uterus [5-7]. However, our previous proteomic analyses of the soluble proteome of the chicken calcified eggshell layer [8,9] indicated that the matrix does not only contain proteins produced by the shell gland epithelium but also proteins produced and secreted in other sections of the oviduct. Apparently leftovers of the assembly processes along the egg production line migrate with the egg to end up in the uterus fluid where they are eventually incorporated into the growing calcitic layer. Several such proteins, especially the major egg white proteins ovalbumin [10], lysozyme [11] and ovotransferrin [12] were shown by immunohistochemical methods to reside in the intra-crystalline matrix itself and not to be just surface contaminants. This location was also confirmed for osteopontin, a protein previously identified in bone, but also secreted in the eggshell gland triggered by the entry of the egg and is therefore an example of a protein occurring in different biomineral systems of one organism [13]. However, although egg white proteins may weakly influence calcium carbonate crystallization *in vitro*[11,12,14,15], their role, if any, in eggshell mineralization remains unclear. In addition to egg proteins the matrix proteome contained basement membrane components, endoplasmatic reticulum residents, Golgi complex proteins, and other intracellular compartments. These may have reached the oviduct fluid as by-products of secretion or may have been released by damaged cells of the oviduct epithelium. In total, the soluble proteome of the mineralized layer, the thickest eggshell compartment, comprised more than 520 proteins [8,9]. Further proteomic studies of insoluble proteins of the calcified layer and the cuticle [16] and solubilized cuticle [17] contributed several interesting new proteins to the overall chicken eggshell proteome. This unexpected complexity of the eggshell proteome raised the problem of how to discern between matrix proteins functioning in matrix assembly and calcification and a background of non-functional proteins. Because birds are not as easily accessible to genetic manipulation as unicellular or some invertebrate species, one has to resort to more practicable methods.

The concept that functional eggshell proteins should be produced in the eggshell gland has stimulated gene expression studies with microarrays comparing differential expression between eggshell gland tissue of juvenile and sexually mature hens [18] and between eggshell gland tissue and tissues of other oviduct sections [19]. Of these studies, the second one may be more relevant to the identification of eggshell proteins, because it focused on the eggshell gland during deposition of the eggshell. Altogether 605 transcripts were highly expressed in uterine tissue as compared to other oviduct sections and 437 corresponded to known proteins present in protein sequence databases. Of these, 52 corresponded to matrix constituents identified by proteomics or protein biochemical methods previously. However, many of these transcripts or corresponding proteins are not likely to play a role in eggshell production, because the list also contains such proteins as tubulin, actin, glyceraldehyde-3-P-dehydrogenase or ezrin. A disadvantage of such transcriptomic studies is that they will not recognize major eggshell proteins such as ovotransferrin, ovalbumin or serum albumin, which are not produced in the eggshell gland but may nevertheless influence eggshell mineralization and eggshell properties in some way [11,12,14,15]. Another possible way to identify functionally important eggshell proteins, and the one we pursue here, is the comparison of eggshell matrices of different avian species.

Mass spectrometry (MS)-based in-depth and high-throughput studies depend on the availability of comprehensive sequence databases created by genome sequencing projects. The first available avian genome sequence was that of chicken [20] followed by zebra finch [21] and turkey [22] genome sequences recently. For the present study we chose the turkey eggshell because turkey belongs to the same family as chicken (Phasianidae) and has a very similar genome [22,23]. The present proteomic analysis of the turkey calcified eggshell layer acid-soluble and acid-insoluble proteins showed many similarities, but also unexpected differences, between chicken and turkey eggshell proteomes.

## Materials and methods

### Preparation of proteins and peptides

Turkey eggshells (strains Converter and Big 6) were obtained from Putenzucht Miko GmbH, A-4871 Zipf, Austria. These were fertilized, but not incubated eggs. The empty shells of five eggs of each strain were washed under tap water and then cleaned in 5% EDTA for 60min at room temperature to facilitate mechanical removal of membranes, cuticle and attached contaminants. The membranes were peeled off and the cuticles were removed by rubbing under flowing de-ionized water. The pieces of calcified eggshell were washed with water, dried and demineralized in 10% acetic acid (1 g of shell/20 ml) overnight in the cold room. The suspension was centrifuged for 1 h at 4°C and 12000g_av_ to separate acid-soluble from acid-insoluble matrix. The pellets were washed three times by re-suspension in 10vol 10% acetic acid, centrifugation at 4°C and 12000g_av_ for 30min, and lyophilized. Supernatants were successively dialyzed against 3 × 10vol 10% acetic acid and 2 × 10vol 5% acetic acid at 4-6°C (Spectra/Por 6 dialysis membrane, molecular weight cut-off 2000; Spectrum Europe, Breda, The Netherlands) and lyophilized.

Proteins were separated by SDS-PAGE using pre-cast 4-12% Novex Bis-Tris gels in MES buffer, using reagents and protocols supplied by the manufacturer (Invitrogen, Carlsbad, CA), except that 1% β-mercaptoethanol was used as reducing agent in the sample buffer. The sample was suspended in 20 μl sample buffer/100 μg of matrix, heated to 70°C for 10 min, and centrifuged to remove sample buffer-insoluble material. Three lanes were loaded with 80 μg of organic matrix in each of three separate experiments per strain and fraction. Gels were stained with colloidal Coomassie (Invitrogen) after electrophoresis, and cut into suitable slices for in-gel reduction, carbamidomethylation and digestion with trypsin as described [24]. The molecular weight marker was Novex Sharp pre-stained (Invitrogen). The eluted peptides were cleaned with C18 Stage Tips [25] before mass spectrometric analysis.

### LC-MS and data analysis

Peptide mixtures were analyzed by on-line nanoflow liquid chromatography using the EASY-nLC 1000 system (Proxeon Biosystems, Odense, Denmark, now part of Thermo Fisher Scientific) with 20 cm capillary columns of an internal diameter of 75 μm filled with 1.8 μm Reprosil-Pur C18-AQ resin (Dr. Maisch GmbH, Ammerbuch-Entringen, Germany). Peptides were eluted with a linear gradient from 5-30% buffer B (80% acetonitrile in 0.1% formic acid) for 100 min, 30-60% B for 12 min and 80-95% B for 8 min at a flow rate of 250 nl/min. The eluate was electro-sprayed into an Orbitrap Elite (Thermo Fisher Scientific, Bremen, Germany) using a Proxeon nanoelectrospray ion source. The Orbitrap Elite was operated in a HCD top 10 mode essentially as described [26,27]. The resolution was 120,000 for full scans and 15,000 for fragments (both specified at m/z 400). Ion target values were 1e6 and 5e4ms, respectively. Exclusion time was 90 sec. Raw files were processed using the Andromeda search engine-based version 1.3.9.3 of MaxQuant (http://www.maxquant.org/) with enabled second peptide, iBAQ and match between runs (match time window 0.5min; alignment time window 20 min) options [28-30]. For protein identification the ENSEMBL turkey protein database from release 66, 2012, (http://www.ensembl.org/info/data/ftp/index.html) was downloaded and combined with the reversed sequences and sequences of common contaminants, such as human keratins. Carbamidomethylation was set as fixed modification. Variable modifications were oxidation (M), N-acetyl (protein) and pyro-Glu/Gln (N-term). The initial mass tolerance for full scans was 7 ppm and 20 ppm for MS/MS. Two missed cleavages were allowed and the minimal length required for a peptide was seven amino acids. The peptide and protein false discovery rates (FDR) were set to 0.01. The maximal posterior error probability (PEP), which is the individual probability of each peptide to be a false hit considering identification score and peptide length, was set to 0.01. Two sequence-unique peptides (sum of sequence-unique peptides in acid-soluble and acid-insoluble fractions of six technical replicates forming one biological replicate) occurring at least three times in total in two different technical replicates were required for high-confidence protein identifications. Identifications with only two sequence-unique peptides were routinely validated using the MaxQuant Expert System software [31] considering the assignment of major peaks, occurrence of uninterrupted y- or b-ion series of at least four consecutive amino acids, preferred cleavages N-terminal to proline bonds, the possible presence of a2/b2 ion pairs and immonium ions, and mass accuracy. The iBAQ (intensity-based absolute quantification) [32] option of MaxQuant was used to calculate, based on the sum of peak intensities, the approximate share of each protein in the total proteome.

Sequence database searches were performed with FASTA (http://www.ebi.ac.uk/Tools/sss/fasta/) [33] against current releases of Uniprot Knowledgebase (UniProtKB) and International Protein Index (IPI). Other bioinformatics tools used were clustalW2 for sequence alignments (http://www.ebi.ac.uk/Tools/msa/clustalw2/), InterProScan (http://www.ebi.ac.uk/Tools/pfa/iprscan/) [34] for domain predictions, SignalP 4.1 (http://www.cbs.dtu.dk/services/SignalP/) [35] for signal sequence prediction, and the Venn diagram plotter (http://omics.pnl.gov/software/VennDiagramPlotter.php) for preparing Venn diagrams.

## Results and discussion

**Figure 1:**
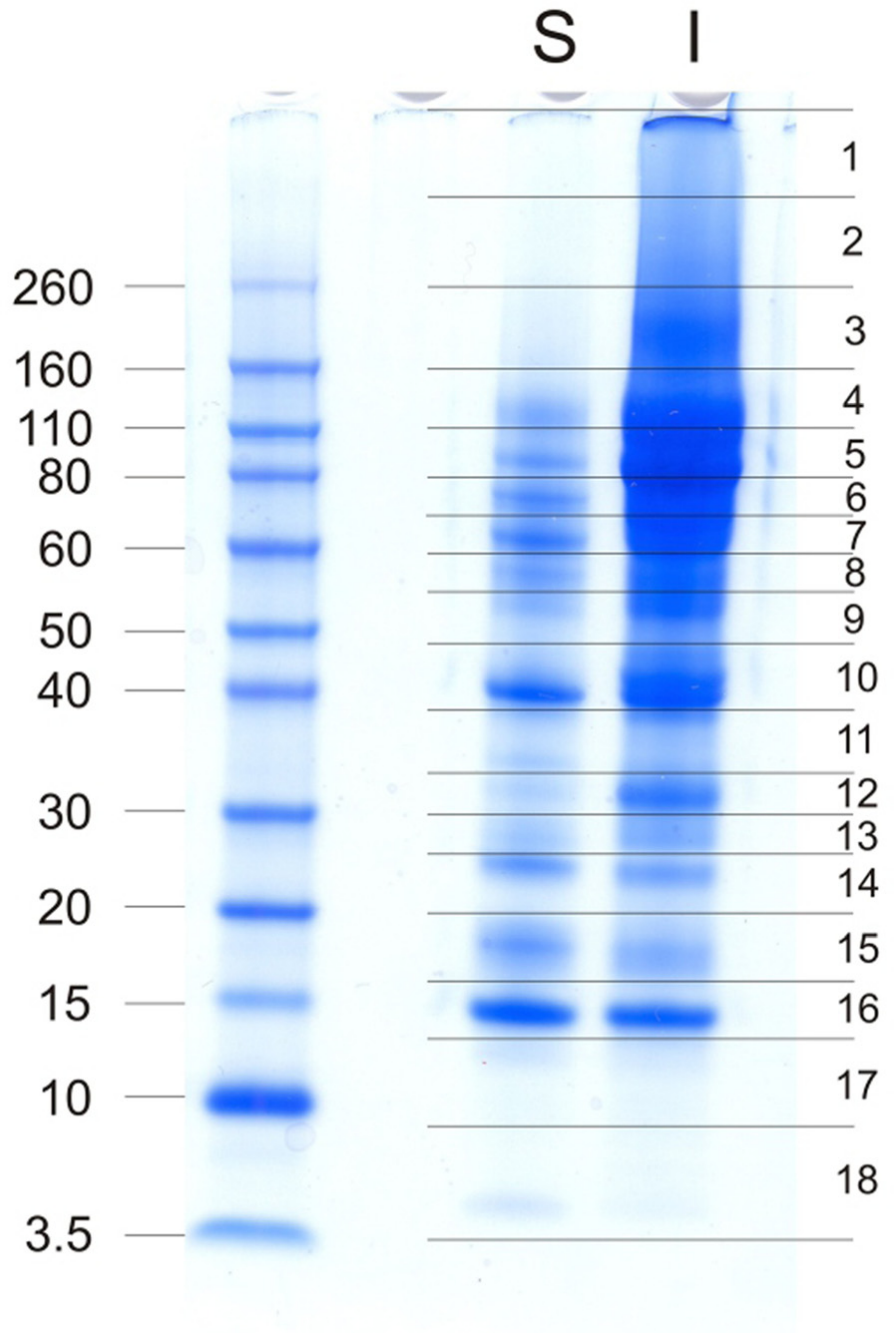
**SDS-PAGE of turkey eggshell matrix proteins.** Each lane was loaded with 80μg of matrix. S, acid-soluble matrix; I, acid-insoluble matrix. Molecular markers are shown to the left. Slices for in-gel digestion are indicated to the right.

**Figure 2:**
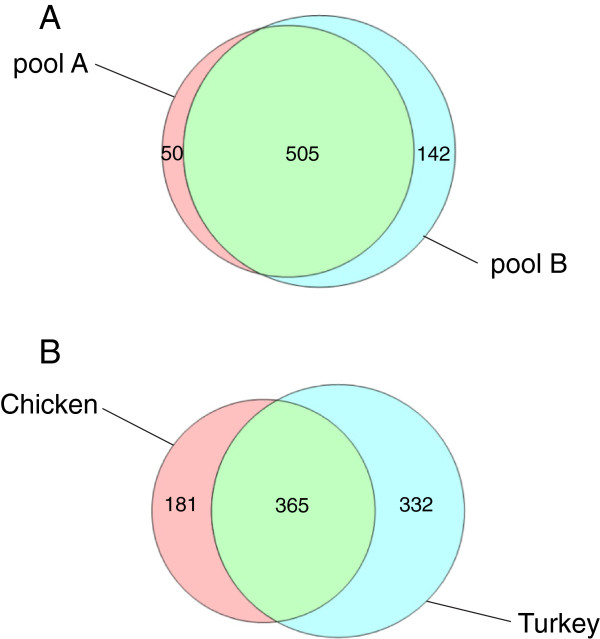
**Comparison of turkey biological replicate proteomes (A) chicken and turkey eggshell proteomes (B).** The number of identified chicken eggshell proteins were compiled from [8,9,16,17] and cover acid-soluble proteins of the calcified layer [8,9], insoluble proteins of the calcified shell [16], and cuticle proteins [17].

For this study we used two biological replicates each consisting of the pooled washed shell calcified layers of five eggs of either strain Converter (biological replicate A) or strain Big6 (biological replicate B). Each of these biological replicates was analyzed in six technical replicates, three for the acid-soluble fraction and three for the acid-insoluble fraction. Matrix yields were approximately 8mg of acid-soluble matrix and 16mg of acid-insoluble matrix per g of dry shell calcified layer, together constituting approximately 2.5% of the total shell weight. No differences were detected in PAGE patterns of samples from different strains (not shown). Acid-soluble eggshell matrix and acid-insoluble matrix were analyzed separately. For each technical replicate three identical PAGE lanes were cut into 18 slices for in-gel digestion (Figure 1). MS analysis of the eluted peptides produced a total of 216 raw files. Technical replicate groups were analyzed with MaxQuant, first separately, then after combining results of acid-soluble and acid-insoluble fractions of each strain. After grouping obvious fragments of identical proteins and almost identical proteins together, pool A (strain Converter) yielded 555 protein groups and pool B (strain Big 6) 647 protein groups, with an overlap of 505 protein groups (Figure 2A). Almost all of the protein groups identified in only one pool were of low or very low abundance, each protein representing between <0.0001 to <0.01% of the total proteome as judged by their iBAQ values. Furthermore, most of these proteins were also identified in the respective other pool but below statistical acceptance thresholds. Therefore these differences likely did not represent differences between strains, but rather experimental variation. The combined proteomes yielded 697 protein groups (Additional file 1: Table S1) that may represent a slightly lower number of proteins because not all fragments of identical proteins distributed over different database entries may have been identified unequivocally. This was more than the sum of different proteins identified in the acid-soluble shell proteome [8,9], the insoluble shell proteome [16] and the cuticle [17] of the chicken eggshell (Figure 2B), a finding that may also reflect technical progress. The overlap between total chicken and turkey eggshell proteomes was 52% and increased to 85% if only turkey proteins of >0.01% of the total proteome (172 proteins) were considered, and to 94% with proteins of greater than 0.1% abundance (47 proteins). This indicates that much of the differences occurred among the low and very low-abundance proteins. The numerical difference between combined chicken eggshell proteins and turkey eggshell proteins was 151 proteins (protein groups). This increase may be attributable to a large part to developments in mass spectrometry instrumentation (FT-ICR [8] versus the much faster Orbitrap Elite, this study). However, the greater number of technical replicates and the analysis of both, acid-soluble and acid-insoluble fraction, may also have contributed. The complete lists of identified protein groups including those not accepted for Additional file 1: Table S1 are contained in Additional file 2: Table S2 ProteinGroups pool A, and Additional file 3: Table S3 ProteinGroups pool B. The identified peptides are listed in Additional file 4: Table S4 Peptides pool A and Additional file 5: Table S5 Peptides pool B. These files also contain additional accession numbers for groups with more than one protein, numbers of total, razor and sequence-unique peptides, their distribution over gel sections, iBAQ intensities, peptide sequences, and other relevant data not only for accepted identifications but also for identifications with only one peptide or two peptides in only one technical replicate.

Of the 697 protein groups 122 were identified only in acid-soluble fractions and 20 only in acid-insoluble fractions (Additional file 1: Table S1). Most of these were identified in shells of only one turkey strain at low abundance (<0.001%) and may not be due to true solubility preferences but experimental variation. However, 38 proteins were identified in one fraction of both biological replicates and may therefore reflect real differences in distribution among solubility fractions (Additional file 1: Table S1). This may especially be true for proteins identified with more than three sequence-unique peptides or an abundance of >0.001%, such as the antimicrobial peptide NK-lysine (H9H1A3_MELGA), the possibly extracellular ribonuclease G1NAU2_MELGA, the protein similar to Cys-rich secretory protein 3 contained in entry G1NN67_MELGA, or galectin-3 (G1NLL4_MELGA).

Similar to the chicken eggshell proteome, the turkey eggshell proteome contained many proteins occurring in other egg compartments, especially the egg white. These are likely leftovers of egg assembly that migrate together with the egg from the site of their secretion into the oviduct lumen to the eggshell gland [8]. Other proteins may have reached the oviduct fluid as by-products of secretion, shedding of extracellular domains of membrane proteins, or may have been released by damaged epithelial cells. This mixture of proteins is then supplemented with proteins specifically secreted from uterus epithelia and may be inserted into the growing eggshell during mineralization.

When looking for functional eggshell proteins, an obvious choice is to inspect the major proteins, although minor proteins can of course also affect matrix assembly and mineralization, especially if they have catalytic properties. In previous studies we discerned major proteins from minor ones using the exponentially modified protein abundance index (emPAI), a quantification method relying on spectral counts and relating the number of identified unique parent ions to the number of theoretically possible peptides [36]. Although in principle well suited for the purpose of identifying major proteins, the results are not particularly intuitive and the division into abundance groups as practiced before is somewhat arbitrary [8]. In the present study we therefore used the more refined iBAQ procedure [32] as implemented in recent MaxQuant versions. This method is based on peak intensities of identified peptides and these can be normalized to the sum of all intensities yielding the percentage of each component in relation to the total proteome. This showed that 47 major proteins, or protein groups, with an individual percentage of >0.1 constituted approximately 95% of the total identified turkey eggshell proteome. Of these 47 proteins we previously identified 44 (94%) in the chicken eggshell proteome and 24 were among the 50 most abundant of the chicken matrix proteins (Table 1). Furthermore, the messages of 11 of them were previously found to be up-regulated in epithelia of egg-containing uterus in different transcriptomic studies (Table 1). However, there were also some unexpected differences as detailed below.

**Table 1:** The most abundant proteins of turkey eggshell matrix

**ENSMGAP and UniProtKB**	**Probable chicken homolog**	**Sequence**	**FASTA**	**Protein**	**% of total**^**1**^
		**identity**	**E-value**		
**Accession**					
00000007641	IPI00581368	79.8%	1.9^-135^	Ovocleidin-116 **+	31.30
(G1N6E1)	OC116				
00000004038	IPI00583974	90.9%	2.6^-150^	Ovalbumin **	12.52
(G1MYK6)	OVAL				
0000015961	IPI00595980	99.2%	0	Periostin	10.89
(G1NQ91)	F1P4N9				
00000007759	IPI00573506	78.8%	8.8^-151^	Ovocalyxin-36 **+	6.00
(G1N6M8)	Q53HW8				
00000000142	IPI00600069	87.6%	1.3^-68^	Extracellular fatty acid-binding protein (EXFAB) **	3.82
(G1MQ16)	EXFAB				
0000018878	IPI00588727	77.9%	3.7^-60^	EDIL3 **, fragment	3.70
(G3UT43)	F1NCN3				
00000018172	IPI00685019	83.2%	5.2^-72^	Ig light chains with overlapping peptide sets ** ^**2**^	2.81
(G3UR71)	F1NSD0				
etc.	etc.				
00000001491	IPI00601265	86.8%	5.0^-57^	Avidin *	2.67
(G1MSZ9)	AVID				
00000010561	IPI00574195	88.4%	0	Serum albumin **	2.57
(G1NCR2)	LBU				
00000002725	IPI00683271	87.3%	2.2^-203^	Ovotransferrin **	2.22
(G1MVN4)	TRFE				
00000007002	IPI00576782	90.6%	2.2^-56^	Cystatin **	1.67
(G1N522)	CYT				
0000019196	IPI00578559	76.9%	4.0^-138^	Polymeric Ig receptor **, fragment	1.59
(G3UTW1)	F1NE61				
00000008902	IPI00575013	95.7%	2.2^-191^	Similar to milk fat globule EGF factor 8 protein **	1.25
(G1N944)	E1C0K5				
0000015436	IPI00583503	97.6%	2.2^-58^	Similar to trefoil family peptide 2 (TFF-2)	1.13
(G1NP11)	E1BZ37				
00000002528	IPI00571581	93.1%	2.7^-173^	Sulfhydryl oxidase 1 **	1.08
(G1MV84)	QSOX1				
00000005746	Q90WR3	87.1%	1.2^-76^	Hemopexin, fragment **	0.80
(H9H0W2)					
00000005136	IPI00680258	89.3%	2.1^-150^	Similar to α2-antiplasmin/serpinF2 *+	0.80
(G1N0Z7)	F1NAR5				
00000005175	IPI00587267	97.9%	5.7^-173^	Similar to serpinF1/PEDF/α2-antiplasmin **	0.64
(G1N131)	E1C7H6				
00000010985	IPI00600859	95.2%	8.9^-70^	Lysozyme C **	0.64
(LYSC)	B8YK79				
00000017273	IPI00599414	95.9%	1.4^-100^	SPARC/BM-40/osteonectin *	0.57
(G3UNZ0)	SPRC				
00000004416	IPI00573738	88.9%	5.0^-120^	Ovalbumin-reated protein Y *	0.43
(G1MZF4)	OVALY				
00000013361	IPI00595705	98.4%	1.0^-54^	Similar to epidymal secretory protein E1 *	0.43
(G1NJ71)	Q5ZJI7				
00000016597	IPI00822809	77.8%	7.5^-22^	Meleagrin (gallin)	0.42
(G1NRF9)	DGGR58				
00000004408	IPI00570865	94.4%	2.4^-66^	Similar to REG4 *	0.38
(G1MZE6)	E1BZV4				
00000007908	IPI00578482	85.6%	1.8^-121^	Similar to α-carbonic anhydrase *	0.35
(G1N6Y5)	E1C004				
00000014942	IPI00604279	93.9%	1.8^-120^	Clusterin **	0.31
(G1NMV6)	Q9YGP0				
000000013859	IPI00680520	93.7%	1.9^-210^	Similar to pantetheinase/VNN1 *	0.29
(G1NKC5)	E1BUA6				
00000001205	IPI00603434	99.0%	1.7^-137^	Dentin matrix protein 4/FAM20C *+	0.27
(G1MSD1)	E1C4X0				
00000016580	IPI00583184	97.3%	1.8^-143^	Tsukushin *	0.25
(G1NRF2)	TSK				
00000010021	IPI00600561	81.2%	0	Chordin *+	0.24
(G1NBK1)	O57465				
00000007021	IPI00573727	99.3%	1.2^-115^	Similar to nucleobindin-2 **+	0.23
(G1N538)	Q5ZHR1				
00000008705	IPI00600003	95.2%	1.4^-168^	Cathepsin D **+	0.22
(G1N8P0)	CATD				
00000015671	IPI00573323	90.0%	2.5^-85^	Lysozyme G *	0.21
(G1NPK6)	LYG				
00000013696	IPI00585437	89.8%	1.9^-47^	Similar to prostate stem cell antigen *	0.18
(G1NJY7)	F1NXM7				
0000018441	IPI00594564	88.5%	1.1^-133^	Similar to nexin-1/serpin E2 **	0.18
(G3URX3)	E1BWU2				
00000009949	IPI00577639	93.9	1.2^-17^	Serpin G1/similar to plasma protease C1 inhibitor *	0.16
(G1NBE9)	F1NA58				
	(partial)				
00000007635	IPI00585901	93.9%	1.7^-84^	Osteopontin *+	0.15
(G1N6D8)	OSTP				
00000008830	IPI00585604	86.3%	0	Similar to mucin 5AC *	0.15
(G1N8Z1)	E1C037				
etc.	etc.				
00000010418	IPI00573327	94.2%	1.5^-208^	Vitamin D-binding protein **	0.15
(G1NCF1)	Q9W6F5				
00000003557	IPI00585935	98.0%	0	Similar to semaphorin-3G, fragment *	0.15
(G1MXI6)	F1NQ93				
00000010338	IPI00598054	93.1%	2.5^-66^	Similar to immunoglobin J chain **	0.15
(G1NC90)	E1BY93				
00000003137	IPI00590535	98.4%	0	Fibronectin, fragment **+	0.13
(G1MWJ4)	F1NJT3				
00000001542	IPI00587849	94.4%	6.1^-154^	Similar to antithrombin/serpin C1 *	0.12
(G1MT40)	F1NLP				
00000002860	IPI00577696	99.0%	0	Uncharacterized protein/similar to glypican-4 **+	0.12
(G1MVZ0)	F1NAU1				
00000006152	IPI00574055	99.0%	0	Procollagen-lysine 2-oxoglutarate 5-dioxygenase *	0.12
(G1N380)	PLOD1				
0000016417	IPI00581002	100%	8.0^-58^	Ubiquitin/polyubiquitin **	0.12
(G1NR52)	F1N4V4				
	etc.				
00000000900	IPI00595826	99.7%	0	Golgi apparatus protein 1 *+	0.11
(G1MRQ6)	F1P250				
	etc.				

This table contains all proteins sharing >0.1% of the total proteome. *, indicates previous identification in the chicken eggshell. **, indicates previous identification among the 50 most abundant proteins in the chicken eggshell. For the complete list of identified proteins and details see (Additional file 1: Table S1). **+**, indicates proteins the message of which was shown to be up-regulated in egg-containing uterus. ^1^, the average of two biological replicates (Additional file 1: Table S1). ^2^, immunoglobulins with largely overlapping peptide sets were grouped together. The 47 proteins or protein groups shown in this table represent 95% of the total identified turkey eggshell matrix proteome.

### Ovocleidins and ovocalyxins (“eggshell-specific” proteins)

**Figure 3:**
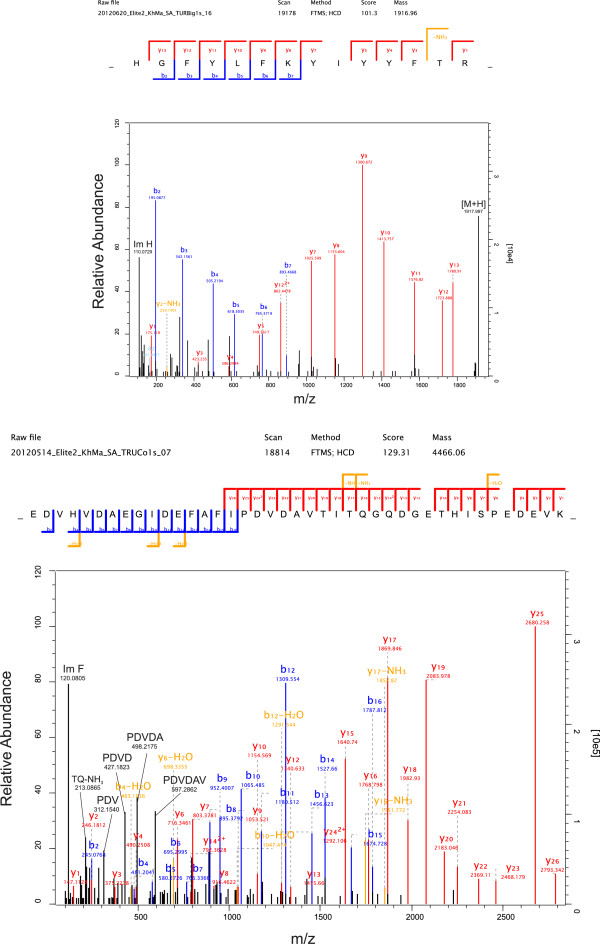
**Representative spectra of turkey ovocleidin-116 (ENSMGAP00000007641/G1N6E1) peptides.** Top, doubly charged peptide (aa46-59) with PEP 2.4^-15^ and a mass error of −1.0 ppm. Bottom, quintuply charged peptide (aa234-273) with PEP 8.5^-215^ and a mass error of −0.2 ppm. MaxQuant Expert System annotations not contained in simple fragment annotation were added for some major peaks (in black). These were the [M + H] ion and the histidine immonium ion in the upper spectrum and a series of internal fragments and the phenylalanine immonium ion in the lower spectrum. Full expert annotation is not shown for sake of clarity.

**Figure 4:**
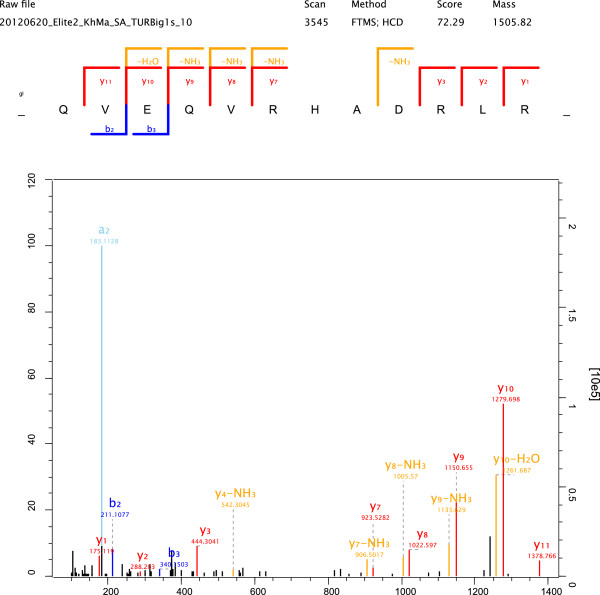
**Spectrum of a turkey OC-116 peptide indicating the presence of a C-terminus similar to chicken OC-116.** This peptide was not found in turkey OC-117 but matches the chicken OC-116 C-terminus (aa671-682), which is missing in the turkey protein. This quadruply charged peptide was identified with a posterior error probability (PEP) of 0.0005 and a mass error of −0.144 ppm. It is the only evidence for the presence in the turkey protein of a C-terminus similar to that of chicken OC-116. The N-terminal glutamine was cyclized to pyroglutamate during peptide fractionation under acidic conditions, a frequently observed modification of peptides with N-terminal glutamine.

By far the most abundant protein in the identified turkey eggshell proteome was ovocleidin-116 (OC-116) (Table 1). As frequently observed for major proteins, OC-116 was identified with hundreds of peptides all over the PAGE gradient (Additional file 2: Table S2 and Additional file 3: Table S3). However, we found the highest concentration in slice 5 (Figure 1) corresponding to a M_r_ of 80 to 110 kDa. The presence of OC-116 fragments, observed in chicken matrix preparations [37], could also have contributed to the PAGE pattern and the resulting peptide distribution. Some representative spectra identifying and distinguishing the turkey protein are shown in Figure 3. OC-116 was first detected in chicken eggshell as the core protein of a proteoglycan with a molecular mass of approximately 120kDa [38] and was subsequently characterized by molecular cloning and sequencing [37,39]. A substantial fraction of it occurs in the eggshell matrix without attached glycosaminoglycan chains, but in an N-glycosylated form [40]. In the chicken eggshell matrix we previously showed that OC-116 was one of the most abundant proteins of the acid-soluble proteome [8] and phosphoproteome [9]. OC-116 was first described as an eggshell-specific protein [39] but was subsequently also identified in egg yolk [41], the vitelline membrane [42] and egg white [43]. Interestingly, however, it was also identified in chicken bone [44] and is expressed in chicken osteoblasts and osteocytes during bone development and mineralization [45], indicating some similarities between bone and eggshell mineralization. The supposed mammalian homolog of OC-116, matrix extracellular phosphoglycoprotein (MEPE) [45], was shown to regulate bone formation, for instance by inhibiting growth plate cartilage mineralization [46]. In comparison to chicken OC-116 the turkey protein sequence (ENSMGAP00000007641/G1N6E1_MELGA) lacked approximately 250 amino acids (aa) at the C-terminus. Sequence identity in the remaining overlap of approximately 500aa was 80%. Consequently only four of the 40 sequence–unique peptides identified matched to chicken and turkey sequences. Database searching after we had added the chicken OC-116 sequence to the turkey sequence database produced a single peptide, _670_QVEQVRHADRLR_682_, matching to the C-terminus (Figure 4). Compared to other OC-116 peptides this peptide was rarely identified and occurred only in very few technical replicates. The distribution of OC-116 peptides over the PAGE slices would rather indicate a turkey OC-116 of a length similar to that of the chicken protein.

**Figure 5:**
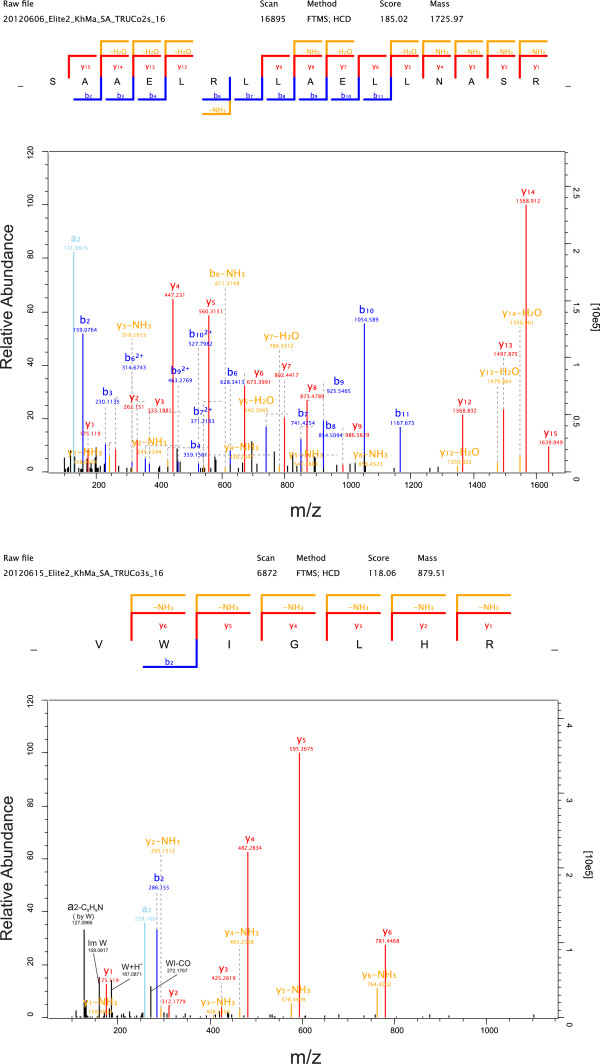
**Spectra indicating the presence of OC-17 in the turkey eggshell proteome.** This figure shows selected spectra of the two ovocleidin-17 peptides. Top, this triply charged peptide was identified with a mass error of 0.7 ppm and a PEP of 1.9^-102^. Bottom, doubly charged peptide with a mass error of 0.3 ppm and a PEP of 0.01. Four MaxQuant Expert System annotations for major peaks are shown in black in addition to the simple fragment annotation. These were two internal fragments, the immonium ion of tryptophane, and an a2 ion with loss of the tryptophane residue from the peptide backbone. Full expert annotation was omitted for sake of clarity.

Ovocleidin-17 (OC-17) is a major chicken eggshell protein [8] completely unrelated to OC-116 [47,48]. Its amino acid sequence was established by Edman sequence analysis of the purified protein [48]. The presence of a C-type lectin-like domain lacking the characteristics of a typical true C-type lectin and a tendency to aggregate during isolation suggested a function as structural matrix protein [48]. However, OC-17 was also reported to affect *in vitro* calcium carbonate crystallization [49] and to possess antimicrobial activity [50]. Neither the genome-derived protein sequence database of turkey nor that of chicken contained a sequence of significant similarity to OC-17, indicating that it may be encoded in the 5-10% of the genome sequences still missing for both species [23]. Addition of the chicken OC-17 protein sequence to the turkey protein sequence database yielded two OC-17 peptides, _47_SAAELRLLAELLNASR_62_ and _75_VWIGLHR_81_ (Figure 5). Furthermore, the existence of a protein similar to OC-17 in turkey eggshell matrix would be in agreement with the results of a comparative Western blotting study using anti-chicken OC-17 antiserum [51]. The OC-17 peptides peaked in gel fractions 15 and 16 (Additional files 2: Table S2 and Additional file 3: Table S3), corresponding to the size of OC-17. However, major proteins of similar size, such as avidin and cystatin, also showed a peak of their peptide distribution in these same fractions and very likely correspond to the two major bands observed in these sections (Figure 1). Nevertheless, the sum of evidence may indicate that OC-17 is present in turkey eggshell matrix, although at an unknown percentage of the total proteome.

Ovocalyxin-36 (OCX-36) is a major protein of the turkey eggshell matrix proteome (Table 1) and the chicken eggshell matrix proteome [8]. In chicken it is secreted in oviduct sections where eggshell production takes place and its gene expression is strongly up-regulated during mineralization [19,52]. Its sequence contains a bactericidal permeability-increasing domain and OCX-36 was therefore suggested to play a role in egg defense against microbial contamination [52].

Two other ovocalyxins, OCX-32 and OCX-21 were not found in the turkey eggshell matrix proteome. OCX-32 [53] apparently did not have a counterpart in the published turkey genome sequence. Addition of the chicken protein sequence to the turkey sequence database used with MaxQuant did not produce any evidence for its presence in the turkey eggshell matrix. OCX-21 is a name given to the sequence contained in entry IPI00574331 of the chicken sequence database on various occasions [17,19]) and is identical to gastrokine-2 (E1C2G7_CHICK), a secretory protein of mammalian gastric surface mucous cells [54]. FASTA database searches showed that the turkey protein sequence database did contain a homolog of the protein with 92.5% sequence identity to the chicken protein in accession ENSMGAP00000000035/G1MPS6 _MELGA. Therefore, the absence of any identified peptide of this major chicken eggshell matrix protein [8] may indicate its absence from the turkey eggshell matrix.

### Major proteins not previously identified in eggshells

Among the major turkey eggshell matrix proteins (Table 1) were two proteins that were not identified in other eggshell matrices or egg fractions before, periostin and trefoil family peptide 2 (TFF-2). Periostin accounted for almost 11% of the total matrix proteome and was not identified in the chicken matrix although a periostin sequence was present in the IPI_CHICK sequence database used for this study [8]. As expected from its name, periostin was detected in the periosteum that covers the outer surface of bone, but it also occurs in other collagen-rich mammalian connective tissues [55]. The role of periostin in bone mineralization is not clear at present, although it seems to be important for bone growth and repair, and there is no indication of its possible function in eggshell formation at present. Its presence in shell matrix now provides another strong link of eggshell mineralization to bone metabolism.

TFF-2 belongs to a family of small proteins expressed predominantly in the mucosa of the gastrointestinal tract [56,57] and it seems to play a role in mucosal protection and repair. Its location and function in the mammalian gastrointestinal tract apparently partially overlaps with that of gastrokines such as OCX-21/gastrokine-2. Indeed interactions between gastrokines and TFFs have been reported [54]. TFF-2 binds to mucins, especially to mucin 5AC [58], which was another major component of the major turkey eggshell (Table 1). *In vitro* binding of TFF-2 to mucin induces the formation of highly viscous complexes [59]. Thus, the function of these two matrix components may be to protect the shell gland mucosa of egg-laying hens.

Also not previously identified in eggshell matrix was the egg white protein meleagrin [60]. Its chicken homolog gallin was shown to have antimicrobial activity [61]. The expression of gallin was highest in the magnum section of the oviduct and about 140 times less in the eggshell gland. This indicates that this small protein reaches the eggshell gland essentially as a left-over of egg white assembly by co-migration through the oviduct together with the unfinished egg [61].

### Other major proteins possibly involved in mineralization or matrix assembly of chicken and turkey

Osteopontin is a major non-collagen protein in bone but also occurs in many other tissues and body fluids. It has an inhibitory effect on various normal and pathological mineralization processes [62]. In the chicken oviduct it is secreted exclusively in the eggshell gland and accumulates in the shell matrix [13]. Osteopontin of different species is highly phosphorylated and its inhibitory effect was shown to depend on phosphorylation. Similar to chicken osteopontin [8,9] the abundance of turkey osteopontin was most probably greatly underestimated because the phosphorylated peptides were not identified in this general survey. The kinase phosphorylating extracellular matrix proteins such as OC-116 and osteopontin was recently identified as FAM20C protein [63,64], a Golgi lumen resident that was also found in chicken and turkey eggshell matrix (Table 1). Another obvious candidate for a protein with a role in mineralization is G1N6Y5_MELGA, which contains a predicted α-carbonic anhydrase domain and could therefore be involved in carbonate production for the growing eggshell calcite layer or the control of CO_2_ concentration in the uterus fluid. A protein with a probable, yet unknown, function in eggshell assembly is glypican-4 (Table 1). Similar to the expression of osteopontin [65] its expression is massively up-regulated in response to the mechanical strain exerted onto the eggshell gland walls upon entry of the egg and ceases shortly before completion of the eggshell [66]. Other proteins may have a more general function in eggshell matrix production, such as the extracellular chaperone clusterin, which may be important in maintaining the proper folding of matrix proteins during matrix assembly [67]. SPARC/BM40/osteonectin, which is also abundant in other vertebrate mineralized tissues such as bone and teeth, may participate in regulation of matrix assembly [68].

### Major egg white proteins in the eggshell matrix

Major egg white proteins known from chicken that were identified among the major turkey eggshell matrix proteins were ovalbumin, avidin, ovotransferrin, cystatin and lysozyme C (Table 1). Many others were identified at lower abundance (Additional file 1: Table S1). Two of the major proteins, lysozyme C and ovotransferrin were shown to be true components of the chicken eggshell matrix previous to proteomic analyses using immunohistochemical methods [11,12]. Several of these proteins have antimicrobial activity and their main function during egg production may be in the defense of egg and oviduct against microbial contamination either by direct attack of bacterial cell walls (lysozyme C), iron sequestration (ovotransferrin), biotin sequestration (avidin), or protease inhibition (cystatin) [1,2]. Lysozyme C, ovotransferrin, and ovalbumin have a very weak effect on calcium carbonate crystal morphology *in vitro*[11,12,14,15] but their possible role in *in vivo* crystallization, if any, remains unclear at present. An attractive idea in this respect is that such proteins showing only very weak or no interaction with calcite may nevertheless influence eggshell mineralization by maintaining a proper environment in the eggshell gland with respect to pH, CO_2_ concentration and soluble calcium availability in the uterus fluid [14].

## Conclusions

Analysis of the turkey eggshell matrix proteome revealed some unexpected differences as compared to the chicken eggshell matrix, although both species belong to the same family (Phasianidae). The turkey eggshell contains a new major eggshell component, the bone protein periostin. There were also differences among a group of so-called eggshell-specific proteins produced in the chicken eggshell gland epithelial cells and thought to be very important for eggshell production. Two of these, OC-116 and OCX-36, were also among the major turkey eggshell matrix proteins. Another one, OCX-21/gastrokine-2 was missing in the proteome, although a very similar protein sequence was contained in the genome-derived turkey protein sequence database. The sequences of OC-17 and OCX-32 were not contained in the turkey sequence database but may be encoded in the 5-10% of the genome not yet sequenced [22,23]. Addition of the chicken sequences of these two proteins to the database enabled identification of two OC-17 peptides suggesting the presence of this protein in the turkey eggshell matrix. However, this approach was not successful in the case of OCX-32, indicating that it either was not in the matrix or that the sequences are too different for such a cross-species identification. More eggshell proteomes are needed to identify a possible common set of shell mineralization-controlling avian proteins along with more transcriptome studies, immunochemical approaches to eggshell protein localization, and functional tests with isolated proteins.

## Abbreviations

Aa: Amino acid; FDR: False discovery rate; HCD: Higher-energy collision-induced dissociation; iBAQ: Intensity-based absolute quantification; OC: Ovocleidin; OCX: Ovocalyxin; PAGE: Polyacrylamide gel electrophoresis; PEP: Posterior error probability; TFF: Trefoil family of proteins.

## Competing interests

The authors declare that they have no competing interests.

## Authors’ contributions

KM conceived the study, performed sample preparation and data acquisition. MM supplied methodological expertise. Both authors took part in the design of the study and were critically involved in manuscript drafting. All authors read and approved the final manuscript.

## Supplementary Material

Additional file 1: Table S1 This table shows the complete list of accepted protein identifications in turkey eggshell matrix preparations. Click here for file

Additional file 2: Table S2 ProteinGroups pool A. This file shows the MaxQuant-derived protein identification data for pool A (strain Converter), such as additional accession numbers for protein groups, protein scores, number of peptides, distribution of peptides in gel slices and molecular weight of the predicted protein. Click here for file

Additional file 3: Table S3 ProteinGroups pool B. MaxQuant-derived protein data for pool B (strain Big6). Click here for file

Additional file 4: Table S4 Peptides pool A. MaxQuant list of peptides identified in pool A (strain Converter). This Excel sheet contains all peptide sequences in alphabetic order and relevant parameters, such as preceding amino acid, peptide charge, peptide mass, scores, PEP value, distribution in gel slices, and iBAQ intensities. Click here for file

Additional file 5: Table S5 Peptides pool B. MaxQuant list of peptides identified in pool B (strain Big6). Click here for file

## References

[B1] Burley RW Vadhera DV The avian egg. Chemistry and Biology 1989 New York: John Wiley and Sons

[B2] Mine Y Egg bioscience and biotechnology 2008 Hoboken, New Jersey: John Wiley and Sons

[B3] Schneider WJ Osanger A Waclawek M Nimpf J Oocyte growth in the chicken. Receptors and more Biol Chem 1998 379 965 971 9792429

[B4] Creger CR Phillips H Scott JT Formation of an eggshell Poult Sci 1976 55 1717 1723 10.3382/ps.0551717

[B5] Rose MLH Hincke MT Protein constituents of the eggshell: eggshell-specific matrix proteins Cell Mol Life Sci 2009 66 2707 2719 10.1007/s00018-009-0046-y 19452125 PMC11115492

[B6] Hincke MT Nys Y Gautron J The role of matrix proteins in eggshell formation J Poult Sci 2010 47 208 219 10.2141/jpsa.009122

[B7] Hincke MT Nys Y Gautron J Mann K Rodriguez-Navarro AB McKee MD The eggshell: structure, composition and mineralization Front Biosci 2012 17 1266 1280 10.2741/3985 22201802

[B8] Mann K Macek B Olsen JV Proteomic analysis of the acid-soluble organic matrix of the chicken calcified eggshell layer Proteomics 2006 6 3801 3810 10.1002/pmic.200600120 16767793

[B9] Mann K Olsen JV Macek B Gnad F Mann M Phosphoproteins of the chicken eggshell calcified layer Proteomics 2007 7 106 115 10.1002/pmic.200600635 17152097

[B10] Hincke MT Ovalbumin is a component of the chicken eggshell matrix Connect Tissue Res 1995 31 227 233 10.3109/03008209509010814 15609630

[B11] Hincke MT Gautron J Panheleux M Garcia-Ruiz J McKee MD Nys Y Identification and localization of lysozyme as a component of eggshell membranes and eggshell matrix Matrix Biol 2000 19 443 453 10.1016/S0945-053X(00)00095-0 10980420

[B12] Gautron J Hincke MT Panhéleux M Garcia-Ruiz JM Boldicke T Nys Y Ovotransferrin is a matrix protein of the hen eggshell membranes and basal calcified layer Connective Tissue Res 2001 42 255 267 10.3109/03008200109016840 11913770

[B13] Pines M Knopov V Bar A Involvement of osteopontin in egg shell formation in the laying chicken Matrix Biol 1994 14 765 771 8785591 10.1016/s0945-053x(05)80019-8

[B14] Hernández-Hernández A Gómez-Morales J Rodríguez-Navarro AB Gautron J Nys Y García-Ruiz JM Identification of some active proteins in the process of hen eggshell formation Crystal Growth & Design 2008 8 4330 4339 10.1021/cg800786s23996829

[B15] Hernández-Hernández A Vidal M-L Gómez-Morales J Rodríguez-Navarro AB Labas V Gautron J Nys Y García-Ruiz JM Influence of eggshell matrix proteins on the precipitation of calcium carbonate Journal of Crystal Growth 2008 310 1754 1759 10.1016/j.jcrysgro.2007.11.170

[B16] Miksik I Sedlakova P Lacinova K Pataridis S Eckardt A Determination of insoluble avian eggshell matrix proteins Anal Bioanal Chem 2010 397 205 214 10.1007/s00216-009-3326-3 19998026

[B17] Rose-Martel M Du J Hincke MT Proteomic analysis provides new insight into the eggshell cuticle J Proteom 2012 75 2697 2706 10.1016/j.jprot.2012.03.019 22708129

[B18] Dunn IC Wilson PW Lu Z Bain MM Crossan CL Talbot RT Waddington D New hypotheses on the function of the avian shell gland derived from microarray analysis comparing tissue from juvenile and sexually mature hens Gen Comp Endocrinol 2009 163 225 232 10.1016/j.ygcen.2009.03.006 19303879

[B19] Jonchère V Réhaut-Godbert S Hennequet-Antier C Cabau C Sibut V Cogburn LA Nys Y Gautron J Gene expression profiling to identify eggshell proteins involved in physical defence of the chicken egg BMC Genomics 2010 11 57 10.1186/1471-2164-11-57 20092629 PMC2827412

[B20] International Chicken Genome Sequencing Consortium Sequence and comparative analysis of the chicken genome provide unique perspectives on vertebrate evolution Nature 2004 432 695 716 10.1038/nature03154 15592404

[B21] Warren WC Clayton DF Ellegren H Arnold AP Hillier LD Künstner A Searle S White S Vilella AJ Fairley S Heger A Kong L Ponting CP Jarvis ED Mello CV Minx P Lovell P Velho TAF Ferris M Balakrishnan CN Sinha S Blatti C London SE Li Y Lin Y-C George J Sweedler J Southey B Gunaratne P Watson M Nam K Backström N Smeds L Nabholz B Itoh Y Whitney O Pfenning AR Howard J Völker M Skinner BM Griffin DK Ye L McLaren WM Flicek P Quesada V Velasco G Lopez-Otin C Puente XS Olender T Lancet D Smit AFA Hubley R Konkel MK Walker JA Batzer MA Gu W Pollock DD Chen L Cheng Z Eichler EE Stapley J Slate J Ekblom R Birkhead T Burke T Burt D Scharff C Adam I Richard H Sultan M Soldatov A Lehrach H Edwards SV Yang S-P Li XC Graves T Fulton L Nelson J Chinwalla A Hou S Mardis ER Wilson RK The genome of a songbird Nature 2010 464 757 762 10.1038/nature08819 20360741 PMC3187626

[B22] Dalloul RA Long JA Zimin AV Aslam L Beal K Blomberg L Bouffard P Burt DW Crasta O Crooijmans RPMA Cooper K Coulombe RA De S Delany ME Dodgson JB Dong JJ Evans C Frederickson KM Flicek P Florea L Folkerts O Groenen MAM Harkins TT Herrero J Hoffmann S Megens HJ Jiang A de Jong P Kaiser P Kim H Kim KW Kim S Langenberger D Lee MK Lee T Mane S Marcais G Marz M McElroy AP Modise T Nefedov M Notredame C Paton IR Payne WS Pertea G Prickett D Puiu D Qioa D Raineri E Ruffier M Salzberg SL Schatz MC Scheuring C Schmidt CJ Schroeder S Searle SMJ Smith EJ Smith J Sonstegard TS Stadler PF Tafer H Tu ZJ Van Tassell CP Vilella AJ Williams KP Yorke JA Zhang LQ Zhang HB Zhang XJ Zhang Y Reed KM Multi-platform next-generation sequencing of the domestic turkey (Meleagris gallopavo): genome assembly and analysis PLOS Biology 2010 8 e1000475 10.1371/journal.pbio.1000475 20838655 PMC2935454

[B23] Dodgeson JB Delany ME Cheng HH Poultry genome sequences: progress and outstanding challenges Cytogenet Genome Res 2011 134 19 26 10.1159/000324413 21335957

[B24] Shevchenko A Tomas H Havlis J Olsen JV Mann M In-gel digestion for mass spectrometric characterization of proteins and proteomes NatureProtocols 2006 1 2856 2860 10.1038/nprot.2006.468 17406544

[B25] Rappsilber J Mann M Ishihama Y Protocol for micro-purification, enrichment, pre-fractionation and storage of peptides for proteomics using StageTips Nat Protoc 2007 2 1896 1906 10.1038/nprot.2007.261 17703201

[B26] Michalski A Damoc E Lange O Denisov E Nolting D Müller M Viner R Schwartz J Remes P Belford M Dunyach J-J Cox J Horning S Mann M Makarov A Ultra high resolution linear ion trap orbitrap mass spectrometer (Orbitrap Elite) facilitates top down LC MS/MS and versatile peptide fragmentation modes Mol Cell Proteomics 2012 11 1 11 10.1074/mcp.E112.019653 22159718 PMC3316736

[B27] Michalski A Neuhauser N Cox J Mann M A systematic investigation into the nature of tryptic HCD spectra J Proteome Res 2012 11 5479 5491 10.1021/pr3007045 22998608

[B28] Cox J Mann M MaxQuant enables high peptide identification rates, individualized ppb-range mass accuracies and proteome-wide protein quantification Nature Biotechnol 2009 26 1367 1372 19029910 10.1038/nbt.1511

[B29] Cox J Matic I Hilger M Nagaraj N Selbach M Olsen JV Mann M A practical guide to the MaxQuant computational platform for SILAC-based quantitative proteomics Nat Protoc 2009 4 698 705 19373234 10.1038/nprot.2009.36

[B30] Cox J Neuhauser N Michalski A Scheltema RA Olsen JV Mann M Andromeda – a peptide search engine integrated into the MaxQuant environment J Proteome Res 2011 10 1794 1805 10.1021/pr101065j 21254760

[B31] Neuhauser N Michalski A Cox J Mann M Expert system for computer-assisted annotation of MS/MS spectra Mol Cell Proteom 2012 11 1500 1509 10.1074/mcp.M112.020271 PMC3494176 22888147

[B32] Schwanhäusser B Busse D Li N Dittmar G Schuchhardt J Wolf J Chen W Selbach M Global quantification of mammalian gene expression control Nature 2011 473 337 342 10.1038/nature10098 21593866

[B33] Goujon M McWilliam H Li W Valentin F Squizzato S Paern J Lopez R A new bioinformatics analysis tools framework at EMBL-EBI (2010) Nucleic Acids Res 2010 38 Suppl W695 9 20439314 10.1093/nar/gkq313 PMC2896090

[B34] Hunter S Jones P Mitchell A Apweiler R Attwood TK Bateman A Bernard T Binns D Bork P Burge S de Castro E Coggill P Corbett M Das U Daugherty L Duquenne L Finn RD Fraser M Gough J Haft D Hulo N Kahn D Kelly E Letunic I Lonsdale D Lopez R Madera M Maslen J McAnulla C McMenamin C Mi H Mutowo-Muellenet P Mulder N Natale D Orengo C Pesseat S Punta M Quinn AF Rivoire C Sangrador-Vegas A Selengut JD Sigrist CJA Scheremetjew M Tate J Thimmajanarthanan M Thomas PD Wu CH Yeats C Yong S-Y InterPro in 2011: new developments in the family and domain prediction database Nucleic Acids Res 2011 40 D306 D312 22096229 10.1093/nar/gkr948 PMC3245097

[B35] Petersen TN Brunak S von Heinje G Nielsen H SignalP 4.0: discriminating signal peptides from transmembrane regions Nat Methods 2011 8 785 786 10.1038/nmeth.1701 21959131

[B36] Ishihama Y Oda Y Tabata T Sato T Nagasu T Rappsilber J Mann M Exponentially modified protein abundance index (emPAI) for estimation of absolute protein amount in proteomics by the number of sequenced peptides per protein Mol Cell Proteomics 2005 4 1265 1272 10.1074/mcp.M500061-MCP200 15958392

[B37] Mann K Hincke MT Nys Y Isolation of ovocleidin-116 from chicken eggshells, correction of its amino acid sequence and identification of disulfide bonds and glycosylated Asn Matrix Biol 2002 21 383 387 10.1016/S0945-053X(02)00031-8 12225802

[B38] Carrino DA Rodriguez JP Caplan AI Dermatan sulfate proteoglycans from the mineralized matrix of the avian eggshell Connect Tissue Res 1997 36 175 193 10.3109/03008209709160219 9512887

[B39] Hincke MT Gautron J Tsang CP McKee MD Nys Y Molecular cloning and ultrastructural localization of the core protein of an eggshell matrix proteoglycan, ovocleidin-116 J Biol Chem 1999 274 32915 32923 10.1074/jbc.274.46.32915 10551857

[B40] Nimtz M Conradt HS Mann K LacdiNAc (GalNAcβ1-4GlcNAc) is a major motif in N-glycan structures of the chicken eggshell protein ovocleidin-116 Biochim Biophys Acta 2004 1675 71 80 10.1016/j.bbagen.2004.08.007 15535969

[B41] Mann K Mann M The chicken egg yolk plasma and granule proteomes Proteomics 2008 8 178 191 10.1002/pmic.200700790 18046696

[B42] Mann K Proteomic analysis of the chicken egg vitelline membrane Proteomics 2008 8 2322 2332 10.1002/pmic.200800032 18452232

[B43] Mann K Mann M In-depth analysis of the chicken egg white proteome using an LTQ orbitrap Velos Proteome Sci 2001 9 7 21299891 10.1186/1477-5956-9-7 PMC3041730

[B44] Horvat-Gordon M Yu F Burns D Leach RM Ovocleidin (OC 116) is present in avian skeletal tissues Poult Sci 2008 87 1618 1623 10.3382/ps.2008-00031 18648057

[B45] Bardet C Vincent C Lajarille M-C Jaffredo T Sire J-Y OC-116, the chicken ortholog of mammalian MEPE found in eggshell, is also expressed in bone cells J Exp Zool 2010 314B 653 662 10.1002/jez.b.21366 20665709

[B46] Staines KA Mackenzie NCW Clarkin CE Zelenchuk L Rowe PS MacRae VE Farquharson C MEPE is a novel regulator of growth plate cartilage mineralization Bone 2012 51 418 430 10.1016/j.bone.2012.06.022 22766095 PMC3427007

[B47] Hincke MT Tsang CPW Courtney M Hill V Narbaitz R Purification and immunochemistry of a soluble matrix protein of the chicken eggshell (ovocleidin-17) Calcif Tissue Int 1995 56 578 583 10.1007/BF00298593 7648490

[B48] Mann K Siedler F The amino acid sequence of ovocleidin-17, a major protein of the avian eggshell calcified layer Biochem Mol Biol Int 1999 47 997 1007 10410246 10.1080/15216549900202123

[B49] Reyes-Grajeda JP Moreno A Romero A Crystal structure of ovocleidin-17, a major protein of the calcified Gallus gallus eggshell J Biol Chem 2004 279 40876 40881 10.1074/jbc.M406033200 15263013

[B50] Wellman-Labadie O Lakshminarayanan R Hincke MT Antimicrobial properties of avian eggshell-specific C-type lectin-like proteins FEBS Lett 2008 582 699 704 10.1016/j.febslet.2008.01.043 18258195

[B51] Panhéleux M Bain M Fernandez MS Morales I Gautron J Arias JL Solomon SE Hincke MT Nys Y Organic matrix composition and ultrastructure of eggshell: a comparative study Brit Poult Sci 1999 40 240 252 10.1080/00071669987665 10465392

[B52] Gautron J Murayama E Vignal A Morisson M McKee MD Réhault S Labas V Belghazi M Vidal M-L Nys Y Hincke MT Cloning of ovocalyxin-36, a novel chicken eggshell protein related to lipopolysaccharide-binding proteins, bactericidal permeability-increasing proteins, and Plunc family proteins J Biol Chem 2007 282 5273 5286 17179153 10.1074/jbc.M610294200

[B53] Gautron J Hincke MT Mann K Panhéleux M Bain M McKee MD Solomon SE Nys Y Ovocalyxin-32, a novel chicken eggshell matrix protein J Biol Chem 2001 276 39243 39252 10.1074/jbc.M104543200 11493603

[B54] Menheniott TR Kurklu B Giraud AS Gastrokines: stomach-specific proteins with putative homeostatic and tumor suppressor roles Am J Physiol Gastrointest Liver Physiol 2013 304 G109 G121 10.1152/ajpgi.00374.2012 23154977

[B55] Merle B Garnero P The multiple facets of periostin in bone metabolism Osteoporos Int 2012 23 1199 1212 10.1007/s00198-011-1892-7 22310955

[B56] Kjellev S The trefoil factor family – small peptides with multiple functionalities Cell Mol Life Sci 2009 66 1350 1369 10.1007/s00018-008-8646-5 19099184 PMC11131466

[B57] Jiang Z Lossie AC Applegate TJ Evolution of trefoil factor(s): Genetic and spatio-temporal expression of trefoil factor 2 in the chicken (*Gallus gallus domesticus*) PlosOne 2001 6 e22691 10.1371/journal.pone.0022691 PMC3146476 21829480

[B58] Kouznetsova I Laubinger W Kalbacher H Kalinski T Meyer F Roessner A Hoffman W Biosynthesis of gastrokine-2 in the human gastric mucosa: restricted spatial expression along the antral gland axis and differential interaction with TFF1, TFF2 and mucins Cell Physiol Biochem 2007 20 899 908 10.1159/000110450 17982272

[B59] Thim L Madsen F Poulsen SS Effect of trefoil factors on the viscoelastic properties of mucus gels Eur J Clin Invest 2002 32 519 527 10.1046/j.1365-2362.2002.01014.x 12153553

[B60] Odani S Koide T Ono T Takahashi Y Suzuki J Covalent structure of a low-molecular mass protein, meleagrin, present in turkey (*Meleagris gallopavo*) ovomucoid preparation J Biochem 1989 105 660 663 2760022 10.1093/oxfordjournals.jbchem.a122721

[B61] Gong D Wilson PW Bain MM McDade K Kalina J Hérve-Grépinet V Nys Y Dunn IC Gallin, an antimicrobioal peptide member of a new avian defensing family, the ovodefensins, has been subject to recent gene duplication BMC Immunol 2010 11 12 10.1186/1471-2172-11-12 20226050 PMC2846878

[B62] Staines KA MacRae VE Farquharson C The importance of the SIBLING family of proteins on skeletal mineralization and bone remodeling J Endocrinol 2012 214 241 255 10.1530/JOE-12-0143 22700194

[B63] Ishikawa HO Xu A Ogura E Manning G Irvine KD The Raine syndrome protein FAM20C is a Golgi kinase that phosphorylates biomineralization proteins PLOS ONE 2012 7 e42988 10.1371/journal.pone.0042988 22900076 PMC3416761

[B64] Tagliabracci VS Engel JL Wen J Wiley SE Worby CA Kinch LN Xiao J Grishin NV Dixon JE Secreted kinase phosphorylates extracellular proteins that regulate biomineralization Science 2012 336 1150 1153 10.1126/science.1217817 22582013 PMC3754843

[B65] Lavelin I Yarden N Ben-Bassat S Bar A Pines M Regulation of osteopontin expression during egg shell formation in the laying hen by mechanical strain Matrix Biol 1998 17 615 623 10.1016/S0945-053X(98)90112-3 9923654

[B66] Lavelin I Meiri N Einat M Genina O Pines M Mechanical strain regulation of the chicken glypican-4 gene expression in the avian eggshell gland Am J Physiol Regul Integr Comp Physiol 2002 283 R855 R861 10.1152/ajpregu.00088.2002 12228054

[B67] Mann K Gautron J Nys Y McKee MD Bajari T Schneider WJ Hincke MD Disulfide-linked heterodimeric clusterin is a component of the chicken eggshell matrix and egg white Matrix Biol 2003 22 397 407 10.1016/S0945-053X(03)00072-6 14614987

[B68] Kawasaki K Buchanan AV Weiss KM Biomineralization in humans: Making the hard choices of life Annu Rev Genet 2009 43 119 142 10.1146/annurev-genet-102108-134242 19659443

