# Supplementary material for: The proteome of the calcified layer organic matrix of turkey (Meleagris gallopavo) eggshell
Source: Proteome Sci. 2013 Aug 27;11:40. doi: 10.1186/1477-5956-11-40 (PMC3766105; doi:10.1186/1477-5956-11-40)
Supplement: Additional file 1: Table S1 — This table shows the complete list of accepted protein identifications in turkey eggshell matrix preparations. [file 1477-5956-11-40-S1.docx]

**Table 1**

**Turkey eggshell matrix proteins**

| **ENSMGAP**  **accession no.**  **(UniProt)** | **Chicken homolog**  **(>75% identity)** | **Protein** | **Pool** | | **Fraction** | **Unique**  **Pep-tides** | **% of total**  **(iBAQ)** | **mean** |
| --- | --- | --- | --- | --- | --- | --- | --- | --- |
|  |  |  |  | |  |  |  |  |
| 00000000004  (B1N1B6) | IPI00584841  Q8AV77; [8] | Hep21 | A  B | | S  - | 2  - | 0.0085  - |  |
| 00000000006  (B1N1C2) | IPI00596315  GBLP | Guanine nucleotide-binding protein subunit β2-like | A  B | | -  S | -  5 | -  0.0006 |  |
| 00000000018  (G1MPR2)  00000007974  (H9H1C2)  0000019295  (H9H2I1) | IPI00581158  Q90633; [8] | Similar to complement C3, fragments | A  B | | **S**,I  **S**,I | 17  19 | 0.0067  0.0048 | 0.0058 |
| 00000000098  (G1MPX8) | IPI00597864  PRIO | Major prion protein | A  B | | S  S | 3  2 | 0.0006  0.0007 | 0.0007 |
| 00000000099  (G1MPX9) | IPI00588294  F1NP62 | Uncharacterized protein; domains: SEA, peptidase S1/S6, LDL receptor repeat | A  B | | **S**,I  **S**,I | 10  11 | 0.0008  0.0009 | 0.0009 |
| 00000000116  (G1MPZ3) | IPI00593518  Q5F390; [8] | Uncharacterized protein; domains: Signal recognition particle (SRP) GTPase subunit, SRP α subunit, SRP54 subunit | A  B | | S,**I**  S,**I** | 5  7 | 0.0016  0.0024 | 0.0020 |
| 00000000142  (G1MQ16) | IPI00600069  EXFAB; [8] | Uncharacterized protein/extracellular fatty acid-binding protein (EXFAB) | A  B | | **S,**I  **S**,I | 19  15 | 3.8466  3.8024 | 3.8245 |
| 00000000221  (G1MQ78) | IPI00575913  F1P014; [8] | Uncharacterized protein; domains: Lipocalin/cytFAB/calycin, α1-microglobulin, signal seq | A  B | | **S**,I  **S**,I | 8  7 | 0.0574  0.0499 | 0.0537 |
| 00000000231  (G1MQ88/  Q6QA54) | IPI00575904  GPC1; [8] | Uncharacterized protein/Glypican-1 | A  B | | **S,**I  **S**,I | 15  17 | 0.0256  0.0316 | 0.0286 |
| 00000000237  (G1MQ93) | IPI00603559  E1C6Y2 | Uncharacterized protein; domain: peptidase S8/S53/subtilisin/kexin/sedolisin | A  B | | **S**,I  S | 6  8 | 0.0004  0.0007 | 0.0006 |
| 00000000288  (G1MQD8) | IPI00588684  IPI00593121  E1BZ81, [8] | Uncharacterized protein/similar to dipeptidyl peptidase 2; domain: Peptidase S8 | A  B | | **S**,I  **S,**I | 6  7 | 0.0052  0.0078 | 0.0065 |
| 00000000302  (G1MQF0) | IPI00586210  Q5QQ39 | Uncharacterized protein/similar to a-2,3-sialyltransferase ST3 Gal IV | A  B | | **S**,I  **S**,I | 2  3 | 0.0011  0.0019 | 0.0015 |
| 00000000323  (G1MQH0) | IPI00592879  F1NGN6; [8] | Uncharacterized protein/similar to ER mannosyl-oligosaccharide 1,2-α-mannosidase; domains: Glycoside hydrolase family 47 | A  B | | S,**I**  **S**,I | 11  14 | 0.0082  0.0147 | 0.0115 |
| 00000000324  (G1MQH1) | IPI00589043  F1NK40 | Uncharacterized protein; domains: α2-macroglobulin | A  B | | **S**,I  **S**,I | 5  15 | 0.0003  0.0011 | 0.0007 |
| 00000000330  (G1MQH6) | IPI00583396  O73840; [8] | Uncharacterized protein/similar to heparin cofactor II | A  B | | **S**,I  **S**,I | 5  8 | 0.0019  0.0041 | 0.0030 |
| 00000000333  (G1MQH8) | IPI00587189  Q6Q2J6 | Uncharacterized protein/similar to preprourotensin II | A  B | | S,**I**  **S**,I | 3  3 | 0.0214  0.0085 | 0.0140 |
| 00000000502  (G1MQW1) | IPI00821977  F1P0A9 | Uncharacterized protein; domains: Kunitz, amyloidogenic glycoprotein; shares 1 peptide with 0000015345 (G1NNT9) | A  B | | **S**,I  **S**,I | 10  8 | 0.0018  0.0066 | 0.0042 |
| 00000000549  (H9H062) | IPI00584049  ATPB | Mitochondrial ATP synthase subunit β | A  B | | I  **S**,I | 2  5 | 0.0002  0.0005 | 0.0004 |
| 00000000551  (G1MR04) | IPI00811898  F1N9C4 | Uncharacterized protein; domains: metallophosphoesterase | A  B | | **S**,I  **S**,I | 4  2 | 0.0033  0.0005 | 0.0019 |
| 00000000561  (G1MR11) | IPI00571559  H9KYX6; [17] | Uncharacterized protein/similar to selenium-binding protein 1 | A  B | | **-**  S | -  2 | -  0.0002 |  |
| 00000000565  (G1MR14)  00000018121  (G3UR23)  00000017514  (G3UPL6) | IPI00819939  F1NL36  IPI00818128  F1NL37  IPI00594466  F1NI70; [8] | Uncharacterized protein/similar to RET tyrosine-kinase RET receptor | A  B | | **S**,I  **S**,I | 7  9 | 0.0025  0.0049 | 0.0037 |
| 00000000586  (G1MR32) | IPI00600589  SPON1; [8] | Spondin-1 | A  B | | **S**,I  **S**,I | 8  13 | 0.0025  0.0097 | 0.0061 |
| 00000000610  (G1MR45)  00000017458  (G3UPG3) | IPI00586178  F1NU65  IPI00582195  F1NU67; [8] | Uncharacterized protein; domains: EGF, peptidase M12B, disintegrin, ADAM, signal seq | A  B | | S,I  **S**,I | 8  10 | 0.0017  0.0011 | 0.0014 |
| 00000000611  (G1MR46) | IPI00577489  F1NLW7; [8] | Uncharacterized protein; domains: SEA, CUB, trypsin, LDL receptor repeat | A  B | | **S**,I  **S**,I | 12  9 | 0.0058  0.0054 | 0.0056 |
| 00000000645  (G1MR66) | IPI00602790  F1NU46;[8] | Similar to stanniocalcin(-1) | A  B | | **S**,I  S | 5  5 | 0.0092  0.0053 | 0.0073 |
| 00000000679  (G1MR91) | IPI00577903  F1NUF8; [8] | α1-microglobulin/bikunin | A  B | | **S**,I  S | 3  3 | 0.0014  0.0011 | 0.0013 |
| 00000000704  (G1MRB1) | IPI00818415  F1NV93 | Uncharacterized protein/peptidyl-prolyl cis-trans isomerase (cyclophilins type) | A  B | | **S**,I  **S**,I | 5  5 | 0.0312  0.0877 | 0.0595 |
| 00000000718  (G1MRC3) | IPI00600838  A6N8N6; [8] | Uncharacterized protein/IGFBP 3 | A  B | | **S**,I  **S**,I | 4  5 | 0.0045  0.0106 | 0.0076 |
| 00000000764  (G1MRF6) | IPI00594508  E1BYC4 | Uncharacterized protein/similar to complement C2; domains:sushi/SCR/CCP, Peptidase S1/S6, VWA | A  B | | **S**,I  S,I | 8  8 | 0.0043  0.0027 | 0.0035 |
| 00000000781  (G1MRH0) | IPI00573900  IPI00578042  LOXL2; [8] | Lysyl oxidase homolog 2 | A  B | | S,**I**  S,**I** | 10  12 | 0.0051  0.0127 | 0.0089 |
| 00000000796  (G1MRI3) | IPI00575645  F1NWG7; [8] | Uncharacterized protein; domain: ARM_like; similar to ER chaperone/nucleotide exchange factor SIL1 | A  B | | I  S,**I** | 9  11 | 0.0019  0.0014 | 0.0015 |
| 00000000799  (G1MRI5)  00000017818  (G5E7I2) | IPI00589958  F1NDY9 | Uncharacterized protein; domain: protein disulfide isomerase | A  B | | S,**I**  S,**I** | 4  6 | 0.0010  0.0011 | 0.0011 |
| 00000000839  (G1MRL1)  00000017786  (G3UQB2) | IPI00571323  F1P4V1, FIBA | Fibrinogen α-chain | A  B | | I  - | 3  - | 0.0003  - |  |
| 00000000842  (G1MRL4) | IPI00582281, partial  F1NTQ2,partial; [8] | Uncharacterized protein/similar to β-hexosaminidase β-subunit | A  B | | S,**I**  S,**I** | 7  6 | 0.0286  0.0263 | 0.0275 |
| 00000000854  (G1MRM3) | IPI00594830  Q5ZLG8; [8] | Annexin A11 | A  B | | **S**,I  **S**,I | 10  7 | 0.0135  0.0071 | 0.0103 |
| 00000000859  (G1MRM7) | IPI00603596  F1NP60 | Uncharacterized protein; domains: FN2, ricin, CLECT, antifreeze, kringle_like | A  B | | S  S | 4  2 | <0.0001  <0.0001 | <0.0001 |
| 00000000865  (G1MRN3) | IPI00573193  H9KYW7; [8,17] | Uncharacterized protein/similar to hyaluronidase 1 | A  B | | **S**,I  **S**,I | 7  6 | 0.0066  0.0057 | 0.0062 |
| 00000000886  (G1MRP5) | IPI00592072  E1BV78, Q93568; [8] | Fibrinogen γ-chain | A  B | | S,**I**  S,I | 7  4 | 0.0014  0.0006 | 0.0010 |
| 00000000900  (G1MRQ6) | IPI00595826  GSLG1, F1P250; [8] | Golgi apparatus protein 1 | A  B | | **S**,I  **S**,I | 44  47 | 0.0066  0.2210 | 0.1138 |
| 00000000965  (G1MRV5) | IPI00586261  E1BTM1 | Eukaryotic peptide chain release factor subunit 1 | A  B | | -  S | -  3 | -  <0.0001 |  |
| 00000000966  (G1MRV6) | IPI00594898  F1NGY8 | Uncharacterized protein/similar to coagulation factor ; domains: GLA, peptidase S1/S6; shares 1 peptide with 00000013050 (G1NIF9) | A  B | | S  **S**,I | 5  7 | 0.0025  0.0023 | 0.0024 |
| 00000000969  (G1MRV8) | IPI00602928  Q5ZI46; [8,17] | Uncharacterized protein/similar to β-hexosaminidase α-subunit | A  B | | S,**I**  S,**I** | 8  10 | 0.0083  0.0170 | 0.0127 |
| 00000000970  (G1MRV9) | IPI00579663  H9L3E5 | ATP synthase α-subunit | A  B | | I  - | 3  - | 0.0001  - |  |
| 00000000984  (G1MRX0) | IPI00572548  CO1A1, P02453; [16] | Collagen α1 (I), fragment **^1^** | A  B | | S,I  - | 2  - | 0.0009  - |  |
| 00000001002  (G1MRY6) | IPI00586458  Q804X5 | Similar to anticoagulant protein C | A  B | | -  S,I | -  3 | -  0.0025 |  |
| 00000001025  (G1MS05) | IPI00603464  Q92170; [8] | Similar to histidine phosphatase of the ER, fragment | A  B | | S,I  **S**,I | 2  4 | 0.0028  0.0019 | 0.0024 |
| 00000001080  (H9H0A1)  00000019291  (H9H2I0) | IPI00812261  A2N884 | Similar to VH1 protein; shares peptides with other VH1 proteins | A  B | | S,**I**  - | 1 (3)  - | 0.0646  - |  |
| 00000001099  (G1MS56) | IPI00651180  Q5ZMK0; [8] | Uncharacterized protein/similar to cathepsin O | A  B | | S,I  **S**,I | 4  3 | 0.0018  0.0014 | 0.0016 |
| 00000001108  (H9H0A3)  00000001096  (H9H0A2) | IPI00585171  Q7T1N6, F1NFI6  [8] | Uncharacterized protein/similar to GDP-fucose protein O-fucosyltransferase 2 | A  B | | S,I  **S**,I | 10  7 | 0.0020  0.0015 | 0.0018 |
| 00000001119  (G1MS71) | IPI0000584176  E1C7S1; [8,17] | Uncharacterized protein/similar to neuronal pentraxin 2 | A  B | | S,**I**  **S**,I | 4  3 | 0.0024  0.0010 | 0.0017 |
| 00000001127  (G1MS78) | IPI00602423  PDGFC | PDGF-C | A  B | | S,**I**  I | 3  2 | 0.0008  0.0009 | 0.0009 |
| 00000001167  (G1MSA4) | IPI00574064  KPYK; [8] | Pyruvate kinase | A  B | | S,**I**  S | 4  7 | 0.0006  0.0018 | 0.0012 |
| 00000001173  (G1MSA8) | IPI00591151  F1P4X6; [8] | Uncharacterized protein/ITM (integral membrane protein) 2A; domain: BRICHOS | A  B | | S,**I**  **S**,I | 5  5 | 0.0045  0.0049 | 0.0047 |
| 00000001201  (G1MSC8) | IPI00588334  E1BXK1 | Uncharacterized protein; domains: LAMB IV, EGF | A  B | | S,I  S,**I** | 6  13 | 0.0006  0.0010 | 0.0008 |
| 00000001205  (G1MSD1) | IPI00603434  E1C4X0; [8] | Uncharacterized protein/similar to dentin matrix protein 4/FAM20C | A  B | | **S**,I  **S**,I | 12  10 | 0.2420  0.2947 | 0.2684 |
| 00000001272  (G1MSI1) | IPI00586821  CO6A2; [8] | Collagen α2 (VI) **^1^** | A  B | | S  **S**,I | 4  9 | 0.0001  0.0009 | 0.0005 |
| 00000001277  (H9H0B3)  00000017722  (H9H285) | - | Similar to annexin (A4), fragments | A  B | | S,I  **S**,I | 8  6 | 0.0026  0.0009 | 0.0018 |
| 00000001294  (G1MSJ8) | IPI00822825  F1NI05, F1NK49; [8] | Uncharacterized protein/similar to laminin subunit γ1 | A  B | | S,I  S,I | 19  21 | 0.0066  0.0086 | 0.0076 |
| 00000001387  (G1SMR7) | IPI00603337  F1NY48 | Uncharacterized protein/similar to relaxin receptor 1/similar to leucine-rich repeat-containing G-protein-coupled receptor 7 | A  B | | -  S | -  2 | -  0.0003 |  |
| 00000001450  (G1MSW3) | IPI00603309  HSP7C; [8] | Heat shock cognate 71kDa protein F1NWP3_CHICK | A  B | | S,I  **S**,I | 11 (13)  10 (20) | 0.0066  0.0151 | 0.0109 |
| 00000001453  (G1MSW6) | IPI00576631  F1NIZ7 | Uncharacterized protein; domains: SEA | A  B | | **-**  S | -  2 | -  0.0002 |  |
| 00000001458  (G1MSX1) | IPI00603974  E1C6M8; [8] | Similar to EGF-containing fibulin-like extracellular matrix protein 1 | A  B | | **S**,I  **S**,I | 9 (10)  10 | 0.0202  0.0191 | 0.0197 |
| 00000001491  (G1MSZ9; Q8AY76) | IPI00601265  AVID; [8] | Avidin | A  B | | **S**,I  **S**,I | 13  11 | 2.6822  2.6512 | 2.6667 |
| 00000001515  (G1MT18)  00000001510  (G1MT14) | IPI00574962  F1P144  IPI00819257  E1BVD3 | Uncharacterized protein/similar to leucine zipper protein 2 | A  B | | **S**,I  - | 2  - | 0.0004  - |  |
| 00000001513  (G1MT16)  00000013789  (G1NK65) | IPI00579747  FGFR3  IPI00598143  F1P3S0 | Fibroblast growth factor receptor | A  B | | S  **S**,I | 5  6 | 0.0020  0.0021 | 0.0021 |
| 00000001542  (G1MT40) | IPI00587849  F1NLP7 [8] | Similar to antithrombin | A  B | | **S**,I  **S**,I | 24  27 | 0.1080  0.1250 | 0.1165 |
| 00000001556  (G1MT53) | IPI00598170  E1C1N1 | Uncharacterizede protein/similar to plakophilin-1 | A  B | | -  S | -  2 | -  <0.0001 |  |
| 00000001557  (G1MT54) | IPI00579636  F1NW32, NEO1; [8] | Neogenin-1 | A  B | | S  S | 5  2 | 0.0003  0.0002 | 0.0003 |
| 00000001575  (G1MT68) | IPI00604297  F1NYZ7; [8] | Similar to CLIC4 protein (chloride intracellular channel 4) | A  B | | S,**I**  **S**,I | 3  3 | 0.0010  0.0012 | 0.0011 |
| 00000001580  (G1MT72) | IPI00587416  F1NF15; [8] | Similar to dynein light chain roadblock type 1 | A  B | | **-**  S | -  2 | -  0.0007 |  |
| 00000001590  (G1MT79) | IPI00584751  F1P4K2 [8] | Sortin-related receptor/sortilin-1 | A  B | | **S**,I  **S**,I | 19  22 | 0.0006  0.0010 | 0.0008 |
| 00000017375  (G3UP83)  00000001643  (G1MTC0) | IPI00596507  O93419; [8] | Collagen XVIII **^1^** | A  B | | **-**  **S**,I | -  4 | -  0.0005 |  |
| 00000001721  (G1MTI5) | IPI00574082  E1C7K2; [8] | Uncharacterized protein/similar to matrix metalloproteases 17 | A  B | | **-**  S | -  2 | -  0.0002 |  |
| 00000001753  (G1MTK9)  00000009077  (G1I9N6)  00000008592  (G1N8F8)  00000018592  (G3QSU3)  00000018813  (G1MYE7)  00000003967  (G3USX9)  00000019643  (G3X8P3) | IPI00570724  TBB3  IPI00592950  TBB1  IPI00591483  TBB7  IPI00597391  TBB2  [8,17] | β-tubulins | A  B | | S,**I**  S,**I** | 17  18 | 0.0480  0.0165 | 0.0323 |
| 00000001774  (G1MZM6) | IPI00602683  E1C9J8 | Similar to Arg-rich mutated in early stage tumours/similar to mesencephalic astrocyte-derived neurotrophic factor | A  B | | S  S | 5  5 | 0.0030  0.0054 | 0.0042 |
| 00000001821  (G1MTR1)  00000010409  (G1NCE5) | IPI00580429  IPI00576313  F4MAY7 [8] | Plakoglobin; fragments | A  B | | S,**I**  **S**,I | 5  4 | 0.0302  0.0316 | 0.0309 |
| 00000001835  (G1MTS2)  00000019323  (G3UU71) | IPI00578325  F1NIZ9; [8] | GTP-binding protein/rho GTPase | A  B | | S,I  **S**,I | 2  4 | 0.0015  0.0025 | 0.0020 |
| 00000001848  (G1MTT4) | IPI00582263  RAN | GTP-binding nuclear protein Ran | A  B | | **-**  **S**,I | -  4 | -  0.0009 |  |
| 00000002344  (G1MUU5)  00000001887  (G1MTV9) | IPI00590538  Q5ZJH4; [8] | Uncharacterized protein/ADAM9; domains: EGF, ADAM7_reprolysin, disintegrin | A  B | | **S**,I  **S**,I | 2  2 | 0.0004  0.0009 | 0.0007 |
| 00000001902  (G1MTX0) | IPI0000603114  OAF; [8] | Out at first protein homolog | A  B | | S,I  **S**,I | 4  4 | 0.0021  0.0028 | 0.0025 |
| 00000001910  (G1MTX5) | IPI00594349  Q5ZKV5; [8] | Uncharacterized protein; domains: arrestin | A  B | | S,I  **S**,I | 6  6 | 0.0325  0.0316 | 0.0321 |
| 00000001935  (G1MTZ5) | IPI00588327  F1P397 | Similar to testican-3 | A  B | | S  **S**,I | 9  6 | 0.0030  0.0044 | 0.0037 |
| 00000001937  (G1MTZ6) | IPI00680904  E1C015 | Uncharacterized protein/similar to poliovirus-related protein-1; domains: IG | A  B | | S  S | 4  4 | 0.0041  0.0035 | 0.0038 |
| 00000001973  (G1MU19) | IPI00582446  LIPL | Lipoprotein lipase | A  B | | S,I  I | 2  2 | 0.0004  0.0004 | 0.0004 |
| 00000002025  (G1MU55) | IPI00584710  F1NWF7; [8] | Similar to arylsulfatase A | A  B | | **S,**I  S,**I** | 2  3 | 0.0020  0.0027 | 0.0024 |
| 00000002065  (G1MU86)  00000010131  (G1NBT6) | IPI00596111  Q5ZL15; [8] | Similar to G protein β4 | A  B | | S,I  - | 2  - | 0.0014  - |  |
| 00000002079  (H9H0E9) | IPI00580985  Q6EE32; [8] | Calreticulin, fragment | A  B | | S,**I**  **S**,I | 6  7 | 0.0026  0.0036 | 0.0031 |
| 00000002157  (G1MUF5)  00000009298  (H9H1I8) | IPI00823231  E1BTS8  IPI00586802  H9KYT1 | Uncharacterized protein/similar to transitional ER ATPase; domains: AAA+ ATPase, CDC48, vps4_C | A  B | | -  S | -  6 (8) | -  0.0005 |  |
| 00000002159  (G1MUF6) | IPI00579332  E1BTE7 | Uncharacterized protein/similar to torsin 3A;domains: ClpA/B, AAA+ ATPase, torsin, signal seq | A  B | | S,**I**  S,**I** | 3  5 | 0.0007  0.0009 | 0.0008 |
| 00000002213  (G1MUJ6)  00000015905  (G1NQ43) | IPI00574482  Q5ZI39  IPI00588878  Q5ZLW0 | Similar to plastin 2/3 | A  B | | -  S | -  8 (10) | -  0.0009 |  |
| 00000002217  (G1MUJ9) | IPI01017338  F1P4U3 | Secreted phosphoprotein 24 | A  B | | -  S | -  3 | -  0.0005 |  |
| 00000002239  (G1MUL7)  00000006481  (G1N3Z1) | IPI00592851  Q5ZMA0; [8]  IPI00592098  Q5ZKR9; [8] | ADP-ribosylation factor 1/4 | A  B | | S,I  **S**,I | 6  6 | 0.0023  0.0033 | 0.0028 |
| 00000002256  (G1MUM9) | IPI00592986  F1NCV8; [8] | Similar to ER lectin 1 | A  B | | S,**I**  S,I | 3  3 | 0.0016  0.0009 | 0.0013 |
| 00000002265  (G1MUN6) | IPI00579591  Q90XC6; [8] | Prominin-like protein; domain: prominin | A  B | | **S**,I  S | 4  2 | 0.0019  0.0017 | 0.0018 |
| 00000002303  (G1MUR4) | IPI00596570  E1BU50; [8,9] | Uncharacterized protein/similar to Golgi phosphoprotein 4 (GPP130) | A  B | | **S**,I  **S**,I | 2  3 | 0.0052  0.0092 | 0.0072 |
| 00000002353  (G1MUV3) | IPI00595452  Q5ZI96; [8] | Similar to glycosaminoglycan xylosekinase | A  B | | S,I  S,**I** | 5  4 | 0.0015  0.0030 | 0.0026 |
| 00000002380  (G1MUX4) | IPI00821924  F1P456 | Similar to Npepps/puromycin-sensitive aminopeptidase | A  B | | -  **S**,I | -  4 | -  0.0002 |  |
| 00000002383  (G1MUX6) | IPI00590509  F1NPH3; [8] | Uncharacterized protein; domains:VWA, FN3, signal seq | A  B | | S,**I**  S,I | 9  8 | 0.0691  0.0434 | 0.0563 |
| 00000002465  (G1MV34) | IPI00582790  IPI00596872  F1NNM9, [8] | Uncharacterized protein; domains: semaphorin, IG, plexin (semaphorin 3D) | A  B | | S,**I**  S,**I** | 16  15 | 0.0787  0.0628 | 0.0708 |
| 00000002510  (G1MV71) | IPI00602837  - | Uncharacterized protein; domains: Leucine-rich repeat | A  B | | S,I  **S**,I | 2  2 | 0.0007  0.0012 | 0.0010 |
| 00000002528  (G1MV84)  00000019210  (G3UTX4) | IPI00571581  QSOX1; [8,16,17] | Sulfhydryl oxidase 1 | A  B | | S,I  S.I | 27  23 | 1.1842  1.5773 | 1.0838 |
| 00000002606  (G1MVE1) | IPI00601326  E1BQ84 | Similar to copine-2; shares peptides with 00000019594 (G3UUX4) | A  B | | S,**I**  - | 2 (3)  - | 0.0002  - |  |
| 00000002667  (G1MVJ0) | IPI00590771  F1P3U8 | Uncharacterized protein/similar to fibulin-7; domains: Sushi/SCR/CCP, EGF | A  B | | S,I  - | 2  - | 0.0002  - |  |
| 00000002668  (G1MVJ1) | - | Uncharacterized protein/ICAM4; domains: intercellular adhesion molecule (ICAM/VCAM) | A  B | | S  S | 2  2 | 0.0007  0.0002 | 0.0005 |
| 00000002678  (Q4VTT6) | IPI00575264  ZP3 | Zona pellucid protein C (ZPC) | A  B | | S,**I**  - | 7  - | 0.0076  - |  |
| 00000018684  (G3USK9)  00000002691  (G1MVK8) | IPI00575769  CO6A3 | Collagen α3 (VI) **^1^** | A  B | | S,**I**  S,**I** | 16  29 | 0.0003  0.0010 | 0.0007 |
| 00000002751  (G1QMV6) | IPI00602488  E1BXJ0; [8] | Protein-L-isoaspartate O-methyltransferase | A  B | | S,**I**  **S**,I | 6  4 | 0.0013  0.0024 | 0.0019 |
| 00000002758  (G1MVR1)  00000002763  (G1MVR6) | IPI00584718  Q90824  IPI01017282  TENA, [8] | Tenascin/cytotactin (largely overlapping peptide sets) | A  B | | S,**I**  S,**I** | 40  36 | 0.0161  0.0182 | 0.0172 |
| 00000002760  (G1MVR3) | IPI00597243  F1NDN9; [8] | Keratin 19; shares peptides with common contaminants | A  B | | S,**I**  S,**I** | 5 (6)  3 (4) | 0.0062  0.0019 | 0.0041 |
| 00000002805  (G1MVV5)  00000002725  (G1MVN4) | IPI00578012  E1BQC2  IPI00683271  TRFE, [8,12,16,17] | Ovotransferrin (+BB, CC, BC) | A  B | | **S**,I  **S**,I | 75  71 | 2.3526  2.0783 | 2.2155 |
| 00000002809  (G1MVV8) | IPI00579242  O93601; [8,17] | Apolipoprotein A-IV | A  B | | S,I  **S**,I | 20  14 | 0.0073  0.0054 | 0.0064 |
| 00000002825  (G1MVW7) | IPI00590797 [8] | Uncharacterized protein, fragment; similar to transmembrane protein 132A/similar to GRP78-binding protein | A  B | | **S**,I  **S**,I | 3  4 | 0.0010  0.0015 | 0.0013 |
| 00000002830  (G1MVX1) | IPI00580765  APOA1; [8] | Apolipoprotein A-I | A  B | | **S**,I  **S**,I | 16  15 | 0.0576  0.0338 | 0.0457 |
| 00000002860  (G1MVZ0) | IPI00577696  F1NAU1; [8] | Uncharacterized protein/similar to glypican-4 | A  B | | **S**,I  **S**,I | 19  18 | 0.1189  0.1169 | 0.1179 |
| 00000002869  (G1MVZ6) | IPI00593849  F1NFM9 | Uncharacterized protein; similar to FAM132A; domains: C1q/TNF_like, signal seq | A  B | | S,I  S,**I** | 3  3 | 0.0019  0.0020 | 0.0020 |
| 00000002904  (G1MW20) | IPI00589269  CAB45; [8,16] | 45kDa calcium-binding protein | A  B | | **S**,I  **S**,I | 14  14 | 0.0893  0.0855 | 0.0874 |
| 00000002906  (G1MW21) | IPI00577879  A4VAR9; [8] | Dystroglycan | A  B | | **S**,I  **S**,I | 17  21 | 0.0264  0.0335 | 0.0300 |
| 00000002923  (G1MW36) | IPI00584718  [8] | Uncharacterized protein; domains: cadherin | A  B | | **S**,I  **S**,I | 8  8 | 0.0050  0.0038 | 0.0044 |
| 00000002948  (G1MW54) | IPI00578517  Q9DER4 | Zona pellucida protein (ZP) -1 | A  B | | S,**I**  - | 6  - | 0.0022  - |  |
| 00000002964  (G1MW67) | IPI00811337  F1NBV6, H9L1M8 | Similar to sia-α-2,3-Gal-β-1,4-GlcNAc-R:α-2,8-sialyltransferase/α-2,8-sialyltransferase; | A  B | | S,I  **S**,I | 5  6 | 0.0025  0.0060 | 0.0043 |
| 00000003022  (G1MWB3) | IPI00585165  E1BUG8; [8] | Uncharacterized protein/similar to galactose-3-O-sulfotransferase 2; shares 1 peptide with 00000015265 (G1NNM9) | A  B | | S,**I**  S,I | 15 (16)  15 (16) | 0.0211  0.0194 | 0.0203 |
| 00000003066  (G1MWE3) | IPI00600808  RAB5C, [8] | Ras-related Rab 5A/5B/5C; overlaping peptide sets | A  B | | -  S,I | -  3 | -  0.0010 |  |
| 00000003069  (G1MWE6) | IPI00588491  E1C5U6; [8] | Uncharacterized protein/similar to villin-1; domains: villin, gelsolin | A  B | | -  **S**,I | -  6 | -  0.0006 |  |
| 00000003081  (G1MWF4) | IPI00589584  O93382; [8] | Rab-GDP dissociation inhibitor | A  B | | S,I  **S**,I | 8  8 | 0.0007  0.0014 | 0.0011 |
| 00000003137  (G1MWJ4) | IPI00590535  F1NJT3; [8,9,16] | Fibronectin, fragment | A  B | | S,**I**  S,**I** | 38  33 | 0.1571  0.1064 | 0.1318 |
| 00000017932  (G3UQJ3)  00000003150  (G1MWK4)  00000006080  (G1N321)  00000019446  (G3UUI2) | IPI00602984  AGRIN, P31696-2 to 6; [8] | Agrin, fragments with largely overlapping peptide sets | A  B | | S,**I**  S,**I** | 44  42 | 0.0445  0.0200 | 0.0323 |
| 00000003153  (G1MWK6)  00000003587  (G1MXK7) | IPI00571356  E1BQ45 | Similar to matrilin-4; overlapping fragments | A  B | | S,**I**  S,**I** | 21  22 | 0.0800  0.0785 | 0.0793 |
| 00000003213  (G1MWQ7) | IPI00597105  F7NIZ8; [8] | Uncharacterized protein/similar to meteorin-like protein | A  B | | **S**,I  **S**,I | 11  10 | 0.0162  0.0136 | 0.0149 |
| 00000003254  (G1MWU3) | IPI00598113  Q8JIG5; [8] | Uncharacterized protein/ovoglycoprotein/ α1-acid glycoprotein; domain: α1-acid glycoprotein/calycin/apolipoprotein M, signal | A  B | | **S**,I  **S**,I | 3  4 | 0.0307  0.0705 | 0.0506 |
| 00000003289  (G1MWX1) | IPI00584442  E1C8J7 | Similar to thioredoxin-related transmembrane protein 2 | A  B | | **-**  **S**,I | -  2 | -  0.0003 |  |
| 00000003326  (G1MWZ9) | IPI00819959  E1BXV7  IPI00591736  E1BRS7 | Uncharacterized protein; domains: ANATO, α2-macroglobulin, netrin module, signal seq | A  B | | **S**,I  S,I | 6  10 | 0.0003  0.0004 | 0.0004 |
| 00000003352  (G1MX21) | IPI00584715  CP080 | UPF0468 protein C16orf80 homolog | A  B | | S,I  - | 2  - | 0.0003  - |  |
| 00000017223  (G3UNU3)  00000003363  (Q5I5K4) | IPI00584027  SDC4 | Syndecan-4 | A  B | | S  S | 3  2 | 0.0118  0.0123 | 0.0121 |
| 00000003416  (G1MX70) | IPI00590750  F1P3M4 | Collagen, similar to α1 type VII or α1 type V **^1^** | A  B | | S,I  **S**,I | 17  20 | 0.0011  0.0022 | 0.0017 |
| 00000003451  (G1MX98) | IPI00575584  ENOA, F1NZ78; [8] | α-enolase; shares several peptides with 00000014948 (G1NMWo; γ-enolase) | A  B | | S,I  **S**,I | 7 (11)  13 | 0.0030  0.0076 | 0.0053 |
| 00000003476  (G1MXB6) | IPI00577857  E1BVT3; [8] | Malate dehydrogenase | A  B | | **-**  **S**,I | -  4 | -  0.0004 |  |
| 00000003479  (G1MXB9) | - | Similar to complement factor D | A  B | | **S**,I  S | 3  3 | 0.0015  0.0010 | 0.0013 |
| 00000003490  (G1MXC7) | IPI00602403  RABP1 | Cellular retinoic acid-binding protein 1 | A  B | | S,I  S | 3  2 | 0.0096  0.0080 | 0.0088 |
| 00000003498  (G1MXD5) | IPI00602177  E1C8L4; [8] | Uncharacterized protein/similar to transmembrane protease serine 13 | A  B | | **S**,I  **S**,I | 5  5 | 0.0037  0.0051 | 0.0044 |
| 00000003507  (G1MXE4) | IPI00597742  E1C3F5 | Uncharacterized protein/similar to torsin-2A | A  B | | I  S,I | 3  3 | 0.0004  0.0005 | 0.0005 |
| 00000003543  (G1MXH6) | IPI00602986  E1BWI0; [8] | Similar to desmoplakin | A  B | | **S**,I  **S**,I | 10  14 (15) | 0.0018  0.0022 | 0.0020 |
| 00000003557  (G1MXI6) | IPI00585935  F1NQ93; [8,9,16] | Uncharacterized protein/similar to semaphorin-3G | A  B | | S,**I**  S,**I** | 34  31 | 0.1470  0.1611 | 0.1541 |
| 00000003608  (G1MXM3) | IPI00603117  F1NKZ7; [8] | Uncharacterized protein/similar to PKHD domain-containing transmembrane protein C17orf101 homolog; domains: prolyl 4-hydroxylase α-subunit | A  B | | S,**I**  - | 3  - | 0.0007  - |  |
| 00000003666  (G1MXR5) | IPI00598002  F1N9M8; [8] | Uncharacterized protein/similar to transmembrane protease serin 4 | A  B | | S,**I**  S,I | 4  3 | 0.0011  0.0007 | 0.0009 |
| 00000003671  (G1MXR9) | IPI00590040  LY86; [8] | Lymphocyte antigen 86 | A  B | | S  - | 2  - | 0.0017  - |  |
| 00000017906  (G5E7N2)  00000003673  (G1MXS0) | IPI00590524  F1NLV8, PTK7 | Interactive tyrosine kinase 7 | A  B | | S  S | 5  3 | 0.0001  <0.0001 | <0.0001 |
| 00000003675  (G1MXS1) | IPI00598167  E1C0B0; [8] | Uncharacterized protein/similar to group XV phospholipase A2 | A  B | | **-**  S | -  4 | -  0.0009 |  |
| 00000003721  (G1MXV5) | IPI00591690  Q9DDD3 | Calsyntenin-1; domains: cadherin | A  B | | **S**,I  **S**,I | 12  13 | 0.0040  0.0048 | 0.0044 |
| 00000003758  (G1MXY2) | IPI00591852  1433B | 14-3-3 protein α/β; shares 1 peptide with other 14-3-3 proteins | A  B | | S,**I**  **S**,I | 4  5 | 0.0014  0.0013 | 0.0014 |
| 00000003844  (G1MY48) | IPI00587482  E1C7M8 | Uncharacterized protein/similar to dyslexia-associated protein KIAA0319-like | A  B | | **S**,I  **S**,I | 2  2 | 0.0016  0.0018 | 0.0017 |
| 00000003854  (G1MY55)  00000017647  (G3UPY6) | IPI00574152  E1C6P3 | P2X purinoreceptor | A  B | | -  **S**,I | -  3 | -  0.0002 |  |
| 00000003857  (G1MY58) | IPI00586605  Q6QWE7, F1NRD4 | Similar to adiponectin | A  B | | S,I  S | 3  2 | 0.0012  0.0002 | 0.0007 |
| 00000003874  (G1MY70) | IPI00819343  F1NS88 | Similar to isochorismate domain-containing protein 1 | A  B | | I  - | 2  - | 0.0003  - |  |
| 00000003916  (G1MYA6) | - | Similar to lectin mannose-binding 2 variant 1 | A  B | | **S**,I  - | 3  - | 0.0020  - |  |
| 00000003921  (G1MYB0) | IPI00587947  F1NF50 | α-N-acetylgalactosaminide α-2,3-sialyltransferase 2 | A  B | | S,I  **S**,I | 6  8 | 0.0064  0.0133 | 0.0099 |
| 00000003937  (G1MYC4) | IPI00586834  F1NU56 | Similar to ribosomal protein S25 | A  B | | **-**  S | -  3 | -  0.0007 |  |
| 00000003945  (G1MYD1) | IPI00575580  Q4AEI5, partial; [8] | Similar to complement regulatory GPI-anchor protein CREG | A  B | | **S**,I  **S**,I | 3  3 (4) | 0.0006  0.0008 | 0.0007 |
| 00000003950  (G1MYD5) | IPI00572510  Q4AEJ1; [8] | Similar to complement regulatory soluble protein CRES; aa34-228 100% identical to aa40-235 of G1MYD1 | A  B | | -  **S**,I | -  2 | -  0.0005 |  |
| 00000003953  (G1MYD7) | -  E1C080 | Similar to Ras-related Rab-8B; shares 1 peptide with 00000011581 (G1NF26; Rab-3B) | A  B | | I  - | 1 (2)  - | 0.0003  - |  |
| 00000003963  (G1MYE5) | IPI00602001  Q9DEG0, F1P3M9 | Uncharacterized protein/complement regulatory membrane protein, fragment | A  B | | S  - | 2  - | 0.0007  - |  |
| 00000004000  (G1MYH2) | IPI00600529  F2Z4K7 | Ribosomal protein S3A | A  B | | -  S | -  3 | -  0.0001 |  |
| 00000004026  (G1MYJ4) | IPI00591895  Q8QGU9 | Semaphorin-3F | A  B | | S,I  S,I | 4  4 | 0.0003  0.0004 | 0.0004 |
| 00000004038  (G1MYK6,  OVAL)  00000004045  (G1MYL1) | IPI00583974  OVAL; [10,8,9,16,17] | Ovalbumin; N-term: acetyl-Gly_2_ | A  B | | S,**I**  S,**I** | 28  22 | 11.8539  13.1873 | 12.5206 |
| 00000004064  (G1MYM6) | IPI00583624  E1C857; [8] | Uncharacterized protein/similar to tetraspanin 6 | A  B | | S,I  **S**,I | 2  2 | 0.0019  0.0016 | 0.0018 |
| 00000004077  (G1MYN4) | IPI00577674  P61355 | Ribosomal protein L27 | A  B | | **-**  S | -  2 | -  0.0010 |  |
| 00000004106  (G1MYQ7) | IPI00601812  SFRP3 | Secreted frizzled-related protein 3 | A  B | | **S**,I  S | 2  3 | 0.0052  0.0162 | 0.0107 |
| 00000004109  (G1MYQ9) | IPI00583304  E1BTM0; [8] | Uncharacterized protein/similar to inter-α-trypsin inhibitor H5 | A  B | | S,I  S,I | 27  26 | 0.0338  0.0302 | 0.025 |
| 00000004117  (G1MYR4) | H9L296 | Uncharacterized protein/similar to semaphorin-3B | A  B | | S,**I**  S,**I** | 6  7 | 0.0041  0.0031 | 0.0036 |
| 00000004128  (G1MYS3) | IPI00572003  Q5ZLB6, F1P1L8 | Uncharacterized protein/similar to serpin peptidase inhibitor clade B member 6 | A  B | | -  S | -  2 | -  0.0001 |  |
| 00000004181  (G1MYW7) | IPI00597333  F1NUG0 | Neuroendocrine convertase 2; domains: Peptidase S8/S53, preprotein convertase P | A  B | | I  **S**,I | 4  5 | 0.0025  0.0042 | 0.0034 |
| 00000004191  (G1MYX6) | IPI00577013  PRDX6; [8] | Peroxiredixin-6 | A  B | | **S**,I  **S**,I | 9  7 | 0.0043  0.0099 | 0.0071 |
| 00000004197  (G1MYY0) | IPI00577279  B3VE14 | Inter-α-inhibitor H2 | A  B | | S  S | 5  6 | 0.0003  0.0005 | 0.0004 |
| 00000004203  (G1MYY5) | IPI00680755  F1NLB0 | NCAM1 | A  B | | -  S | -  2 | -  <0.0001 |  |
| 00000004242  (G1MZ16)  00000014144  (G1NKZ8) | IPI00600819  COAA1, F1NRH2  [8] | Collagen α1 (X) **^1^** | A  B | | S,**I**  S,I | 9  8 | 0.0378  0.0931 | 0.0655 |
| 00000004255  (G1MZ27) | IPI00570964  Q5ZIZ0 | 6-phosphogluconate dehydrogenase, decarboxylating | A  B | | -  S,I | -  3 | -  0.0009 |  |
| 00000004285  (G1MZ49) | IPI00584733  MIME | Mimecan | A  B | | S,**I**  S,**I** | 10  8 | 0.0115  0.0312 | 0.0214 |
| 00000004287  (G1MZ51) | IPI00587181  F1NI86 | Uncharacterized protein/similar to β-glucuronidase | A  B | | S,**I**  S,**I** | 4  6 | 0.0010  0.0029 | 0.0020 |
| 00000004290  (G1MZ54)  00000019160  (G3UTS6) | IPI00585699  HYOU1; [8] | Hypoxia-upregulated protein 1; domains: HSP70 | A  B | | S,I  **S**,I | 11  13 | 0.0014  0.0028 | 0.0021 |
| 00000004389  (G1MZD0) | IPI00584175  FPPS | Farnesyl pyrophosphate synthase | A  B | | I  S,**I** | 4  2 | 0.0002  0.0001 | 0.0002 |
| 00000004400  (G1MZD9) | IPI00577039  ANXA2; [8] | Annexin A2; N-term: acetyl-Ser_2_ | A  B | | **S**,I  **S**,I | 20  20 | 0.0372  0.0330 | 0.0351 |
| 00000004408  (G1MZE6) | IPI00570865  E1BZV4; [8] | Uncharacterized protein/similar to REG4; domains: CLECT, signal seq | A  B | | **S**,I  **S**,I | 9  8 | 0.4324  0.3306 | 0.3815 |
| 00000004414  (G1MZF2)  00000019771  (G3UV97) | IPI00585509  ARP2 | Actin-related protein 2 | A  B | | -  S,I | -  2 | -  0.0005 |  |
| 00000004416  (G1MZF4) | IPI00573738  OVALY [8,16,17] | Ovalbumin-related Y; shares 1 peptide with ovalbumin-related X | A  B | | S,I  **S**,I | 12 (13)  15 (16) | 0.2839  0.5684 | 0.4262 |
| 00000004436  (G1MZH2) | IPI00580221  OVALX [8,17] | Ovalbumin-related X; shares 1 peptide with ovalbumin-related Y | A  B | | S,I  S,I | 3  4 | 0.0048  0.0049 | 0.0049 |
| 00000004444  (G1MZI0) | IPI00571119  E1BZH8 [8] | Similar to heparan sulfate 6-O endosulfatase 2 | A  B | | **S**,I  S | 2  4 | 0.0093  0.0108 | 0.0101 |
| 00000004472  (G1MZK2) | IPI00684373  F1NNL7 | Similar to RAB-1B | A  B | | S,I  **S**,I | 2  2 | 0.0020  0.0029 | 0.0025 |
| 00000004474  (G1MZK4) | IPI00583999  F1NLE0 | Uncharacterized protein/similar to cadherin-related family member 2; domains: cadherin, signal seq | A  B | | **S**,I  **S**,I | 9  13 | 0.0043  0.0057 | 0.0050 |
| 00000004530  (G1MZP2) | IPI00575487  F1NJ71; [8] | Similar to plasma membrane calcium-transporting ATPase 2 | A  B | | S,I  **S**,I | 4  8 | 0.0005  0.0008 | 0.0007 |
| 00000010136  (G1NBU1)  00000004544  (H9H0Q7) | IPI00573563  Q5F3R8, RB11A  [8] | Ras-related Rab-11A | A  B | | -  **S**,I | -  2 | -  0.0016 |  |
| 00000004553  (G1MZQ7)  0000019713  (G3UV44) | IPI00592257  F1P5W0 (partial) | Similar to FRAS1-related extracellular matrix protein 1 (FREM1) | A  B | | S,**I**  S,**I** | 19  19 | 0.0080  0.0103 | 0.0092 |
| 00000004590  (G1MZT6) | IPI00579092  F1P2P9; [8] | 14-3-3 protein ε; shares 1 of 5 peptides with other 14-3-3 proteins | A  B | | S,**I**  **S**,I | 4  3 | 0.0016  0.0014 | 0.0015 |
| 00000012434  (G1NH13)  etc | IPI00684128  G1K239 | Histone H3 | A  B | | S,I  - | 2  - | 0.0092  - |  |
| 00000004613  (G1MZV1) | IPI00584371  SAP; [8,17] | Proactivator polypeptide (prosaposin) | A  B | | **S**,I  **S**,I | 14  16 | 0.0485  0.0707 | 0.0596 |
| 00000004627  (H9H0Q9) | IPI00587828  A2N887; [8] | Similar to VH1 protein; shares peptides with other VH1 proteins | A  B | | **-**  S,I | -  1 (4) | -  0.0911 |  |
| 00000004638  (G1MZX1) | IPI00587313  IOV7; [8,17] | Ovoinhibitor; shares 1 peptide with 00000016390 (G1NR45) | A  B | | **S**,I  **S**,I | 9 (10)  9 (10) | 0.0096  0.0062 | 0.0079 |
| 00000004655  (G1MZY1;  IOVO)  00000019272  (G3UU27) | IPI00597129  IOVO; [8] | Ovomucoid | A  B | | **S**,I  **S**,I | 10  11 | 0.0606  0.0409 | 0.0508 |
| 00000004657  (G1MZY3) | IPI00684905  E1BRK7 | Uncharacterized protein/similar to angiopoietin-related protein 7 (ANGL7) | A  B | | I  S,I | 5  2 | 0.0015  0.0004 | 0.0010 |
| 00000004724  (G1N036) | IPI00812899  F1NAH7 | Uncharacterized protein/similar to somatomedin B- and thrombospondin type 1-containing protein; domain: thrombospondin type 1 | A  B | | **S**,I  S | 4  3 | 0.0024  0.0034 | 0.0029 |
| 00000004753  (H9H0S3) | IPI00572547  S10A6; [8] | S100-A6/calcyclin | A  B | | S  S | 2  2 | 0.0050  0.0021 | 0.0036 |
| 00000004774  (G1N071) | IPI00583336  MDHC; [8] | Malate dehydrogenase, fragment (aa1-334) | A  B | | S,I  **S**,I | 5  5 | 0.0022  0.0038 | 0.0030 |
| 00000004837  (G1N0B7) | IPI00588459  F1NIB5 | Similar to TIMP-4 | A  B | | S  S | 3  3 | 0.0010  0.0007 | 0.0009 |
| 00000004906  (G1N0G6) | IPI00577008  MYO1C; [8] | Unconventional myosin-Ic | A  B | | -  S,I | -  3 | -  <0.0001 |  |
| 00000004966  (G1N0L0) | IPI0000576840  F1NWW1; [8,17] | Uncharacterized protein/similar to torsin-1B | A  B | | S,**I**  S,I | 12  10 | 0.0124  0.0085 | 0.0105 |
| 00000004982  (G1N0M3) | IPI00576825  Q5ZL76 | Uncharacterized protein/similar to T-cell immunomodulatory protein (TIP) | A  B | | **S**,I  **S**,I | 6  7 | 0.0051  0.0054 | 0.0053 |
| 00000004984  (G1N0M4) | IPI00587134  F1NVQ5; [17] | Uncharacterized protein/similar to thrombospondin type-1 domain-containing protein 4 | A  B | | S,**I**  S,**I** | 5  5 | 0.0006  0.0017 | 0.0012 |
| 00000004995  (G1N0N0) | IPI00603341  F1P2B1, Q5ZIP1 | Uncharacterized protein/similar to torsin-1A | A  B | | I  - | 2  - | 0.0001  - |  |
| 00000005097  (G1N0W4) | IPI00589818  EPCAM; [17] | Epithelial cell adhesion molecule | A  B | | S,I  **S**,I | 2  2 | 0.0004  0.0002 | 0.0003 |
| 00000005099  (G1N0W6)  00000017866  (G5E7K5)  00000518893  (G3UT58) | IPI00589264  F1NI72, CO3A1  [16] | Collagen α1 (III), fragment **^1^** | A  B | | **-**  S | -  2 | -<0.0001 |  |
| 00000005136  (G1N0Z7) | IPI00680258  F1NAR5; [16,17] | Uncharacterized protein/serpinF2/similar to α2-antiplasmin | A  B | | **S**,I  **S**,I | 17  14 | 0.8439  0.7500 | 0.7970 |
| 00000005175  (G1N131) | IPI00587267  E1C7H6; [8,9,17] | Uncharacterized protein/serpinF1/PEDF/α2-antiplasmin | A  B | | S,I  S,I | 26  24 (25) | 0.7510  0.5270 | 0.639 |
| 00000005200  (G1N152) | IPI00585193  F1NBY4; [8] | Similar to Notum pectin acetylesterase-like protein | A  B | | S,I  **S**,I | 7  8 | 0.0042  0.0034 | 0.0038 |
| 00000018242  (G3URD8)  00000005222  (G1N172) | IPI00598406  ASSY; [8] | Argininosuccinate synthase | A  B | | S,I  S | 4  4 | 0.0009  0.0005 | 0.0007 |
| 00000005240  (G1N187) | IPI00600823  F1NWM1 | Proteasome subunit α-type 7 | A  B | | **-**  **S**,I | -  2 | -  0.0004 |  |
| 00000005255  (G1N198) | IPI00574572  F125A | Multivesicular body subunit 12A/FAM125A | A  B | | **-**  **S**,I | -  2 | -  0.0004 |  |
| 00000005297  (G1N1C7) | IPI00585707  Q5ZMT1; [8] | Similar to Rho GDP-dissociation inhibitor 1 | A  B | | **S**,I  S | 6  5 | 0.0041  0.0053 | 0.0094 |
| 00000005306  (G1N1D3) | F1NWN4 | Uncharacterized protein/similar to fibulin 2 | A  B | | S,**I**  I | 3  4 | 0.0003  0.0002 | 0.0003 |
| 00000005322  (G1N1E5)  00000017298  (G3UP12) | IPI00596673  PDIA1;[8] | Protein disulfide isomerase | A  B | | S,**I**  S,I | 13  14 | 0.0089  0.0128 | 0.0109 |
| 00000005336  (G1N1F6)  00000018238  (G3URD4) | IPI00682826  Q8QFX0, Q5F3N4 | Disintegrin metalloprotease ADAM10 | A  B | | S,I  **S**,I | 7  5 | 0.0025  0.0016 | 0.0021 |
| 00000005351  (G1N1G9)  00000005342  (G1N1G2) | IPI00591422  CAPZB | F-actin capping protein subunit β | A  B | | **-**  S | -  3 | -  0.0006 |  |
| 00000005364  (G1N1H6) | IPI00583980  F1NN85; [8] | Uncharacterized proteins/similar to C3 and PZP-like α2-macroglobulin domain-containing protein 8 | A  B | | **S**,I  **S**,I | 19  14 | 0.0015  0.0009 | 0.0012 |
| 00000005367  (G1N1H9) | IPI00577103  F1NGN7 | Uncharacterized protein/similar to UDP-N-acetyl-α-D-galactose polypeptide N-acetyl-galactosaminyltransferase 14 | A  B | | S,**I**  S,I | 3  2 | 0.0014  0.0004 | 0.0009 |
| 00000005404  (G1N1K4) | IPI00572918  F1NZV7; [8,17] | Uncharacterized protein/similar to megakaryocyte potentiating factor (mesothelin); domain: mesothelin | A  B | | S  S | 7  9 | 0.0008  0.0012 | 0.0010 |
| 00000005483  (G1N1R1) | IPI00576813  Q5ZJT3; [8] | Uncharacterized protein/similar to palmitoyl-protein thioesterase 1 | A  B | | S,I  **S**,I | 9  8 | 0.0160  0.0173 | 0.0167 |
| 00000005496  (G1N1S2)  00000019672  (G3UV09) | E1C3K3  IPI00819936  F1NYL7 | Ribosomal protein L11 | A  B | | -  S | -  2 | -  0.0005 |  |
| 00000005509  (G1N1T0) | IPI00963757  E1BST6 | Similar to voltage-dependent calcium channel subunit α2 | A  B | | -  S | -  5 | -  0.0002 |  |
| 00000005528  (G1N1U5) | IPI00591099  F1NGM0 | EH domain-containing protein 3 (EHD3); shares 2 peptides with 00000011891 (G1NFT2) | A  B | | -  S | -  4 (5) | -  0.0007 |  |
| 00000005534  (G1N1V0) | IPI00577650  E1C7P4 | Uncharacterized protein; domains: Sushi/SCR/CCP, signal seq | A  B | | -  S | -  5 | -  0.0001 |  |
| 00000005571  (H9H0V5) | IPI00597630  H9KZC8 | Similar to proliferation-associated protein 1 | A  B | | -  S | -  3 | -  0.0002 |  |
| 00000005584  (G1N1Y6) | IPI00590719  E1C7A7, O12945; [8] | Vitronectin | A  B | | S,I  **S**,I | 7  5 | 0.0347  0.0838 | 0.0593 |
| 00000005615  (G1N210) | IPI00585476  F1NZZ2, LAMA5; [8] | Laminin α5 | A  B | | S,I  S,I | 62  61 | 0.0064  0.0077 | 0.0071 |
| 00000005644  (G1N231) | IPI00579078  Q8UWG7 | Ribosomal protein L6 | A  B | | **-**  S | -  2 | -  0.0002 |  |
| 00000005649  (G1N234) | IPI00578016  DKK3; [8,9,16] | Dickkopf-related protein 3 | A  B | | **S**,I  **S**,I | 6  4 | 0.0074  0.0054 | 0.0064 |
| 00000005684  (G1N260) | IPI00596714  F1P4X3; [8] | Similar to tissue α-L-fucosidase | A  B | | -  S,I | -  2 | -  0.0009 |  |
| 00000005723  (G1N291) | IPI00588015;  Q8QFQ7; [8] | Cytokine receptor β | A  B | | S  S | 4  6 | 0.0010  0.0023 | 0.0017 |
| 00000005746  (H9H0W2) | Q90WR3; [8] | Hemopexin, fragment | A  B | | **S**,I  **S**,I | 9  10 | 0.6960  0.9002 | 0.7981 |
| 00000005882  (G1N2K9) | IPI00572470  Q6QAZ, F1N9S7; [8] | Annexin A1 | A  B | | **S**,I  **S**,I | 6  7 | 0.0121  0.0154 | 0.0138 |
| 00000005955  (G1N2S0)  00000017737  (G3UQ69)  00000019301  (G3UU53) | IPI00585421  F1N888  IPI00591365  F1NWE4  [8] | Protein-tyrosine phosphatase CRYPα1  Receptor-type protein-tyrosine phosphatase S;  Protein-tyrosine phosphatase CRYPα2, fragment | A  B | | **S**,I  **S**,I | 6 (12)  7 (13) | 0.0019  0.0024 | 0.0022 |
| 00000005960  (G1N2S5) | IPI00573416  Q5ZJN6; [8] | Similar to soluble calcium-activated nuclease 1, fragment | A  B | | S,I  S,I | 5  6 | 0.0054  0.0090 | 0.0072 |
| 00000005964  (G1N2S7) | IPI00572786  E1C8K1; [8,16] | Uncharacterized protein/similar to nephronectin | A  B | | S,I  **S**,I | 18  16 | 0.0198  0.0208 | 0.0203 |
| 00000005966  (G1N2S9) | IPI00596301  F1N9F4 | Similar to δ-notch-like EGF repeat-containing transmembrane protein | A  B | | -  S | -  2 | -  0.0005 |  |
| 00000005995  (G1N2V2) | IPI00586101  D2D3P4 | Rab27a | A  B | | I  S,**I** | 2  2 | 0.0002  0.0002 | 0.0002 |
| 00000006030  (H9H0X6) | IPI00587741 | Uncharacterized protein/similar to lysosomal protein NUC-G1-A | A  B | | **S**,I  **S**,I | 3  3 | 0.0064  0.0077 | 0.0071 |
| 00000017273  (G3UNZ0)  00000006046  (G1N2Z5) | IPI00599415  SPRC; [8] | SPARC/BM-40/Osteonectin | A  B | | S,**I**  **S**,I | 8  9 | 0.3852  0.7469 | 0.5661 |
| 00000006061  (G1N308) | IPI00577281  F1NG93 | Acid α-glucosidase | A  B | | S,**I**  **S**,I | 5  5 | 0.0004  0.0012 | 0.0008 |
| 00000006063  (H9H0X7) | - | Similar to T-complex protein 1 subunit γ | A  B | | -  S | -  3 | -  <0.0001 |  |
| 00000006089  (G1N330) | IPI00595463  NRP1; [8] | Neuropilin-1 | A  B | | S,I  **S**,I | 6  8 | 0.0023  0.0024 | 0.0024 |
| 00000006093  (G1N333) | F1NGI6 | Uncharacterized protein/similar to N-sulphoglucosamine sulphohydrolase | A  B | | S,I  **S**,I | 2  3 | 0.0011  0.0027 | 0.0019 |
| 00000006099  (H9H0X9) | IPI00599279  S10AB; [8] | S100-A11/calgizzarin | A  B | | S,I  S | 2  3 | 0.0053  0.0060 | 0.0057 |
| 00000006122  (G1N356) | IPI00576308  E1BYS4 | Carboxypeptidase D | A  B | | S,I  S,I | 5  6 | 0.0006  0.0009 | 0.0008 |
| 00000006131  (G1N365) | IPI00589360  F1NPJ8; [8,17] | Glutathione peroxidase 3 | A  B | | **S**,I  **S**,I | 6  6 | 0.0253  0.0586 | 0.0420 |
| 00000006152  (G1N380) | IPI00574055  PLOD1; [8,16] | Procollagen-lysine 2-oxoglutarate 5-dioxygenase | A  B | | S,**I**  S,**I** | 26 (27)  26 (27) | 0.1055  0.1429 | 0.1242 |
| 00000006187  (G1N3B0) | IPI00587080  Q5ZKO2; [8] | Uncharacterized protein/similar to GM2 activator protein | A  B | | **S**,I  S | 5  6 | 0.0362  0.0369 | 0.0366 |
| 00000006222  (G1N3D8) | IPI00574153  F1NU17, PGK | Phosphoglycerate kinase, fragment | A  B | | **-**  **S**,I | -  4 | -  0.0010 |  |
| 00000006253  (G1N3G3) | IPI00584377  IPI01017235  UROK | Urokinase-type plasminogen activator | A  B | | **S**,I  S | 5  4 | 0.0017  0.0005 | 0.0011 |
| 00000006303  (G1N3K4) | IPI00684854  IPI00593692  E1C453 | Similar to heterogeneous nuclear ribonucleoprotein K | A  B | | -  S | -  3 | -  0.0002 |  |
| 00000006393  (G1N3R8) | IPI00576877  F1NUU6 | Similar to ectonucleotide pyrophosphatase/ phosphodiesterase family member 6 | A  B | | S,I  **S**,I | 2  2 | 0.0024  0.0033 | 0.0029 |
| 00000006439  (G1N3W0) | IPI00602477  EPHB2; [8] | Ephrin type B receptor 2 | A  B | | **S**,I  **S**,I | 7  4 | 0.0008  0.0006 | 0.0007 |
| 00000006474  (H9H102) | IPI00573347  H9KZ68; [8] | Uncharacterized protein/similar to TMEM132A | A  B | | S,**I**  **S**,I | 6  6 | 0.0106  0.0131 | 0.0119 |
| 00000006525  (G1N423) | IPI00586613  FUT11 | α-1,3-fucosyltransferase 11 | A  B | | S,I  S,**I** | 2  2 | 0.0010  0.0009 | 0.0010 |
| 00000006532  (G1N427) | IPI00599980  CRDL1 | Chordin-like protein 1 | A  B | | **S**,I  **S**,I | 14  14 | 0.0084  0.0089 | 0.0087 |
| 00000006572  (G1N454) | IPI00601828  E1BSQ1; [8] | Similar to secretogranin-3 | A  B | | -  S | -  2 | -  0.0002 |  |
| 00000006579  (G1N459) | IPI00596504  Q5ZIW8 | Similar to Ras-like protein TC21, fragment | A  B | | S,I  S | 3  3 | 0.0008  0.0007 | 0.0008 |
| 00000006599  (G1N475) | E1C7E8 | Uncharacterized protein/similar to ADAMTS13; domains: CUB, TSP_1, ADAM/reprolysin | A  B | | S  S | 2  3 | 0.0001  0.0001 | 0.0001 |
| 00000006622  (G1N490) | IPI00583085  IPI00820520  F1NPG4, F1NKF0,  E1C7E8 | Uncharacterized protein; domains: Leucine-rich repeat, Fn3, signal seq | A  B | | S  **S**,I | 4  4 | 0.0027  0.0027 | 0.0027 |
| 00000006673  (G1N4C8) | IPI00583368  F1NXB0; [8] | Similar to procollagen-lysine 2-oxoglutarate 5-dioxygenase, fragment (PLOD2) | A  B | | **S**,I  **S**,I | 8  12 (13) | 0.0018  0.0024 | 0.0021 |
| 00000006692  (G1N4E3) | IPI00570636  F1NX66; [8] | Similar to phospholipid scramblase 1 | A  B | | S,I  **S**,I | 3  2 | 0.0035  0.0089 | 0.0062 |
| 00000006732  (G1N4G8)  00000018320  (G3URL1) | IPI00578831  F1NG82  IPI00576318  Q90574 | Similar to filamin(-B) | A  B | | -  S | -  10 | -  <0.0001 |  |
| 00000006759  (G1N4I7) | IPI602255  F1NYJ1; [8] | Similar to cathepsin L | A  B | | S,I  S,**I** | 5  6 | 0.0192  0.0273 | 0.0235 |
| 00000006766  (G1N4J2) | IPI00588616  Q5ZJG0; [8] | Uncharacterized protein/similar to N(4)-(β-N-acetylglucosaminyl)-L-asparaginase | A  B | | S,I  S,I | 7  7 | 0.0123  0.0172 | 0.0148 |
| 00000006811  (G1N4M6) | IPI00589473  E1BUB7 | Similar to olfactomedin-3/Noelin-3 | A  B | | I  - | 2  - | 0.0006  - |  |
| 00000006852  (G1N4Q7) | IPI00581215  E1BZR7; [8] | Uncharacterized protein/similar to cadherin-related family member 3; domains: cadherin, signal seq | A  B | | **S**,I  **S**,I | 12  11 | 0.0034  0.0031 | 0.0033 |
| 00000006872  (G1N4S3) | IPI00598959  CDC42; [8] | Similar to cell division control protein 42 homolog | A  B | | S,**I**  **S**,I | 3  4 | 0.0016  0.0013 | 0.0015 |
| 00000006900  (G1N4U8) | IPI00598223  F1NCY6 | Similar to tumor growth factor receptor superfamily member 6B/decoy receptor 3; | A  B | | **S**,I  **S**,I | 6  4 | 0.0139  0.0108 | 0.0124 |
| 00000006912  (G1N4V8) | IPI00683794  F1N868 | Uncharacterized protein/similar to PDGF receptor β | A  B | | **-**  S | -  2 | -  0.0002 |  |
| 00000006934  (G1N4X8) | IPI00574964  RS13, F1NGB4; [8] | Ribosomal protein S13 | A  B | | **-**  S | -  2 | -  0.0005 |  |
| 00000006977  (G1N506) | IPI00593825  E1BS68; [8] | Similar to putative N-acetylglucosamine-1- phosphotransferase γ-subunit | A  B | | **S**,I  **S**,I | 8  7 | 0.0100  0.0323 | 0.0212 |
| 00000006990  (G1N515) | IPI00602594  DEST; [8] | Destrin | A  B | | S,**I**  S,I | 4  3 | 0.0009  0.0011 | 0.0010 |
| 00000007002  (G1N522) | IPI00576782  CYT¸ [8,9,17] | Cystatin | A  B | | **S**,I  **S**,I | 9  10 | 1.8101  1.5303 | 1.6702 |
| 00000007021  (G1N538) | IPI00573727  Q5ZHR1; [8,9,16,17] | Uncharacterized protein/similar to nucleobindin-2; domains: EFh, signal seq | A  B | | **S**,I  **S**,I | 24  30 | 0.1924  0.2733 | 0.2329 |
| 00000007097  (G1N591) | IPI00600265  Q5G8Y9; [8] | Apolipoprotein D | A  B | | **S**,I  **S**,I | 6  8 | 0.4913  0.4696 | 0.0405 |
| 00000007110  (G1N5A0) | IPI00593582  F1NNE7 | Uncharacterized protein/similar to ER aminopeptidase 1 (ERAP1)/similar to oxytocinase | A  B | | S  S | 3  5 | 0.0002  0.0002 | 0.0002 |
| 00000007128  (G1N5B2) | F1NF38; [8] | Similar to xyloside xylosyltransferase 1; 76% identity to IPI00596356 | A  B | | **S**,I  **S**,I | 4  3 | 0.0025  0.0038 | 0.0032 |
| 00000007143  (G1N5C3) | - | Uncharacterized protein/similar to Sdcbp2; domains: PDZ | A  B | | S,I  S | 3  2 | 0.0009  0.0004 | 0.0007 |
| 00000007181  (G1N5F9) | IPI00601953  E1C7C6 | Similar to VEGF-C | A  B | | **-**  **S**,I | -  2 | -  0.0009 |  |
| 00000007187  (G1N5G4) | IPI00577421  CH60 | Mitochondrial HSP60 | A  B | | **-**  S | -  3 | -  <0.0001 |  |
| 00000007193  (G1N5G9) | IPI00600713  F1NBA6, partial; [8] | Similar to collagen α1 (VII) **^1^** | A  B | | **S**,I  **S**,I | 24  27 | 0.0020  0.0020 | 0.0020 |
| 00000007268  (G1N5M2)  00000019255 | IPI00819550  E1BJQ8 | Uncharacterized protein/similar to ectonucleoside triphosphate diphosphohydrolase 6 | A  B | | S,I  **S**,I | 6  4 | 0.0029  0.0013 | 0.0021 |
| 00000007318  (G1N5R3) | IPI00601244  E1C0F3 | Similar to Ras-related protein Rab-7a | A  B | | **S**,I  S | 3  6 | 0.0007  0.0010 | 0.0009 |
| 00000007322  (G1N5R7) | IPI00582670  F1N901; [8] | Similar to α-1,3-mannosyl-glycoprotein 4-β-N-acetylglucosaminyltransferase B | A  B | | S,**I**  S,I | 5  5 | 0.0007  0.0008 | 0.0008 |
| 00000007329  (G1N5S3) 00000007364 (G1N5U9) | AT134 | Probable cation-transporting ATPase 13A4 | A  B | | **-**  S,**I** | -  5 | -  0.0003 |  |
| 00000007343  (G1N5T4) | IPI00577021  LAC; [8,16] | Similar to immunoglobulin light chain; shares peptides with other Ig light chains | A  B | | **S**,I  **S**,I | 3 (11)  3 (11) | 0.2038  0.5635 | 0.3837 |
| 00000007361  (G1N5U6) | IPI00574703  F1NC22; [8] | Similar to immunoglobulin light chain LV1_CHICK; shares peptides with other Ig light chains | A  B | | **S**,I  **S**,I | 1 (3)  1 (2) | 0.0961  0.0240 | 0.0600 |
| 00000007369  (G1N5V3) | IPI00597128  F1NMD3; [8] | Similar to polypeptide N-acetylgalactosaminyltransferase 12; shares peptide with 00000016636 | A  B | | **-**  S,I | -  2 | -  0.0017 |  |
| 00000007411  (G1N5Y2) | IPI00599712  F1NYG4; [8] | Similar to β2-glycoprotein/apolipoprotein H; domains: Sushi/SCR/CCP, β2-glycoprotein 5^th^ domain, signal seq | A B | | S  S | 6  5 | 0.0030  0.0022 | 0.0026 |
| 00000007430  (G3US27) | IPI00597846  Q6KDZ1, F1NAT6  [8,17] | Similar to basement-specific heparan sulfate proteoglycan core protein; domains: LamBIV, EGF, LamG, LDL A repeats, IG, Con A/glucanase | A  B | | S,I  S,I | 33  37 | 0.0022  0.0026 | 0.0024 |
| 00000007459  (G1N611) | IPI01027931  F1P3J1 | Similar to clathrin heavy chain 1 | A  B | | -  **S**,I | -  21 | -  0.0004 |  |
| 00000007470  (G1N620) | IPI00684493  F1NJJ4,Q5ZM95 | Heterogeneous nuclear riboprotein H1-like protein | A  B | | -  S | -  3 | -  0.0003 |  |
| 00000007542  (G1N679) | IPI00584727  LDHA; [8] | L-lactate dehydrogenase | A  B | | -  S | -  2 | -  0.0015 |  |
| 00000007552  (G1N684) | IPI00576103  E1C117; [8] | Uncharacterized protein/similar to interleukin-1 receptor accessory protein | A  B | | **S**,I  **S**,I | 13  14 | 0.0401  0.0588 | 0.0495 |
| 00000007568  (G1N693) | IPI00594717  F1NKJ8, Q5F366 | Uncharacterized protein/similar to α-L-iduronidase | A  B | | **S**,I  **S**,I | 5  8 | 0.0013  0.0022 | 0.0018 |
| 00000007574  (G1N698) | IPI00592920  E1C7R1 | Uncharacterized protein/similar to EGF-like domain 7; domains: EGF, EMI | A  B | | I  I | 3  2 | 0.0008  0.0005 | 0.0007 |
| 00000007579  (G1N6A3) | IPI00588611  E1BWW2; [8] | Similar to tumor susceptibility protein 101 | A  B | | S,I  S | 2  3 | 0.0008  0.0004 | 0.0006 |
| 00000007603  (G1N6B7) | IPI00585225  F1NGT0 | Uncharacterized protein/similar to tissue factor | A  B | | **-**  S | -  2 | -  0.0004 |  |
| 00000007635  (G1N6D8) | IPI00585901  OSTP; [13,8,9] | Osteopontin | A  B | | **S**,I  **S**,I | 3  4 | 0.1688  0.1228 | 0.1458 |
| 00000007641  (G1N6E1) | IPI00581368  OC116; [38,39,8,9,16,17] | Ovocleidin-116 | A  B | | S,I  **S**,I | 40  37 | 33.8482  28.7611 | 31.3047 |
| 00000007676  (G1N6G8) | IPI00579399  F1NSM1 | Uncharacterized protein/similar to mitochondrial ADP-ribose pyrophosphatase | A  B | | **-**  S,I | -  3 | -  0.0006 |  |
| 00000007710  (G1N6J5) | IPI00577031  F1NHD8; [8] | Similar to programmed cell death protein 6/NUDIX | A  B | | **S**,I  **S**,I | 6  4 | 0.0139  0.0087 | 0.0113 |
| 00000007738  (G1N6L3) | IPI0000584299  PTPRJ; [8] | Similar to receptor-type tyrosine-protein phosphatase η | A  B | | S  **S**,I | 2  3 | <0.0001  0.0002 | 0.0001 |
| 00000007758  (H9H1A1) | IPI00576601  Q5ZLB3 | Similar to heterogeneous nuclear ribonucleoprotein A1 | A  B | | -  S | -  3 | -  0.0002 |  |
| 00000007759  (G1N6M8) | IPI00573506  Q53HW8;  [52,8,9,17] | Ovocalyxin-36 | A  B | | S,**I**  **S**,I | 17  14 | 6.3639  5.6336 | 5.9990 |
| 00000007776  (H9H1A3) | IPI00574916  Q2I811 | Antimicrobial peptide NK-lysin | A  B | | S  S | 3  3 | 0.0123  0.0123 | 0.0123 |
| 00000007777  (G1N6P2) | IPI00598229  TENP; [8] | TENP | A  B | | S,**I**  S,I | 5  3 | 0.0152  0.0046 | 0.0099 |
| 00000007823  (G1N6S6) | IPI00577734  F1NW57 | Similar to N-acetylgalactosamine-6-sulfatase | A  B | | **-**  S,I | -  2 | -  0.0003 |  |
| 00000007843  (G1N6T9) | IPI00571064  Q6IVU9 | Similar to xylosyltransferase I | A  B | | **S**,I  **S**,I | 3  4 | 0.0003  0.0003 | 0.0003 |
| 00000007855  (G1N6U7) | IPI00573925  E1BSL7; [8] | Uncharacterized protein/similar to ER thioredoxin domain-containing protein 4 | A  B | | S,I  **S**,I | 3  5 | 0.0006  0.0008 | 0.0007 |
| 00000007872  (G1N6V8) | IPI00584215  F1NXW3 | Similar to ribosomal protein S15 | A  B | | **-**  S | -  3 | -  0.0010 |  |
| 00000007908  (G1N6Y5) | IPI00578482  E1C004; [8] | Uncharacterized protein; domain: α-carbonic anhydrase | A  B | | **S**,I  **S**,I | 15  16 | 0.2348  0.4722 | 0.3535 |
| 00000007940  (G1N704) | IPI00575515  TFR1 | Transferrin receptor protein 1 | A  B | | **-**  **S**,I | -  3 | -  0.0003 |  |
| 00000007966  (G1N722) | IPI00583355  E1BXW5; [8] | Uncharacterized protein/similar to β-microseminoprotein | A  B | | **S**,I  **S**,I | 3  3 | 0.0036  0.0097 | 0.0067 |
| 00000007971  (G1N724) | IPI00584241  E1BYA2 | Uncharacterized protein/similar to GLI pathogenesis-related 2 | A  B | | I  - | 2  - | 0.0007  - |  |
| 00000007984  (G1N734) | IPI00580779  Q5ZL03 | Uncharacterized protein/similar to glutamate-cysteine ligase regulatory subunit | A  B | | -  S | -  2 | -  0.0005 |  |
| 00000007994  (G1N743) | IPI00596947  F1NXX9 | Uncharacterized protein/similar to bactericidal permeability-increasing protein; domains: Lipid-binding serum glycoprotein N-term, BPI α/β, signal seq | A  B | | S,**I**  **S**,I | 11  13 | 0.0043  0.0074 | 0.0059 |
| 00000008002  (G1N751) | IPI00582056  GELS, F1NKF3; [8] | Gelsolin | A  B | | **S**,I  **S**,I | 20 (21)  21 (22) | 0.0489  0.0411 | 0.0450 |
| 00000008014  (G1N762) | IPI00585409  E1C8K3; [8,17] | Annexin (A8) | A  B | | S,I  S | 8  8 | 0.0008  0.0012 | 0.0010 |
| 00000008030  (G1N774) | IPI00594471  E1BTV1; [8] | Similar to stomatin | A  B | | S,**I**  **S**,I | 9  8 | 0.0229  0.0167 | 0.0198 |
| 00000008069  (H9H1C7) | - | Uncharacterized protein/similar to VH1 | A  B | | S,**I**  **S**,I | 2  1 (2) | 0.0190  0.0270 | 0.0230 |
| 00000008080  (G1N7A9) | IPI00593455  CADH1; [8] | Cadherin-1 | A  B | | **S**,I  S | 2  4 | 0.0009  0.0013 | 0.0011 |
| 00000008129  (Q5MGT1) | IPI00592767  IGFII; [8] | IGF-II | A  B | | **S**,I  **S**,I | 5  7 | 0.0420  0.0535 | 0.0478 |
| 00000008137  (G1N7F3) | IPI00584343  F1ND63 | Uncharacterized protein/similar to ADAMTS-3 | A  B | | -  S,I | -  4 | -  0.0002 |  |
| 00000008151  (H9H1D0) | IPI00576957  - | Uncharacterized protein/similar to sperm flagellar protein 1 | A  B | | I  - | 3  - | 0.0004  - |  |
| 00000008196  (G1N7J7) | IPI0000580023  F1NB92; [8] | Uncharacterized protein/similar to ER aminopeptidase 1 | A  B | | **S**,I  **S**,I | 6  9 (11) | 0.0007  0.0017 | 0.0012 |
| 00000008230  (G1N7M9) | IPI00818680  IPI00597667  F1NCW4; Q5ZIH1 | Uncharacterized protein/similar to heterogeneous nuclear ribonucleoprotein D0 | A  B | | -  S | -  2 | -  0.0002 |  |
| 00000008266  (G1N7Q8) | IPI00577274  O42390; [8] | RGD-CAP; domains: FAS1,EMI, TGF-β-induced protein bIGH3/osteoblast-specific factor 2 | A  B | | S,I  **S**,I | 6  4 | 0.0006  0.0003 | 0.0005 |
| 00000008278  (G1N7R6) | IPI00819636  HPSE | Heparanase | A  B | | S,**I**  - | 3  - | 0.0001  - |  |
| 00000008288  (G1N7S6)  00000018452  (G3URY4)  00000019502  (G3UUN5) | IPI00602099  MIM1, F1NEF7  IPI00821500  F1NXC3 | Myeloid protein 1 | A  B | | -  S,I | -  3 | -  0.007 |  |
| 00000008304  (G1N7U1) | IPI0057699  F1P1G6; [8] | Uncharacterized protein/similar to KIAA1199; domains: parallel β-helix repeat, pectin lyase fold, G8, mucin2_WxxW | A  B | | **S**,I  **S**,I | 48  56 | 0.0548  0.0942 | 0.0745 |
| 00000008314  (G1N7V1) | IPI00577831  F1NU54; [8] | Similar to β-1,3-N-acetylglucosaminyl-transferase | A  B | | S,I  **S**,I | 8  9 | 0.0136  0.0150 | 0.0143 |
| 00000008317  (G1N7V4) | IPI00587410  METL9 | Methyltransferase-like protein 9 | A  B | | I  - | 3  - | 0.0004  - |  |
| 00000008334  (G1N7W8)  00000019449  (G3UUI5) | IPI00601433  Q6T7B9  IPI00683119  Q6T7C0 | Stromal cell-derived factor-1β | A  B | | S,I  S | 2  2 | 0.0034  0.0026 | 0.0040 |
| 00000008383  (G1N804)  00000019053  (G3UTK4) | IPI00582660  H2AY  IPI00599073  O93327-2 | Histone H2A.1 | A  B | | -  S | -  3 | -  0.0001 |  |
| 00000008424  (G1N836) | IPI00596335  Q5F3W4; [8] | Uncharacterized protein/similar to retinoic-inducible serine carboxypeptidase (RISC) | A  B | | -  S,I | -  2 | -  0.0005 |  |
| 00000008444  (G1N850) | IPI00571529  F1N8D9; [8] | Similar to Ras-related C3 botulinum toxin substrate 1 | A  B | | -  **S**,I | -  3 | -  0.0035 |  |
| 00000008524  (G1N8A7) | IPI00571247  Q5ZIJ5; [8] | Uncharacterized protein/similar to cathepsin A; domains:peptidase S10 | A  B | | S,**I**  S,I | 10  10 | 0.0856  0.0753 | 0.0805 |
| 00000008532  (G1N8B3) | IPI00585812  F1P0X8; [8,9] | Similar to olfactomedin-like protein 2A | A  B | | S,**I**  - | 2  - | 0.0045  - |  |
| 00000008584  (G1N8F1) | IPI00578086  C5H3Z3,  F1NIL5; [8] | Phospholipid transfer protein; domains: Lipid-binding serum protein/BPI, signal seq | A  B | | **S**,I  **S**,I | 4  4 | 0.0009  0.0024 | 0.0017 |
| 00000008598  (G1N8G3)  00000008600  (G1N8G4) | IPI00592906  Q90998; [8] | Transforming growth factor-β type III receptor; domains: ZP2, endoglin/CD105 antigen, signal seq | A  B | | **S**,I  **S**,I | 13  11 | 0.0132  0.0105 | 0.0119 |
| 00000008610  (G1N8H3) | IPI00586861  F1P010; [8] | Uncharacterized protein/similar to actin-related protein 2/3 complex subunit 4 | A  B | | **-**  **S**,I | -  3 | -  0.0017 |  |
| 00000008653  (G1N8K5) | IPI00571254  F1NNV6; [8] | Uncharacterized protein; domain: ATPase V1 complex subunit S1 | A  B | | **S**,I  S | 4  3 | 0.0037  0.0014 | 0.0026 |
| 00000008667  (G1N8L7) | IPI00593485  Q5ZM28 | Similar to sorcin; domains: EFh | A  B | | S,I  S | 3  2 | 0.0009  0.0009 | 0.0009 |
| 00000008686  (G1N8M9) | IPI00571280  F1NVP4; [8] | Similar to IGFBP-7 | A  B | | **S**,I  **S**,I | 6  7 | 0.0596  0.0704 | 0.065 |
| 00000008705  (G1N8P0) | IPI00600003  G1N8P0, CATD; [8] | Cathepsin D | A  B | | S,**I**  **S**,I | 16  17 | 0.1631  0.2813 | 0.2222 |
| 00000008719  (G1N8Q2) | IPI00590301  E1BYN7 | Similar to voltage-dependent anion-selective channel protein 1 (VDAC1) | A  B | | -  S | -  3 | -  0.0002 |  |
| 00000008735  (G1N8R5) | IPI00590375  GRP78; [8] | HSP70/78kDa glucose- regulated protein ; shares 2 peptides with 00000001450 (G1MSW3) | A  B | | S,**I**  S,**I** | 10  15 | 0.0088  0.0192 | 0.0140 |
| 00000008754  (G1N8S9)  00000008758  (G1N8T1) | IPI00601877  NDK | Nucleoside diphosphate kinase; shares 1 of 4 peptides with 00000010144 (G1NBU7) | A  B | | S,I  **S**,I | 3 (4)  4 (5) | 0.0050  0.0063 | 0.0057 |
| 00000017525  (G3UPM4)  00000008775  (G1N8U4) | IPI00594689  F1P4D5 | Uncharacterized protein; domain: TMEM66 | A  B | | **S**,I  S | 4  4 | 0.0023  0.0024 | 0.0024 |
| 00000008807  (G1N8W9) | IPI00599118  F1N872 | Similar to malectin | A  B | | S,**I**  S,I | 4  4 | 0.0017  0.0011 | 0.0014 |
| 00000008824  (G1N8Y5/  Q5GMQ7) | IPI00593624  F1P297; [8] | Kazal-type serine protease inhibitor | A  B | | **S**,I  S | 3  2 | 0.0073  0.0032 | 0.0053 |
| 00000008830  (G1N8Z1)  00000008881  (G1N931) | IPI00585604  E1C037; [8,9,16] | Uncharacterized protein/similar to mucin 5AC, fragments | A  B | | S,I  S,I | 55  47 | 0.1483  0.1494 | 0.1489 |
| 00000008878  (G1N928) | IPI00602183  F1NN57 | Similar to mucin | A  B | | S,I  S,I | 37  36 (37) | 0.0379  0.0409 | 0.0394 |
| 00000008901  (G1N943) | IPI00595834  E1BWA9; [8] | Similar to FAM3C | A  B | | S,I  **S**,I | 6  8 | 0.0034  0.0038 | 0.0036 |
| 00000008902  (G1N944) | IPI00575013  E1C0K5; [8,16] | Uncharacterized protein/milk fat globule EGF factor 8 protein; domains: EGF, coagulation factor 5/8, galactose-binding-like | A  B | | S,I  S,I | 29  26 | 1.1838  1.3120 | 1.2479 |
| 00000008914  (G1N954) | IPI00580591  E1BUN0; [8] | Uncharacterized protein/similar to hyaluronan and proteoglycan link protein 3 | A  B | | S,**I**  S,I | 15  16 | 0.0501  0.0572 | 0.0537 |
| 00000008936  (G1N969)  00000008375  (G1N7Z6) | IPI00595309  Q5F412, DYL1; [8] | Dynein light chain 1  Similar to dynein light chain 2;  Entries share several peptides | A  B | | **S**,I  S | 3  4 | 0.0042  0.0025 | 0.0034 |
| 00000008424  (G1N836) | IPI00596335  Q5F3W4; [8] | Uncharacterized protein/similar to retinoic-inducible serine carboxypeptidase (RISC) | A  B | | S,I  - | 2  - | 0.0005  - |  |
| 00000008941  (G1N972) | - | Similar to UDP-galactose:β-D-galactoside β-1,4-galactosyltransferase | A  B | | S,**I**  S,**I** | 4  4 | 0.0091  0.0083 | 0.0087 |
| 00000008961  (G1N988) | IPI00584991  MUC5B; [8,17] | Mucin-5B/ovomucin α-chain | A  B | | S,I  S,I | 32  30 | 0.0120  0.0117 | 0.0119 |
| 00000008986  (G1N9B0) | IPI00596430  E1C4M3 | Uncharacterized protein/similar to cathepsin Z | A  B | | **S**,I  **S**,I | 4  3 | 0.0037  0.0042 | 0.0040 |
| 00000008988  (G1N9B2) | IPI00592265  E1BVF2 | Uncharacterized protein; domains: proteinase inhibitor I2 Kunitz | A  B | | **-**  **S**,I | -  2 | -  0.0009 |  |
| 00000009067  (G1N9H7) | IPI00587452  E1C5S9; [8] | Uncharacterized protein/similar to FAM3D | A  B | | **S**,I  **S**,I | 6  5 | 0.0080  0:0067 | 0.0074 |
| 00000009068  (G1N9H8, HBA) | IPI00575926  HBA; [17] | Hemoglobin subunit α-A | A  B | | S,**I**  - | 3  - | 0.0017  - |  |
| 00000009081  (G1N9J0) | IPI00822901  F1NM72 | Similar to GNAS (Guanine nucleotide-binding protein G(s) subunit α) | A  B | | -  **S**,I | -  2 | -  0.0004 |  |
| 00000009120  (G1N9M2)  00000009127  (G1N9M7) | IPI00595654  PTPRG, F1NA27  [8] | Receptor-type tyrosine-protein phosphatase γ | A  B | | **S**,I  **S**,I | 6  7 | 0.0027  0.0034 | 0.0031 |
| 00000009145  (G1N9P0) | IPI00589800  Q5ZLF6 | Similar to Tmed7; domain: EMP24_GP25L/GOLD | A  B | | **-**  S,I | -  2 | -  0.0037 |  |
| 00000009158  (G1N9Q2) | IPI00578074  CYR61; [9] | CYR61 (cysteine-rich angiogenic inducer 61; CEF-10) | A  B | | **S**,I  **S**,I | 5  4 | 0.0014  0.0014 | 0.0014 |
| 00000009163  (G1N9Q6) | IPI00588222  RLA0 | Acidic ribosomal protein P0 | A  B | | -  S | -  3 | -  0.0006 |  |
| 00000009188  (G1N9S4) | IPI00573564  F1NER5; [8] | Uncharacterized protein/similar to chitinase domain-containing protein 1 | A  B | | S,**I**  S,**I** | 4  3 | 0.0018  0.0013 | 0.0016 |
| 00000009193  (H9H1I2) | IPI00592949  Q5ZJX9 | Proteasome subunit α-type | A  B | | -  S | -  2 | -  0.0002 |  |
| 00000009278  (G1N9Y6) | IPI00572857  APCD1 | Adenomatosis polyposis coli-downregulated 1 homolog (APCDD1) | A  B | | S,I  **S**,I | 9  15 | 0.0204  0.0299 | 0.0252 |
| 00000009321  (G1NA18) | IPI00578305  SNTAN; [8] | Sentan | A  B | | S,I  **S**,I | 6  6 | 0.0793  0.0523 | 0.0658 |
| 00000009349  (G1NA39) | IPI00595846  RGMA; [8] | Repulsive guidance molecule A | A  B | | **S**,I  **S**,I | 7  5 | 0.0019  0.0011 | 0.0015 |
| 00000018091  (G3UQZ4)  00000009421  (G1NA95) | IPI00818995  E1BW52 | Similar to insulin degrading enzyme | A  B | | **-**  S | -  2 | -  0.0009 |  |
| 00000009450  (G1NAC0)  00000019091  (G5E7Q4) | IPI00591299  F1NFL6, VIT2  [8,9,17] | Vitellogenin-2 | A  B | | **S**,I  **S**,I | 53  51 | 0.0185  0.0142 | 0.0164 |
| 00000009469  (G1NAD5) | IPI00596272  VIT3; [8] | Phosvitin/VTG3; shares 1 peptide with 00000009450 (G1NAC0) | A  B | | S,I  **S**,I | 8  7 | 0.0005  0.0003 | 0.0004 |
| 00000009474  (G1NAD9) | IPI00593654  F1NRM4; [8] | Uncharacterized protein/similar to di-N-acetylchitobiase | A  B | | S,I  S,I | 10  10 | 0.0482  0.0931 | 0.0707 |
| 00000009485  (G1NAE9) | IPI00577799  Q804X3; [8,9] | Similar to coagulation factor VIII; domains: coagulation factor 5/8, cupredoxin, multicopper oxidase, signal seq | A  B | | S,I  S,I | 7  15 | 0.0008  0.0014 | 0.0011 |
| 00000009518  (G1NAH6) | IPI00583049  F1NV86, Q5ZIX7 | Uncharacterized protein/similar to DNAJ-like subfamily B member 9 | A  B | | S,I  - | 4  - | 0.0006  - |  |
| 00000009632  (G1NAQ8) | IPI00589759  E1BZE1; [8,9] | Uncharacterized protein/similar to α2-HS-glycoprotein (Fetuin-A) | A  B | | **S**,I  S | 2  3 | 0.0144  0.0146 | 0.0145 |
| 00000009646  (G1NAR8) | IPI00584472  E1BQ57, Q9DE41  [8] | Similar to fetuin-B/Apo AI promoter B region-binding protein | A  B | | S,I  **S**,I | 3  4 | 0.0026  0.0023 | 0.0025 |
| 00000009670  (G1NAT8) | IPI00587003  E1C7T4 | Similar to proline arginine-rich and leucine-rich repeat protein (prolargin; PRELP) | A  B | S,I  S,I | | 5  5 | 0.0097  0.0101 |  |
| 00000009674  (G1NAU2) | IPI00595186  RNL2 | Liver ribonuclease/ribonuclease CL2; signal seq | A  B | | S  S | 3  5 | 0.0012  0.0053 | 0.0033 |
| 00000009689  (G1NAV2)  00000009685  (G1NAU9) | IPI00591843  VIT1; [8,17] | Vitellogenin-1 | A  B | | **S**,I  **S**,I | 36  33 | 0.0106  0.0091 | 0.0099 |
| 00000009693  (G1NAV5) | IPI00680631  IPI01017141  F1P161, F1NNP2 | TGF-β2 | A  B | | S,I  S | 2  3 | 0.0011  0.0014 | 0.0013 |
| 00000009749  (G1NB01) | IPI00585166  NMRL1 | NmrA-like family domain-containing 1 | A  B | | S,I  **S**,I | 7  6 | 0.0028  0.0023 | 0.0026 |
| 00000009803  (G1NB43) | IPI00579394  E1BT44;[8] | Uncharacterized protein/similar to α-galactosidase-A | A  B | | S,I  **S**,I | 4  5 | 0.0042  0.0062 | 0.0052 |
| 00000009812  (G1NB52)  00000009805  (G1NB45) | IPI00682727  F1NIN4  IPI00593218  MLRN | Myosin regulatory light chain 2 | A  B | | -  S | -  5 | -  0.0018 |  |
| 00000009949  (G1NBE9) | IPI00577639  F1NA58; [8] | Uncharacterized protein; domain: Serpin C1/protease inhibitor I4 (plasma protease C1 inhibitor) | A  B | | **S**,I  **S**,I | 18  20 | 0.1349  0.1919 | 0.1634 |
| 00000009990  (G1NBH6) | IPI00572815  F1NNI4 | Similar to glucoside xylosyltransferase 2 | A  B | | S,**I**  S,**I** | 5  4 | 0.0012  0.0017 | 0.0015 |
| 00000016369  (G1NR32)  00000009997  (G1NBI2) | IPI00581295  RS4 | Ribosomal protein S4 | A  B | | -  S | -  4 | -  0.0004 |  |
| 00000010021  (G1NBK1)  00000017462  (G3UPG7) | IPI00600561  O57465; [8,9,16] | Chordin | A  B | | **S**,I  **S**,I | 7 (30)  6 (33) | 0.2519  0.2290 | 0.2405 |
| 00000010072  (G1NBP3) | - | Similar to JmjC domain-containing protein 8 | A  B | | I  S,**I** | 4  4 | 0.0019  0.0032 | 0.0026 |
| 00000010137  (G1NBU2) | IPI00682836  E1C989 | Uncharacterized protein/similar to collagen α1 (XXVIII) **^1^** | A  B | | -  S | -  6 | -  0.0002 |  |
| 00000010144  (G1NBU7) | F1P3E1 | Nucleoside diphosphate kinase; shares 1 peptide with 00000008754 (G1N8S9) and  00000008758 (G1N8T1) | A  B | | S,**I**  S,**I** | 2  4 | 0.0006  0.0020 | 0.0013 |
| 00000010145  (G1NBU8) | IPI00595175  F1NUS6 | Uncharacterized protein/similar to NCAML1-like protein | A  B | | **S**,I  **S**,I | 13  21 | 0.0014  0.0023 | 0.0019 |
| 00000010162  (G1NBW3) | IPI00585486  PGAM1 | Phosphogycerate mutase 1 | A  B | | S,I  **S**,I | 3  3 | 0.0007  0.0029 | 0.0018 |
| 00000010188  (G1NBY2) | IPI00575596  Q5ZII1 | Similar to ribosomal protein L4 | A  B | | -  S | -  2 | -  0.0002 |  |
| 00000018368  (G3URQ6)  00000010222  (G1NC11) | IPI00575894  F1NED4  IPI00579666  F1NPY9; [8] | Similar to thrombospondin type-1 domain containing protein 7A; domains: TSP_1, EGF_like | A  B | | S  S | 10  17 | 0.0005  0.0008 | 0.0007 |
| 00000010305  (G1NC68) | IPI00579663  H9L3E5 | ATP synthase α-subunit | A  B | | **-**  **S**,I | -  5 | -  0.0008 |  |
| 00000010309  (G1NC71) | IPI00586930  F1NJ23; [8,16] | Laminin β1 | A  B | | **S**,I  S,I | 26  30 | 0.0069  0.0131 | 0.0100 |
| 00000010324  (G1NC80) | IPI00598274  Q6TPK5 | Schwann cell-specific EGF-like repeat autocrine factor | A  B | | **S**,I  **S**,I | 6  9 | 0.0238  0.0556 | 0.0397 |
| 00000010338  (G1NC90) | IPI00598054  E1BY93; [8] | Uncharacterized protein/similar to immunoglobin J chain | A  B | | S,**I**  **S**,I | 6  5 | 0.2261  0.0708 | 0.1485 |
| 00000010343  (G1NC93) | IPI00600666  Q92073 | Similar to β-1,4-galactosyltransferase | A  B | | S,I  **S**,I | 3  2 | 0.0016  0.0013 | 0.0015 |
| 00000017377  (G3UP85)  00000010350  (G1NCA0) | IPI00572250  ITA6 [8] | Integrin α6 light chain | A  B | | S,I  S,**I** | 6  7 | 0.0007  0.0009 | 0.0008 |
| 00000010367  (G1NCB5) | IPI00599851  CL049 [8] | UPF0454 protein C12orf49 homolog | A  B | | S  S | 3  3 | 0.0022  0.0014 | 0.0018 |
| 00000010377  (G1NCC1) | IPI00819358  IPI00603219  F1NF17, Q7T1F2 | Tumor necrosis factor-related apoptosis-inducing ligand | A  B | | I  - | 2  - | 0.0005  - |  |
| 00000010382  (G1NCC4) | IPI00599089  F1NYI5 | Uncharacterized protein; domains: Lamin_tail | A  B | | S,I  **S**,I | 2  4 | <0.0001  0.0001 | <0.0001 |
| 00000010386  (G1NCC8) | IPI0000603974  E1C234; [8] | Similar to sulfatase modifying factor 1 | A  B | | **S**,I  S,I | 3  3 | 0.0012  0.0018 | 0.0020 |
| 00000010418  (G1NCF1) | IPI00573327  Q9W6F5; [8] | Vitamin D-binding protein | A  B | | **S**,I  **S**,I | 24  25 | 0.1260  0.1725 | 0.1493 |
| 00000010498  (G1NCL1) | IPI00576481  F1P0Z3; [8] | Uncharacterized protein/similar to phospholipase B-like protein 2 | A  B | | S,I  S,I | 4  6 | 0.0031  0.0075 | 0.0053 |
| 00000010528  (G1NCN7) | IPI00599559  E1C937 | Uncharacterized protein/similar to anterior gradient (AGR) 2 | A  B | | -  S,I | -  2 | -  0.0028 |  |
| 00000010561  (G1NCR2) | IPI00574195  ALBU; [8,17] | Serum albumin | A  B | | **S**,I  **S**,I | 72  69 | 2.1639  2.9818 | 2.5729 |
| 00000010618  (G1NCV4) | IPI00597868  F1NQ51 | Uncharacterized protein/similar to laminin α3 chain | A  B | | S,I  **S**,I | 10  12 | 0.0002  0.0003 | 0.0003 |
| 00000010633  (G1NCW7) | IPI00575767  Q5ZMM4 | Catalase | A  B | | -  S,I | -  2 | -  <0.0001 |  |
| 00000010656  (G1NCY3) | IPI00598042  F1NHY7 | Similar to Wnt inhibitory factor 1 | A  B | | S,**I**  S,**I** | 3  3 | 0.0331  0.0239 | 0.0285 |
| 00000010659  (G1NCY4) | IPI00596585  CASC4; [8] | Similar to CASC4 | A  B | | S  S | 2  4 | 0.0007  0.0040 | 0.0024 |
| 00000010690  (G1ND08) | IPI00596266  F1NI04; [8] | Similar to glucosamine (N-acetyl)-6-sulfatase | A  B | | **S**,I  **S**,I | 9  11 | 0.0169  0.0544 | 0.0357 |
| 00000010715  (G1ND31)  00000018366  (G3URQ4) | IPI00571093  F1NL98  IPI00681851  Q9W6S4 | CD44-like | A  B | | **-**  S | -  2 | -  0.0002 |  |
| 00000010793  (G1ND93) | IPI00587346  NEUS; [8,17] | Uncharacterized protein/similar to serpin peptidase inhibitor clade 1 member 1/neuroserpin | A  B | | **S**,I  **S**,I | 11  13 | 0.0108  0.0261 | 0.0185 |
| 00000010810  (G1NDA4) | IPI00581471  PDC10 | Programmed cell death protein 10 | A  B | | -  S | -  2 | -  0.0003 |  |
| 00000010828  (G1NDB7) | IPI00580059  PRDX1; [8] | Peroxiredoxin-1; shares 1 peptide with 0000015525 (G1NP83) | A  B | | S,I  **S**,I | 5  6 | 0.0108  0.0146 | 0.01372 |
| 00000010836  (G1NDC2) | IPI00589103  E1BXE0 | Uncharacterized protein/similar to Golgi integral membrane protein 4 | A  B | | S  S | 5  3 | 0.0003  0.0005 | 0.0004 |
| 00000010843  (G1NDC6) | IPI00587782  CADH2; [8] | Cadherin-2 | A  B | | **S**,I  **S**,I | 7  8 | 0.0128  0.0169 | 0.0149 |
| 00000019582  (G3UUW3)  00000010848  (G1NDD0) | IPI00576336  PP1B, Q5ZL39 | Serine/threonine protein phosphatase | A  B | | **-**  S | -  3 | -  <0.0001 |  |
| 00000010864  (G1NDE2) | IPI00578479  H9KZZ3 | Similar to lysyl oxidase-like 3 | A  B | | **S**,I  S | 6  5 | 0.0031  0.0008 | 0.0020 |
| 00000010868  (G1NDE6) | IPI00598720  RAPB1 | Ras-related protein 1b | A  B | | S,**I**  **S**,I | 4  3 | 0.0016  0.0024 | 0.0020 |
| 00000010885  (G1NDF8) | IPI00598674  F1NJD7; [8] | Uncharacterized protein/similar to desmocollin-2 | A  B | | **S**,I  S | 5  3 | 0.0057  0.0038 | 0.0048 |
| 00000010909  (G1NDH9) | IPI00579109  AMPN; [8,17] | Aminopeptidase N (aminopeptidase Ey) | A  B | | **S**,I  **S**,I | 8  9 | 0.0465  0.0449 | 0.0457 |
| 00000010914  (G1NDI2) | IPI00598654  E1BTL4; [8] | Similar to protein phosphatase 1L | A  B | | S,**I**  S,I | 3  5 | 0.0020  0.0035 | 0.0028 |
| 00000010920  (G1NDI8) | IPI00577315  Q5F3S5; [8] | Similar to exostosin-2 | A  B | | **S**,I  S,I | 14  14 | 0.0043  0.0066 | 0.0055 |
| 00000010931  (G1NDJ8) | IPI00594746  RBP; [8] | Riboflavin-binding protein | A  B | | **S**,I  **S**,I | 9  6 | 0.0184  0.0140 | 0.0162 |
| 00000010932  (G1NDJ9) | IPI00590184  F1P103; [8] | Similar to calcium and integrin-binding protein 1 (calmyrin) | A  B | | S,**I**  **S**,I | 4  4 | 0.0102  0.0090 | 0.0096 |
| 00000010943  (G1NDK6) | IPI00579322  Q5ZKY7; [8] | Uncharacterized protein/similar to CD82 antigen isoform 1 | A  B | | S  - | 2  - | 0.0003  - |  |
| 00000010947  (G1NDL0) | IPI00575271  PDIA3 | Protein disulfide isomerase A3 | A  B | | S,**I**  S,I | 12  13 | 0.0027  0.0046 | 0.0037 |
| 00000010986  (G1NDP2)  00000010984  (G1NDP1) | IPI00593315  NPY; [8,9] | Pro-neuropeptide Y | A  B | | **S**,I  - | 2  - | 0.0061  - |  |
| 00000010985  (LYSC) | IPI00600859  B8YK79 [11,8,16,17] | Lysozyme C | A  B | | **S**,I  **S**,I | 11  11 | 0.6224  0.6670 | 0.6447 |
| 00000011001  (G1NDQ5) | IPI00594147  F5CE71, F5CE71 | Cathepsin H | A  B | | **S**,I  S,**I** | 5  5 | 0.0184  0.0274 | 0.0229 |
| 00000011013  (G1NDR4) | IPI00591809  E1C532; [8] | Similar to protein O-linked-mannose β-1,2-N-acetylglucosaminyltransferase 1 | A  B | | S,**I**  S,**I** | 12  12 | 0.0045  0.0035 | 0.0040 |
| 00000011014  (G1NDR5) | IPI00822137  E1BUM7 | α-(1,6)-fucosyltransferase | A  B | | S,**I**  S,I | 4  6 | 0.0015  0.0023 | 0.0019 |
| 00000011040  (G1NDT8) | IPI00587025  Q91000, F1NXT1; [8] | Furin | A  B | | **S**,I  **S**,I | 9  10 | 0.0106  0.0149 | 0.0128 |
| 00000011070  (G1NDW1)  00000019803  (G5E7Z2) | IPI00581402  COHA1; [8] | Collagen α1 (XVII) **^1^** | A  B | | S,I  S,I | 3  3 | 0.0012  0.0016 | 0.0014 |
| 00000011072  (G1NDW3) | IPI00651336  Q5ZME1 | Heterogeneous nuclear ribonucleoproteins A2/B1 | A  B | | -  S | -  2 | -  0.0005 |  |
| 00000011082  (G1NDX0) | IPI00593631  E1BX85; [8] | Uncharacterized protein/similar to glutathione S-transferase ω | A  B | | -  S | -  3 | -  0.0004 |  |
| 00000011091  (G1NDX8) | IPI00598019  Q5UMH8; [8] | Pentraxin-related protein 3 | A  B | | S,I  **S**,I | 8  8 | 0.0099  0.0107 | 0.0103 |
| 00000011100  (G1NDY6) | IPI00592470  ANXA5; [8] | Annexin A5 fragment | A  B | | S,I  **S**,I | 10  13 | 0.0030  0.0037 | 0.0034 |
| 00000011115  (G1NDZ9) | IPI00589637  F1N9Q2 | Similar to putative glyoxylase 1; domain: glyoxalase I | A  B | | **-**  S | -  2 | -  0.0003 |  |
| 00000011138  (G1NE21) | IPI00595787  F1NXU9; [8] | Uncharacterized protein/similar to α-mannosidase 2A2 | A  B | | **S**,I  **S**,I | 4  7 | 0.0001  0.0004 | 0.0003 |
| 00000011144  (G1NE26) | IPI00577417  H9KZF5; [8] | Uncharacterizted protein/similar to polypeptide N-acetylgalactosaminyltransferase 6; shares 1 peptide with 00000011222 (G1NE91) | A  B | | S,I  **S**,I | 3  4 | 0.0024  0.0025 | 0.0025 |
| 00000011175  (G1NE49) | IPI00575658  E1C316; [8] | Uncharacterized protein/similar to KIAA0494; domain: EFh | A  B | | **S**,I  S | 9  5 | 0.0025  0.0010 | 0.0018 |
| 00000011222  (G1NE91) | IPI00577417  Q5F4C7; [8] | Uncharacterizted protein/similar to polypeptide N-acetylgalactosaminyl-transferase 3 | A  B | | -  S,**I** | -  2 | -  0.0005 |  |
| 00000011247  (G1NEA9) | IPI00820593  F1NC33 | HSP90-β; shares peptides with 00000014116 (G1NKX1; HSP90-α) | A  B | | S,I  S | 2  7 (12) | 0.0018  0.0034 | 0.0026 |
| 00000011276  (G1NED2) | IPI00682636  F1N9U6 | Uncharacterized protein/similar to glioma pathogenesis-related protein 1; domains: allergen V5/Tpx-1-related, CAP, signal seq | A  B | | S,I  - | 2  - | 0.0008  - |  |
| 00000011278  (G1NED4) | IPI00580053  E1C3J7; [8] | Uncharacterized protein/similar to biotinidase; domains: carbon-hydrogen hydrolase/biotinidase, signal seq | A  B | | S,I  **S**,I | 5  10 | 0.0049  0.0108 | 0.0079 |
| 00000011286  (G1NED9) | IPI00571767  F1NY82 | Similar to Ras-GTPase-activating-like protein IQGAP1 | A  B | | -  S | -  3 | -  <0.0001 |  |
| 00000011352  (G1NEJ1)  00000011341  (G1NEI1) | IPI00586962  VEGFA | VEGF-A | A  B | | **S**,I  S | 5  3 | 0.0031  0.0034 | 0.0033 |
| 00000011396  (G1NEM6) | IPI00573007  Q91001; [8] | Thrombin | A  B | | S,I  **S**,I | 4  7 | 0.0047  0.0065 | 0.0056 |
| 00000011429  (G1NEQ0)  00000017635  (G3UPX5) | IPI00601590  Q98TD1; [8] | PIT 54 | A  B | | **S**,I  **S**,I | 12  16 | 0.0334  0.0978 | 0.0656 |
| 00000011466  (G1NET0) | IPI00582600  F1NH93 | Similar to ribosomal protein S20 | A  B | | **-**  S | -  2 | -  0.0003 |  |
| 00000011469  (G1NET3)  00000017809  (G5E7H3) | IPI00576698  F1NDK7, Q5ZI81 | Uncharacterized protein/dipeptidylpeptidase IV | A  B | | **S**,I  **S**,I | 11  9 | 0.0023  0.0022 | 0.0023 |
| 00000011485  (G1NEU7) | IPI00578127  E1BXD9; [8] | Similar to aminopeptidase A/glutamyl aminopeptidase | A  B | | S  - | 3  - | 0.0001  - |  |
| 00000011499  (G1NEV7) | IPI00603346  E1C648; [8] | Uncharacterized protein/similar to inositol monophosphatase 3 | A  B | | S,I  **S**,I | 2  4 | 0.0026  0.0046 | 0.0036 |
| 00000011507  (G1NEW3) | IPI00819050  F1N9R5 | Uncharacterized protein/similar to ceruloplasmin | A  B | | **S**,I  **S**,I | 7  9 | 0.0009  0.0018 | 0.0014 |
| 00000011529  (G1NEY3) | IPI00585941  F1N997; [8,16,17] | α-tubulin, shares most of its peptides with contaminant | A  B | | S,**I**  - | 2  - | 0.0059  - |  |
| 00000011532  (G1NEY6) | IPI00583507  Q5ZHM8; [8] | Similar to syntenin-1 | A  B | | **S**,I  **S**,I | 6  3 | 0.0128  0.0304 | 0.0216 |
| 00000011539  (G1NEZ2) | IPI00596234  F1NBU1 | Similar to ribosomal protein S16 | A  B | | **-**  S | -  2 | -  0.0002 |  |
| 00000011544  (G1NEZ6) | IPI00577678  F1NF64 | Uncharacterized protein/similar to complement factor I | A  B | | **S**,I  S | 6 (7)  7 | 0.0014  0.0015 | 0.0015 |
| 00000018802  (G3USW8)  00000011551  (G1NF02) | IPI00600922  E1BX73, LARGE2  [8] | Glycosyltransferase-like protein LARGE2 | A  B | | -  S,**I** | -  4 | -  0.0002 |  |
| 00000011569  (G1NF16) | IPI00582079  F1N8Z3 | Ras-related Rab-2a (fragment) | A  B | | S,**I**  **S**,I | 2  2 | 0.0003  0.0003 | 0.0003 |
| 00000011616  (G1NF52) | IPI00584633  F1P2Y9; [8] | Uncharacterized protein/similar to Kunitz-type protease inhibitor 1 | A  B | | S  **S**,I | 7  9 | 0.0062  0.0126 | 0.0094 |
| 00000011629  (G1NF62) | IPI00570671  K2CO; [8] | Keratin type II, cochleal; shares most peptides with contaminants and 00000011530 (G1NEY4) | A  B | | S,I  I | 2 (3)  1 (2) | 0.0006  0.0004 | 0.0005 |
| 00000019406  (G3UUE4)  00000011631  (G1NF64) | IPI00587509  Q4LDF5; [8] | DEC-205; domains: FN3, CLECT, ricin B | A  B | | **S**,I  **S**,I | 7  4 | 0.0003  0.0002 | 0.0003 |
| 00000011653  (G1NF82) | IPI00580829  F1NR48; [8] | Uncharacterized protein/similar to γ-glutamyl hydrolase | A  B | | S,**I**  S,I | 5  5 | 0.0246  0.0613 | 0.0430 |
| 00000011673  (G1NFA2) | IPI00585747  EF2 | Elongation factor 2 | A  B | | S,I  **S**,I | 4  14 | 0.0003  0.0016 | 0.0010 |
| 00000011728  (G1NFE9) | IPI00597840  F1NY83 | Uncharacterized protein; domain: sulfotransferase | A  B | | **S**,I  S,I | 2  5 | 0.0008  0.0070 | 0.0039 |
| 00000011766  (G1NFH8) | IPI00572652  H2AZ, etc | Histone H2A and possible variants | A  B | | **S**,I  **S**,I | 2 (3)  3 | 0.0311  0.0296 | 0.0304 |
| 00000011854  (G1NFQ1) | IPI00587762  F1NBF1; [8] | Uncharacterized protein/similar to β-mannosidase | A  B | | S,**I**  S,**I** | 6  14 | 0.0008  0.0020 | 0.0014 |
| 00000011860  (G1NFQ7) | IPI00601502  Q5ZLJ8 | Uncharacterized protein/similar to transmembrane protein 59 | A  B | | I  - | 3  - | 0.0002  - |  |
| 00000011891  (G1NFT2) | IPI00591099  F1NGM0 | Similar to EH domain-containing protein 4 | A  B | | S  - | 2  - | 0.0004  - |  |
| 00000011927  (G1NFW1) | IPI00571776  E1BRF7 | Similar to extracellular sulfatase Sulf-1; | A  B | | S  S | 2  2 | <0.0001  0.0002 | 0.0001 |
| 00000012007  (G1NG27) | IPI00591842  F1NWW5; [8] | Uncharacterized protein/similar to protocadherin Fat 1 | A  B | | S,I  **S**,I | 64  58 | 0.0041  0.0046 | 0.0044 |
| 00000012013  (G1NG31) | IPI00584072  F1NJU5 | Uncharacterized protein/similar to complement C8, α- polypeptide; domains: TSP_1, membrane attack complex component/perforin/complement C9, LDLRA_2, EGF | A  B | | S,I  **S**,I | 5  6 | 0.0017  0.0016 | 0.0017 |
| 00000012019  (G1NG36) | IPI00599064  Q5ZK58; [8] | Uncharacterized protein/similar to N-acylsphingosine amidohydrolase; domain: acid ceramidase-like | A  B | | S,I  **S**,I | 10  16 | 0.0289  0.0706 | 0.0498 |
| 00000012029  (G1NG45) | IPI00580953  F1NCI8 | Uncharacterized protein/similar to somatomedin-B and TSP_1-containing protein | A  B | | **S**,I  S | 3  4 | 0.0023  0.0026 | 0.0025 |
| 00000012095  (G1NG99) | IPI00603175  F1ND64 | Similar to serine protease HTRA1 | A  B | | -  **S**,I | -  4 | -  0.0072 |  |
| 00000012120  (G1NBG7) | IPI00599898  F1P1K3 | Actin-related protein 2/3 complex subunit 1 | A  B | | -  **S**,I | -  3 | -  0.0004 |  |
| 00000012138  (G1NGD3) | IPI00596487  F1P168 | Uncharacterized protein; domains: EGF_like, disintegrin, ADAM, ADAM_CR | A  B | | -  S,I | -  3 | -  0.0005 |  |
| 00000012248  (G1NGM4) | IPI00880383  F1P2U7 | Uncharacterized protein/similar to papillin; domains: TSP_1, Kunitz, IG, ADAM_TS, signal | A  B | | -  S | -  3 | -  0.0001 |  |
| 00000012272  (G1NPG2) | IPI00822777  F1NHT7, F1NJV8 | Similar to β-catenin | A  B | | S,I  S | 2  2 | 0.0012  0.0001 | 0.0007 |
| 00000012286  (G1NGQ1) | IPI00586903  F1P5L0 | Uncharactrized protein/similar to sodium-coupled monocarboxylate transporter 1 | A  B | | -  S,I | -  2 | -  0.0003 |  |
| 00000012328  (G1NGT5)  00000019182  (G3UTU7) | IPI00603267  ACTN2 | α-actinin-2; shares several peptides with α-actinin-1 (00000012468 (G1NH44)  00000018136 (G3UR37)) | A  B | | -  S | -  2 | -  <0.0001 |  |
| 00000012337  (G1NGU2) | IPI00590239  RSSA | 40S ribosomal protein SA | A  B | | -  S | -  4 | -  0.0007 |  |
| 00000012358  (G1NGW0) | IPI00599870  Q52LM4;[8] | β-galactosidase | A  B | | S,I  S,I | 12  13 | 0.0220  0.0523 | 0.0372 |
| 00000012365  (G1NGW5) | IPI00818947  F1NAD5, F1NKI1 | Cartilage-associated protein | A  B | | I  S,**I** | 6  7 | 0.0033  0.0022 | 0.0028 |
| 00000012380  (G1NGX6) | IPI00594205  F1NML1; [8] | Similar to VWFA and cache domain-containing protein 1 | A  B | | S  **S**,I | 9  7 | 0.0004  0.0005 | 0.0005 |
| 00000012385  (G1NGY0) | IPI00574953  E1C917 | Similar to ERO 1-like protein β (ER oxidoreductin-1-like) | A  B | | S,I  S,I | 7  5 | 0.0019  0.0032 | 0.0025 |
| 00000012453  (G1NH30) | IPI00575078  F1NXH2 | Uncharacterized protein/similar to UDP-N-acetyl-α-D-galactosamine:polypeptide N-acetylgalacactosaminyltransferase-like 1 | A  B | | I  S,**I** | 2  3 | 0.0003  0.0004 | 0.0004 |
| 00000012468  (G1NH44)  00000018136  (G3UR37) | IPI00597432  ACTN1; [8] | α-actinin-1 | A  B | | S,I  **S**,I | 3  3 (7) | 0.0001  0.0007 |  |
| 00000012478  (G1NH54) | IPI00573261  F1NG09; [8] | Uncharacterized protein/similar to programmed cell death 6-interacting protein isoform 1/dopamine receptor-interacting protein 4; domains:BRO1, ALIX V-shaped | A  B | | **S**,I  **S**,I | 22  21 | 0.0090  0.0125 | 0.0108 |
| 00000012493  (G1NH64) | IPI00576066  CALB1; [8] | Calbindin | A  B | | **S**,I  **S**,I | 6  9 | 0.0049  0.0113 | 0.0081 |
| 00000012544  (G1NHA1) | IPI00595914  E1BZF7 | Uncharacterized protein/similar to phospholipase B | A  B | | I  I | 2  2 | 0.0002  0.0005 | 0.0004 |
| 00000012553  (G1NHA8) | IPI00583961  F1NFU4 | Uncharacterized protein/similar to ependymin-related 1 (EPDR1) | A  B | | -  S | -  2 | -  0.0004 |  |
| 00000017426  (G3UPD1)  00000012647  (G1NHI9) | IPI00585886  NAGAB, F1NJF8 | α-N-acetylgalactosaminidase | A  B | | S,**I**  I | 4  6 | 0.0015  0.0019 | 0.0017 |
| 00000012667  (G1NHK4) | IPI00587747  F1P0J8 | Similar to thrombospondin-1 | A  B | | **S**,I  S,I | 24  30 | 0.0403  0.0328 | 0.0366 |
| 00000012712  (G1NHN9) | IPI00585118  F1NDH2; [8] | Angiotensin-2/angiotensinogen | A  B | | **S**,I  **S**,I | 6  10 | 0.0031  0.0047 | 0.0039 |
| 00000012733  (G1NHR0) | IPI00591868  E1BUR8 | Uncharacterized protein/similar to plasma glutamate carboxypeptidase | A  B | | S,**I**  S,I | 6  7 | 0.0064  0.0095 | 0.0080 |
| 00000012771  (G1NHU0) | IPI00578427  F1NG76; [8] | Uncharacterized protein/similar to matrilin-2 | A  B | | S,**I**  S,**I** | 13  17 | 0.0080  0.0157 | 0.0119 |
| 00000012807  (G1NHW9) | IPI00583929  Q6JLB2 | Proteasome subunit β-type | A  B | | S,**I**  S | 4  2 | 0.0009  0.0005 | 0.0007 |
| 00000012823  (G1NHY2) | IPI00595105  A1IMF0 | Ubiquitin carboxyl-terminal hydrolase isoenzyme L1 | A  B | | S,I  **S**,I | 4  4 | 0.0012  0.0015 | 0.0014 |
| 00000012834  (G1NHZ2) | IPI00598507  F1NHD7 | Uncharacterized protein/similar to chondroadherin-like protein; domains: leucine-rich repeats, signal seq | A  B | | -  **S**,I | -  6 | -  0.0012 |  |
| 00000012871  (G1NI18) | IPI00584318  E1C524 | Ras-related protein Ral-A | A  B | | -  S | -  2 | -  0.0019 |  |
| 00000012874  (G1NI21)  00000003564  (G1MXJ1) | IPI00578358  F1NH81  IPI00570679  E1BWJ7 | Similar to polyadenylate-binding protein 1 | A  B | | -  S | -  6 (8) | -  0.0002 |  |
| 00000012884  (G1NI29) | IPI00578632  1433Z; [8] | 14-3-3 protein ζ; shares peptide with other 14-3-3 proteins | A  B | | S,**I**  **S**,I | 6 (7)  6 (7) | 0.0082  0.0200 | 0.0141 |
| 00000012955  (G1NI79) | IPI00735163  E1C5F2, Q7LZG0 | Similar to PolyU-preferential ribonuclease CL1/ribonuclease T2; domain:ribonuclease T2-like | A  B | | S  S | 3  6 | 0.0016  0.0051 | 0.0034 |
| 00000013050  (G1NIF9) | IPI00574250  F1NWX6 | Uncharacterized protein/similar to plasminogen; shares 1 peptide with 00000000966 (G1MRV6) | A  B | | **S**,I  **S**,I | 6 (7)  7 (8) | 0.0012  0.0017 | 0.0015 |
| 00000013083  (G1NII7) | IPI00583347  F1NEL5; [8] | Similar to α-1,5-mannosylglycoprotein 6-β-N-acetylglucosaminyltransferase A | A  B | | **-**  S,I | -  2 | -  0.0006 |  |
| 00000017464  (G3UPG9)  00000013129  (G1NIM4) | IPI00578124  Q90681; [8] | Cation-independent mannose-6-phosphate receptor | A  B | | **S**,I  **S**,I | 15  20 | 0.0005  0.0007 | 0.0006 |
| 00000013135  (G1NIN0) | IPI00602021  F1NHI4 | Superoxide dismutase (extracellular) | A  B | | **S**,I  **S**,I | 7  8 | 0.0478  0.0710 | 0.0594 |
| 00000013161  (G1NIQ2) | IPI00576044  F1NYD7; [8] | Similar to exostosin-1 | A  B | | S,I  S,I | 3  5 | 0.0021  0.0051 | 0.0036 |
| 00000019442  (G3UUH8)  00000013168  (G1NIQ9) | IPI00603272  Q4F9K2; [8] | Tumor necrosis factor receptor superfamily member 11B/osteoprotegerin | A  B | | **S**,I  **S**,I | 10  9 | 0.0634  0.0646 | 0.064 |
| 00000013179  (G1NIS0) | IPI00858046  F1NYE0, NOV | NOV (nephroblastoma-overexpressed) /IGFBP-9; domains: TSP_1, VWC, cystine knot | A  B | | **S**,I  **S**,I | 6  8 | 0.0505  0.0433 | 0.0469 |
| 00000013204  (G1NIT9) | IPI00589837  E2RUH0 | Similar to ectonucleotide pyrophosphatase/phosphodiesterase 2 /extracellular lysophospholipase D | A  B | | **S**,I  **S**,I | 4  4 | 0.0005  0.0005 | 0.0005 |
| 00000013219  (G1NIV1) | IPI00883181  F1NHG5; [8] | Similar to Slit-2 | A  B | | S,**I**  S,I | 19  25 | 0.0103  0.0138 | 0.0121 |
| 00000013300  (G1NJ24) | IPI00822801  E1C9E2, F1CLE8 | Prominin-1 | A  B | | **S**,I  S | 2  3 | 0.0002  0.0001 | 0.0002 |
| 00000013304  (G1NJ28) | IPI00602028  F1NG86 | Uncharacterized protein; domain: FGF-binding 1 | A  B | | **S**,I  S | 6  3 | 0.0034  0.0019 | 0.0027 |
| 00000013318  (G1NJ40) | IPI00584379  E5G6H7; [8] | ADP-ribosyl cyclase CD157 (ADP-ribosyl cyclase 2) | A  B | | **S**,I  **S**,I | 5  4 | 0.0040  0.0063 | 0.0052 |
| 00000013320  (G1NJ42) | IPI00595609  F1NE01 | Uncharacterized protein/similar to C1q and tumor necrosis factor-like 6 | A  B | | S,I  **S**,I | 2  2 | 0.0008  0.0021 | 0.0015 |
| 00000013361  (G1NJ71)  00000018657  (G3USI2) | IPI00595705  Q5ZJI7; [8] | Uncharacterized protein/similar to epididymal secretory protein E1 (Niemann-Pieck disease type C protein) | A  B | | **S**,I  **S**,I | 9  10 | 0.4560  0.4022 | 0.4291 |
| 00000013377  (G1NJ85) | IPI00581678  WDR1  IPI00820144  F1NRI3 | WD-repeat-containing protein 1 | A  B | | **-**  S,I | -  3 | -  0.0002 |  |
| 00000013407  (G1NJB2) | IPI00572165  MYH9 | Myosin-9 | A  B | | **-**  S | -  20 (25) | -  0.0007 |  |
| 00000013465  (G1NJF8) | IPI00574658  TIMP3; [8] | TIMP-3 | A  B | | **S**,I  **S**,I | 7  7 | 0.0215  0.0331 | 0.0273 |
| 00000013480  (G1NJH0) | IPI00820421  F1NRQ9 | TGF-β3 | A  B | | **S**,I  **S**,I | 4  4 | 0.0018  0.0021 | 0.0020 |
| 00000013499  (G1NJI0) | IPI00572503  F1NQF6; [8] | Uncharacterized protein/similar to epididymis-specific α-mannosidase | A  B | | S,**I**  S,I | 23  26 | 0.0582  0.0701 | 0.0642 |
| 00000013595  (G1NJR0) | IPI00578484  Q9YGW6; [8] | Ezrin | A  B | | S,I  **S**,I | 13  12 | 0.0035  0.0056 | 0.0046 |
| 00000013606  (G1NJR8) | IPI00822589  F1NU36, F1NDD6 | Receptor-associated protein  (LDL receptor-/α2-macroglubulin receptor-associated) | A  B | | **-**  **S**,I | -  11 | -  0.0059 |  |
| 00000013617  (G1NJS7) | IPI00598803  E1BZN8; [8] | Similar to hepatocyte growth factor activator Q800Y7_MELGA | A  B | | **S**,I  **S**,I | 8  9 | 0.0068  0.0126 | 0.0097 |
| 00000013618  (G1NJS8) | IPI00591262  E1BS94 | Uncharacterized protein/similar to plasma α-L-fucosidase | A  B | | -  **S**,I | -  2 | -  0.0002 |  |
| 00000013629  (G1NJT6) | IPI00571879  NRX3A | Neurexin-3α | A  B | | S  **S**,I | 2  2 | 0.0001  0.0002 | 0.0002 |
| 00000013653  (G1NJV2) | IPI00599307  RLA1 | Acidic ribosomal protein P1 | A  B | | S,I  - | 2  - | 0.0010  - |  |
| 00000013656  (G1NJV5) | IPI00599871  F1NMI7 | Similar to carbohydrate sulfotransferase 11 | A  B | | S,I  **S**,I | 4  6 | 0.0047  0.0055 | 0.0051 |
| 00000013696  (G1NJY7) | IPI00585437  F1NXM7; [8,17] | Uncharacterized protein/similar to prostate stem cell antigen; domain: CD59 antigen/Ly-6 antigen, signal seq | A  B | | S,I  **S**,I | 5  5 | 0.1564  0.2084 | 0.1824 |
| 00000013707  (G1NJZ8) | IPI00590085  F1NJ89; [8] | Uncharacterized protein/similar to galactocerebrosidase | A  B | | S,I  S,**I** | 11  17 | 0.0333  0.0746 | 0.0540 |
| 00000013729  (G1NK19)  00000018554  (G3US83) | IPI00570770  ENPL, F1NWB7 | Endoplasmin/HSP90-β1; shares 1 peptide with 00000011247 (G1NEA9) | A  B | | S,**I**  S,I | 6  11 | 0.0023  0.0035 | 0.0029 |
| 00000013769  (G1NK51) | IPI00587419  F1NEW8; [8] | Uncharacterized protein; domains: α2-macroglobulin; 50% identity to ovostatin | A  B | | **S**,I  **S,**I | 41  45 | 0.0345  0.0728 | 0.0537 |
| 00000010818  (G1NDB0)  00000013771  (G1NK53) | IPI00581527  Q93410; [8] | Similar to calmodulin | A  B | | **S**,I  S | 4  4 | 0.0052  0.0081 | 0.0067 |
| 00000013826  (G1NK99) | IPI00571258  FGRL1 | FGF receptor-like 1 | A  B | | **-**  S | -  3 | -  0.0008 |  |
| 00000013859  (G1NKC5) | IPI00680520  IPI00573729  E1BUA6; [8] | Uncharacterized protein/similar to pantetheinase (VNN1); shares 3 peptides with 00000013862 (G1NKC7) | A  B | | **S**,I  **S**,I | 9 (12)  8 (11) | 0.2914  0.2819 | 0.2867 |
| 00000013862  (G1NKC7) | IPI00735160  Q5ZHM4 | Uncharacterized protein/similar to pantetheinase (VNN1); shares peptides with 00000013859 (G1NKC5); not the same! | A  B | | **S**,I  **S**,I | 5  7 | 0.0012  0.0039 | 0.0026 |
| 00000013863  (G1NKC8) | IPI00597023  E1C958;[8] | Uncharacterized protein/similar to legumain | A  B | | **S**,I  **S**,I | 12  11 | 0.0346  0.0705 | 0.0526 |
| 00000013869  (G1NKD4) | IPI00582046  F1P4F5; [8] | Uncharacterized protein KIAA1549; domain: DUF3827 | A  B | | S  S | 5  6 | 0.0009  0.0018 | 0.0014 |
| 00000013870  (G1NKD5) | IPI00583111  Q98TQ8; [8] | Connective tissue growth factor | A  B | | S  - | 3  - | 0.0003  - |  |
| 00000013928  (G1NKH5) | IPI00596449  E1BS56 | Uncharacterized protein/similar to α1-antitrypsin/serpin A4 | A  B | | **S**,I  **S**,I | 6  4 | 0.0010  0.0011 | 0.0011 |
| 00000013932  (G1NKH9) | IPI00591427  E1C7T1; [8] | Uncharacterized protein/similar to α1-antitrypsin/serpin A1 | A  B | | **S**,I  **S**,I | 11  9 | 0.0174  0.0109 | 0.0142 |
| 00000014033  (G1NKQ7) | IPI00588898  F1NZN0; [8] | Similar to protease-associated domain-containing protein 1 | A  B | | **-**  **S**,I | -  2 | -  0.0009 |  |
| 00000014088  (G1NKV0)  00000017218  (G3UT92)  00000018929  (G3UNT8) | IPI00596573  F1N9D0; [8] | Similar to mannosyl-oligosaccharide 1,2-α-mannosidase 1A | A  B | | S,**I**  S,I | 13  14 | 0.0573  0.0579 | 0.0576 |
| 00000014109  (G1NKW6)- | IPI00821927  F1NVQ7; F1NKL4 | Similar to cytoplasmic dynein 1 heavy chain 1 | A  B | | -  S | -  5 | -  <0.0001 |  |
| 00000014116  (G1NKX1) | IPI00596586  HS90A; [8] | HSP90-α; shares 4 peptides with 00000011247 (G1NEA9; HSP90-β) | A  B | | S,I  S,I | 6 (9)  7 | 0.0011  0.0034 | 0.0023 |
| 00000014122  (G1NKX7) | IPI00598999  E1C4N9; [8] | Tyrosine-protein kinase receptor | A  B | | **S**,I  **S**,I | 21  24 | 0.0028  0.0024 | 0.0026 |
| 00000014163  (G1NL13) | - | Similar to N-acetyl-β-glucosaminyl glycoprotein 4-β-N-acetyl-galactosaminyl transferase 1 | A  B | | I  I | 2  3 | <0.0001  0.0003 | 0.0002 |
| 00000014167  (G1NL16) | IPI00573166  KCRB; [8] | Creatine kinase B-type | A  B | | S,I  **S**,I | 10  10 | 0.0110  0.0187 | 0.0248 |
| 00000014265  (G1NL92) | IPI00602073  Q90W23 | PDGF B-chain | A  B | | S  S | 2  2 | 0.0028  0.0013 | 0.0021 |
| 00000014421  (G1NLL4)  00000017361  (G1NLL6)  00000014423  (G3UP71) | IPI00596515  F1NEM7,  Q8QGD9, Q90713) | Galectin-3 | A  B | | S  S | 3  6 | 0.0018  0.0027 | 0.0023 |
| 00000014484  (G1NLS0) | IPI001033  LDHB; [8] | L-lactate dehydrogenase B | A  B | | **S**,I  **S**,I | 7  8 | 0.0083  0.0094 | 0.0089 |
| 00000014567  (G1NLZ2)  00000006367  (G1N3P8) | IPI00578788  F1NZ94; [8,16] | Uncharacterized protein/similar to Scube 1; domains: EGF, CUB, thrombomodulin, growth factor receptor | A  B | | S,**I**  S,I | 12 (16)  11 (14) | 0.0883  0.0699 | 0.0791 |
| 00000014574  (G1NLZ7) | IPI00599470  F1NDL4 | Uncharacterized protein/similar to nidogen-2 | A  B | | **S**,I  **S**,I | 10  8 | 0.0016  0.0017 | 0.0017 |
| 00000014584  (G1NM06) | IPI00820938  Q5ZKX2  IPI00570965  F1NCS8 | Similar to Psmc6; domain:AAA+ ATPase | A  B | | **-**  S | -  2 | -  <0.0001 |  |
| 00000014615  (G1NM32)  00000014616  (G1NM33) | IPI00588009  F1NX21, F1NX20 | Uncharacterized protein/similar to CD109 antigen | A  B | | **S**,I  **S**,I | 16  17 | 0.0015  0.0022 | 0.0019 |
| 00000014622  (G1NM38) | IPI00581988  F1N9H4; [8] | Elongation factor 1α | A  B | | S,**I**  **S**,I | 7  6 | 0.0117  0.0192 | 0.0155 |
| 00000014627  (G1NM42)  00000018153  (G3UR54) | IPI00570704  FBLN1; [8] | Fibulin-1 | A  B | | -  S,I | -  2 | -  0.0003 |  |
| 00000018640  (G3USG8;  A4HU04)  00000014700  (G1NMA0) | IPI00586681  Q9IBC9; [8] | CD9 fragment, domains: tetraspanin | A  B | | S,I  - | 2  - | 0.0148  - |  |
| 00000017211  (G3UNT2)  00000014743  (G1NMD8) | IPI00596765  Q9W6J2; [8] | Glutathione S-transferase | A  B | | S,I  S,I | 2 (3)  2 | 0.0007  0.0002 | 0.0005 |
| 00000014813  (G1NMJ9) | IPI00683014  Q5QHR9 | Tumor necrosis factor-α- converting enzyme | A  B | | S,**I**  S,**I** | 4  2 | 0.0002  0.0002 | 0.0002 |
| 00000019513  (G3UUP5)  00000014815  (G1NMK1) | IPI00577739  1433T; [8] | 14-3-3 protein θ; shares 1 peptide with other 14-3-3 proteins | A  B | | S,I  **S**,I | 6  6 | 0.0025  0.0027 | 0.0026 |
| 00000014824  (G1NML0) | IPI00586516  F1NK96 | Similar to protein disulfide isomerase family A member 6 | A  B | | S,**I**  **S**,I | 3  5 | 0.0011  0.0009 | 0.0010 |
| 00000014850  (G1NMM9) | IPI00603403  MATN3 | Matrilin-3 | A  B | | -  S,I | -  3 | -  0.0003 |  |
| 00000014853  (G1NMN2) | IPI00580076  F1NV24 | Similar to syndecan-1, fragment (N-term) | A  B | | S  S | 2  2 | 0.0079  0.0102 | 0.0091 |
| 00000014896  (G1NMR6) | IPI00594653  G3P; [8] | GAPDH | A  B | | S,I  **S**,I | 8  7 | 0.0064  0.0030 | 0.0047 |
| 00000014904  (G1NMS3)  00000018200  (G3UR98) | IPI00593138  RAB10; [8] | Ras-related Rab-10 | A  B | | S,**I**  **S**,I | 3  2 | 0.0020  0.0015 | 0.0018 |
| 00000014942  (G1NMV6) | IPI00604279  Q9YGP0; [67,8,9,16,17] | Clusterin | A  B | | **S**,I  **S**,I | 14  10 | 0.2271  0.3971 | 0.3121 |
| 00000014944  (G1NMV8;  Q70I42) | IPI00582452  TPIS; [8] | Triosephosphate isomerase | A  B | | -  S | -  4 | -  0.0010 |  |
| 00000014961  (G1NMX0)  00000018619  (G3USE6) | IPI00571345  PHB2 | Prohibitin-2 | A  B | | I  - | 3  - | 0.0003  - |  |
| 0000014972  (G1NMY0) | IPI00584356  F1NAC3 | Uncharacterized protein/similar to C1r protein; domains: Sushi/SCR/CCP, CUB, peptidase S1/S6, EGF | A  B | | S,I  **S**,I | 12  13 | 0.0072  0.0059 | 0.0066 |
| 0000014980  (G1NMY8) | IPI00593795  E1C3B9; [8] | Similar to calsyntenin-3 | A  B | | **S**,I  **S**,I | 12  11 | 0.0092  0.0087 | 0.0090 |
| 0000014991  (G1NMZ9) | IPI0000571996  F1N9G6; [8] | Uncharacterized protein/similar to glutathione S-transferase κ1 | A  B | | S,**I**  S,**I** | 6  3 | 0.0020  0.0010 | 0.0015 |
| 0000015037  (G1NN37) | IPI00577962  CATB; [8] | Cathepsin B | A  B | | **S**,I  **S**,I | 16  19 | 0.0626  0.1265 | 0.0946 |
| 0000015039  (G1NN39) | IPI00574804  GLL11; [8] | Gallinacin-11/β-defensin-11/VMO-II | A  B | | **S**,I  **S**,I | 5  4 | 0.0068  0.0123 | 0.0096 |
| 0000015070  (G1NN67) | IPI00577152  F1NFI1 | Uncharacterized protein/similar to Cys-rich secretory protein 3 | A  B | | S  S | 4  8 | 0.0012  0.0114 | 0.0063 |
| 0000015109  (G1NN99) | IPI00574870  F1NEZ6 | Uncharacterized protein/similar to G protein-coupled receptor116 | A  B | | S  **S**,I | 5  6 | 0.0005  0.0007 | 0.0006 |
| 0000015126  (G1NNB3)  0000015127  (G1NNB4)  0000018374  (G3URR1) | IPI00819216  AT1A1 | Na^+^/K^+^-transporting ATPase subunit α1 | A  B | | -  S,I | -  3 | -  0.0002 |  |
| 0000015135  (G1NNC1) | IPI00579759  E1C9B0 | Uncharacterized protein7similar to b-1,4-galactosyltransferase 4 | A  B | | S,I  S | 2  2 | 0.0003  0.0004 | 0.0004 |
| 0000015155  (G1NND5) | IPI00580509  APOV1; [8] | Apovitellenin-I | A  B | | S,**I**  **S**,I | 2  2 | 0.0206  0.0030 | 0.0118 |
| 0000015160  (G1NND9) | IPI00590522  E1C202 | Similar to phospholipase A2; domains: phospholipase A2, signal seq | A  B | | **S**,I  **S**,I | 6  6 | 0.0404  0.0330 | 0.0367 |
| 0000015189  (G1NNG6)  0000018270  (G3URG3) | IPI00576374  Q702H9, Q70D52  [8] | α-2,3-sialyltransferase | A  B | | **S**,I  S | 8  8 | 0.0057  0.0068 | 0.0063 |
| 0000015203  (G1NNH8) | IPI00811795  E1C0S4 (partial) | Uncharacterized protein/similar to Target of NESH-SH3; domains: FN3 | A  B | | **-**  S | -  2 | -  <0.0001 |  |
| 0000015252  (G1NNL9) | IPI00584625  E1C6L4; [8] | Uncharacterized protein/similar to Vit K-dependent protein S | A  B | | **S**,I  **S**,I | 15  14 | 0.0206  0.0366 | 0.0286 |
| 0000015262  (G1NNM7) | IPI00579585  H9KZM9 | Uncharacterized protein/similar to Tcn2; domains: cobalamin-binding transporter | A  B | | **S**,I  **S**,I | 12  8 | 0.0432  0.0363 | 0.0398 |
| 0000015265  (G1NNM9) | IPI00600389  F1NP56 | Uncharacterized protein/similar to galactosylceramide sulfotransferase; shares 1 peptide with 00000003022 (G1MWB3) | A  B | | I  - | 3  - | 0.0007  - |  |
| 0000015275  (G1NNN9) | IPI00595079  CREG1; aa76-192; [8] | CREG1, fragment | A  B | | **S**,I  **S**,I | 3  2 | 0.0070  0.0168 | 0.0119 |
| 0000015289  (G1NNQ0) | IPI00578852  Q5ZMJ1; [8] | Uncharacterized protein/similar to [protein ADP-ribosylarginine] hydrolase | A  B | | S,**I**  S,I | 8  8 | 0.0100  0.0203 | 0.0152 |
| 0000015315  (G1NNR9) | IPI00820995  F1NQH6 | Similar to ROBO2 fragment ROBO2_MOUSE; (aa125-1469) | A  B | | S  S | 3  3 | 0.0001  <0.0001 | <0.0001 |
| 0000015319  (G1NNS3) | IPI00592330  F1ND59; [8,9] | Uncharacterized protein/similar to HSP70-13 | A  B | | S,I  S,I | 13  20 | 0.0235  0.0464 | 0.0350 |
| 0000015324  (G1NNS6) | IPI00586017  F1NSL7 | Similar to Coxsackie-adenovirus-receptor-homolog | A  B | | -  **S**,I | -  2 | -  0.0003 |  |
| 0000015345  (G1NNT9)  0000018431  (G3URW3)  0000018669  (G3USJ4) | IPI00581720  Q9DGJ7, Q9DGJ8  [8,9] | β-amyloid protein 751;  similar to amyloid β A4  β-amyloid protein 695; | A  B | | **S**,I  **S**,I | 15 (16)  20 | 0.0479  0.0651 | 0.0565 |
| 0000015347  (G1NNU1) | IPI00594926  F1P3T6 | Similar to ADAMTS1 | A  B | | S,I  S,**I** | 4  5 | 0.0008  0.0012 | 0.0010 |
| 0000015356  (G1NNU8) | IPI00595594  TCPQ | T-complex protein 1 subunit θ | A  B | | -  **S**,I | -  5 | -  0.0001 |  |
| 0000015427  (G1NP04) | IPI00573994  F1NY88; [8] | Uncharacterized protein/similar to transmembrane protease serine 2 | A  B | | S,I  S | 5  2 | 0.0007  0.0002 | 0.0005 |
| 0000015434  (G1NP09)  0000019792  (G5E7Y1) | IPI00584649  F1NY91; [8] | Uncharacterized protein/similar to FAM3B | A  B | | **-**  S | -  2 | -  0.0005 |  |
| 0000015436  (G1NP11) | IPI00583503  E1BZ37 | Uncharacterized protein/trefoil family peptide 2 (TFF-2); domains: P-type trefoil; signal seq | A  B | | **S**,I  **S**,I | 7  9 | 0.8479  1.4201 | 1.1340 |
| 0000015478  (G1NP43) | IPI00822944  F1NIX1 | Uncharacterized protein/similar to ATP-dependent RNA helicase | A  B | | -  S | -  3 (4) | -  00001 |  |
| 0000015482  (G1NP47)  0000018490  (G3US21) | IPI00593541  Q1XIH7; [8] | Renin/prorenin receptor | A  B | | S,I  S,I | 11  9 | 0.0636  0.0369 | 0.0503 |
| 0000015519  (G1NP78) | IPI00601711  F1NPF0, IF2G | Eukaryotic translation initiation factor 2 subunit 3 | A  B | | -  S | -  4 | -  0.0001 |  |
| 0000015525  (G1NP83) | IPI00601900  F1NNS8; [8] | Similar to peroxiredoxin-4; shares 1 peptide with 00000010828 (G1NDB7) | A  B | | S,**I**  **S**,I | 6 (7)  6 | 0.0133  0.0098 | 0.0116 |
| 0000015543  (G1NP98) | IPI00571343  F1NUC4 | Uncharacterized protein/similar to G protein-coupled receptor 64 | A  B | | **-**  S | -  2 | -  0.0006 |  |
| 0000015587  (G1NPE0) | IPI00684448  E1C897 | Uncharacterized protein/similar to aspartic protease | A  B | | **S**,I  S,I | 6  3 | 0.0017  0.0008 | 0.0013 |
| 0000015598  (G1NPF0)  0000017907  (G5E7N3) | IPI00592782  KALM | Anosmin-1 | A  B | | S  **S**,I | 10  10 | 0.0024  0.0033 | 0.0029 |
| 00000015622  (G1NPG7) | IPI00818123  IPI00600989  F1NFF6; F1NBW8;Q8AYM3 | ADP/ATP translocase 1/ATP-ADP antiporter | A  B | | -  S | -  2 | -  0.0002 |  |
| 0000015665  (G1NPK1) | IPI00601503  MGT4A | α-1,3-mannosyl glycoprotein 4-β-N-acetylglucosaminyltransferase A | A  B | | I  - | 2  - | 0.0002  - |  |
| 0000015671  (G1NPK6) | IPI00573323  LYG, [8] | Lysozyme G | A  B | | **S**,I  **S**,I | 9  9 | 0.3024  0.1267 | 0.2146 |
| 0000015680  (G1NPL3) | CHSTA | Carbohydrate sulfotransferase 10 | A  B | | **-**  **S**,I | -  2 | -  0.0002 |  |
| 0000015714  (G1NPP3) | IPI00574801  E1BV28; [8] | UDP-glucuronic acid decarboxylase I; mitochondrial; domain: NAD-dependent epimerase | A  B | | S,I  S,I | 13  12 | 0.0569  0.0754 | 0.0662 |
| 0000015731  (G1NPQ8) | IPI00578932  F1P3F0; [8] | Similar to growth arrest protein 6/MGC68463 | A  B | | **S**,I  **S**,I | 9  9 | 0.0038  0.0056 | 0.0047 |
| 0000015755  (G1NPT0)  0000015754  (G1NPS9) | IPI00582859  F1P2Q3 | Similar to collagen a1 (IV) **^1^** | A  B | | -  S | -  2 | -  <0.0001 |  |
| 0000015798  (G1NPW6) | IPI00594476  F1P053, DNJC3; [8] | Similar to DnaJ subfamily C member 3 | A  B | | S,**I**  S,I | 15  14 | 0.0141  0.0180 | 0.0161 |
| 0000015832  (G1NPZ0) | IPI00581054  E1BQD1 | Uncharacterized protein; domain: DJ-1_PfpI | A  B | | **S**,I  S,I | 2  2 | 0.0003  0.0003 | 0.0003 |
| 0000015833  (G1NPZ1) | IPI00576538  F1NZF1; [8] | Uncharacterized protein/similar to ceroid-lipofuscinosis neuronal protein 5 | A  B | | **S**,I  **S**,I | 7  9 | 0.0127  0.0248 | 0.0188 |
| 0000015841  (G1U9Q8; P84479) | IPI00590350  HBB, Q90864  [8,16,17] | Hemoglobin β-chain  Hemoglobin β-A | A  B | | I  - | 6  - | 0.0041  - |  |
| 0000015865  (G1NQ14) | IPI00595514  Q25C35; [8] | Tiarin-like protein | A  B | | **S**,I  **S**,I | 4  4 | 0.0009  0.0014 | 0.0012 |
| 0000015910  (G1NQ47) | IPI00586374  E1BXC2 | Similar to S-formylglutathione hydrolase | A  B | | S,I  - | 2  - | 0.0003  - |  |
| 0000015953  (G1NQ84) | IPI00573359  F1P587; [8] | Similar to complement C4-1 | A  B | | S,I  S,I | 14  14 | 0.0011  0.0019 | 0.0015 |
| 0000015961  (G1NQ91) | IPI00595980  Q6DMS3 | Periostin | A  B | | S,I  S,I | 75 (77)  69 (71) | 10.2270  11.5484 | 10.8877 |
| 0000017921  (G1NQJ5)  0000017979  (G1NQJ4)  0000016090  0000016091 | IPI00593242  E1C9E0  IPI00820386  F1N995 | Uncharacterized protein/similar to stromelysin-1/2a | A  B | | -  S | -  3 | -  0.0003 |  |
| 0000016152  (G1NQP8) | IPI00577371  F1NWG2; [8] | Uncharacterized protein/similar to dipeptidyl peptidase 1/cathepsin C | A  B | | S,**I**  S,**I** | 11  14 | 0.0076  0.0194 | 0.0137 |
| 0000016178  (G1NQR9) | IPI00577009  F1NVT4, F1NVT5; [8] | Similar to Odz4/terneurin-4 | A  B | | **S**,I  S,I | 2  3 | 0.0001  0.0002 | 0.0002 |
| 0000016199  (G1NQT4) | IPI00570989  E1C292; [8] | Similar to calpain-5 | A  B | | -  S | -  3 | -  0.0001 |  |
| 0000016213  (H9H1Z2) | IPI00601791  F1NB83, partial; [8] | Uncharacterized protein/similar to tripeptidyl peptidase 1 | A  B | | S,I  S,I | 3  3 | 0.0103  0.0193 | 0.0148 |
| 0000016222  (G1NQU6) | IPI00595238  Q9PW81; [8] | Erythroid-specific folate receptor | A  B | | **S,**I  **S**,I | 5  4 | 0.0146  0.0142 | 0.0145 |
| 0000016230  (G1NQV4) | IPI00822610  F1NPA9 | Ribosomal protein S3 | A  B | | -  S | -  2 | -  0.0009 |  |
| 0000016259  (G1NQX3) | IPI00584082  F1NDB4 | Uncharacterized protein/similar to leucine-rich repeat-containing protein 32 | A  B | | S,I  **S**,I | 3  5 | 0.0027  0.0023 | 0.0025 |
| 0000016343  (G1NR21) | IPI00582200  F1NEZ4 | Similar to SLIT and NTRK-like family member 2 | A  B | | S,I  - | 3  - | 0.0002  - |  |
| 0000016390  (G1NR45) | IPI00579312 | Uncharacterized protein; domains: Kazal-type protease inhibitor; shares 1 peptide with 00000004638 (G1MZX1; ovoinhibitor) | A  B | | **S**,I  **S**,I | 2  2 | 0.0078  0.0046 | 0.0062 |
| 0000016417  (G1NR52)  0000016399  (G1NR62) | IPI00581002  F2Z4K9, UBB  [8] | Ubiquitin/polyubiquitin | A  B | | **S**,I  **S**,I | 6  4 | 0.1470  0.1348 | 0.1209 |
| 0000016404  (G1NR54) | - | Uncharacterized protein/similar to UDP-GlcNac: β-Gal β-1,3-N-acetylglucosaminyl transferase 4 | A  B | | S,**I**  S,**I** | 3  9 | 0.0031  0.0098 | 0.0065 |
| 0000016479  (G1NR95) | IPI00578425  E1BR90; [8] | Uncharacterize protein/similar to carbohydrate sulfotransferase 12 | A  B | | **S**,I  **S**,I | 12  10 | 0.0139  0.0194 | 0.0167 |
| 0000016573  (G1NRE7) | IPI00581140  E1C744 | Similar to serine protease 23 | A  B | | S,**I**  S,I | 8  6 | 0.0196  0.0043 | 0.0120 |
| 0000016579  (G1NRF1) | IPI00601632  E1C5F3; [8] | Uncharacterized protein/similar to CHST5; domain: carbohydrate (chondroitin) sulfotransferase | A  B | | S,I  S,I | 6  5 | 0.0021  0.0026 | 0.0024 |
| 0000016580  (G1NRF2) | IPI00583184  TSK, [8,16] | Tsukushin | A  B | | S,**I**  **S**,I | 12  11 | 0.2178  0.2859 | 0.2519 |
| 0000016597  (G1NRF9) | IPI00822809  DGGR58 | Meleagrin/gallin | A  B | | **S**,I  **S**,I | 4  4 | 0.3654  0.4673 | 0.4164 |
| 0000016652  (G1NRJ1)  0000016647  (G1NRH10) | IPI00604087  H2B1  IPI00822431  F1NCM0; [8] | Histone H2B | A  B | | S,**I**  S | 2  4 | 0.0014  0.0059 | 0.0037 |
| 0000016636  (G1NRH7) | IPI00682229  F1NFT0 | Similar to polypeptide N-acetyl-galactosaminyltransferase 4(GALNT); shares peptide with 00000007369 (G1N5V3) | A  B | | S,**I**  S,I | 9  10 (11) | 0.0094  0.0126 | 0.0110 |
| 0000016644  (G1NRJ0)  0000016645  0000016651 | IPI00572919  etc; [8] | Histone H4 | A  B | | **S**,I  **S**,I | 5  6 | 0.0189  0.0668 | 0.0429 |
| 0000016715  (G1NRM0) | IPI00592947  F1NLT2; [8] | Uncharacterized protein/similar to torsin 4-A-A | A  B | | I  - | 4  - | 0.0006  - |  |
| 0000016856  (G1NRV7) | IPI00602775  Q5ZK57; [8] | Uncharacterized protein/similar to UDP-GlcNAc: β-Gal β-1,3-N-acetylglucosaminyl-transferase 2 | A  B | | S  S | 2  2 | 0.0008  0.0011 | 0.0010 |
| 0000016980  (G1NS19) | IPI00595022  IPI00871073  NTF3 | Neurotrophin-3 | A  B | | S,I  - | 2  - | 0.0003  - |  |
| 0000016987  (G1NS21) | IPI00590465  F1NRQ5 | Uncharacterized protein/similar to lactosylceramide 1,3-N-acetyl-β-D-glucosaminyltransferase | A  B | | I  - | 2  - | 0.0005  - |  |
| 0000017228  (H9H250) | IPI00574718  F1NXL1; [8] | Uncharacterized protein/similar to reticulon-4 receptor –like 2 | A  B | | S,I  S,I | 6  5 | 0.0073  0.0104 | 0.0089 |
| 0000017229  (G3UNU8) | F1P590 | Similar to NHL-repeat-containing protein 3, fragment | A  B | | S,I  S,I | 2  2 | 0.0039  0.0098 | 0.0069 |
| 0000017341  (G3UP52)  (G1NA96) | IPI00594441  DNAS1, Q9PZY8  IPI00679732  H9L1H2, H9KZ16 | Deoxyribonuclease; signal sequence | A  B | | -  S | -  2 (3) | -  0.0004 |  |
| 0000017652  (G3UPZ0)  0000005689  (G1N265) | IPI00574951  SEM3C; [8] | Semaphorin-3C | A  B | | S,**I**  S,**I** | 6  6 | 0.0019  0.0021 | 0.0020 |
| 0000017677  (G3UQ12)  0000006376  (G1N3Q5)  0000019222 | IPI00602351  Q6JG52, Q98942  [8] | Pancreatic α-amylase | A  B | | -  **S**,I | -  6 | -  0.0018 |  |
| 0000017706  (G3UQ40) | IPI00572019  F1NKR1; [8] | Uncharacterized protein/similar to Bapxr protein; domain: glycoside hydrolase family 38 | A  B | | -  S,I | -  2 | -  0.0002 |  |
| 0000017748  (G3UQ78) | IPI00576481  E1C4I5; [8] | Uncharacterized protein/similar to heparin sulfate glucosaminyl 3-O-sulfotransferase 1 | A  B | | S,**I**  S,**I** | 4  4 | 0.0395  0.0636 | 0.0516 |
| 0000017809  (G5E7H3)  0000011469  (G1NET3) | IPI00576698  Q5ZI81 | Uncharacterized protein/similar to venom dipeptidyl peptidase IV A6MJH2_OXYSU | A  B | | **S**,I  **S**,I | 11  9 | 0.0023  0.0022 | 0.0023 |
| 0000017903  (G5E7M9) | IPI00685019  F1NSD0 | Similar to immunoglobulin light chain LV1_CHICK; shares peptides with other Ig light chains | A  B | | **-**  **S**,I | -  1 (3) | -  0.0466 |  |
| 0000017999  (G3UQQ8) | IPI00597755  F1NSD3; [8] | Immunoglobulin light chain; shares peptides with other Ig light chains | A  B | | **S**,I  S | 3  2 | 0.0198  0.0057 | 0.0128 |
| 0000018054  (G3UQV9) | IPI00584436  F1N8E5 | Uncharacterized protein/similar to serine protease 23 | A  B | | S,**I**  S,**I** | 5  4 | 0.0020  0.0006 | 0.0013 |
| 0000018172  (G3UR71) | IPI00685019  F1NSD0 | Immunoglobulin light chain; shares peptides with many other Ig light chains | A  B | | **S**,I  - | 1 (13)  - | 0.5472  - |  |
| 0000018231  (G3URC8)  0000009084  (G1N9J3) | IPI00571731  RET4; [8] | Retinol-binding protein 4 | A  B | | **S**,I  S | 4  3 | 0.0046  0.0023 | 0.0035 |
| 0000018349  (G3URN7) | IPI00591339  F1NZW5, Q5ZKY8 | Uncharacterized protein; domain: seven_cysteines/MANSC | A  B | | S  S | 2  2 | 0.0009  0.0006 | 0.0008 |
| 0000018441  (G3URX3)  0000004868  (G1NQD9) | IPI00594564  E1BWU2; [8] | Uncharacterized protein/similar to nexin-1 | A  B | | **S**,I  **S**,I | 21  23 | 0.1495  0.2050 | 0.1773 |
| 0000018475  (G3US06) | IPI00575870  F1NSR8 | Uncharacterized protein/similar to γ-interferon-inducible lysosomal thiol reductase | A  B | | **S**,I  **S**,I | 2  3 | 0.0019  0.0047 | 0.0033 |
| 0000018544  (G3US73)  0000003883  (G1MY76) | IPI00591409  SIAT1; [8] | β-galactoside α-2,6-sialyltransferase 1 | A  B | | **S**,I  **S**,I | 8  8 | 0.0177  0.0199 | 0.0112 |
| 0000018661  (G3USI6) | IPI00597109  AMPN; [8] | Aminopeptidase N; aminopeptidase Ey | A  B | | **S,**I  S,I | 4  3 | 0.0389  0.0076 | 0.0233 |
| 0000019601  (G3UUY0)  0000018676  (G3USK1) | IPI00683984  F1NSC8 | Similar to immunoglobulin light chain LV1_CHICK; shares peptides with other Ig light chains | A  B | | **S**,I  **S**,I | 3  2 | 0.2057  0.1179 | 0.1618 |
| 0000018816  (G3USY2) | IPI00590762  Q9DEQ8 | Teneurin-4 | A  B | | **S**,I  **S**,I | 5  6 | 0.0058  0.0011 | 0.0035 |
| 0000018822  (G3USY8) | IPI00685019  F1NSD0 | Similar to immunoglobulin light chain LV1_CHICK; shares peptides with other Ig light chains | A  B | | **S**,I  **S**,I | 3  3 (4) | 0.1638  0.4854 | 0.3246 |
| 0000018878  (G3UT43) | IPI00588727  F1NCN3; [8,16] | EGF-like repeat and discoidin domain I-like protein 3/EDIL3, fragment | A  B | | **S**,I  **S**,I | 14  15 | 3.9859  3.4145 | 3.7000 |
| 0000018940  (G3UTA1) | IPI00588322  FIBB; [8] | Fibrinogen β-chain | A  B | | S,I  S,I | 3  4 | 0.0005  0.0006 | 0.0006 |
| 0000018959  (G3UTB7)  0000010189  (G1NBY3) | IPI00603030  A3FB57 | Receptor-type tyrosine-protein phosphatase LAR | A  B | | **S**,I  **S**,I | 21 (22)  21 (22) | 0.0043  0.0048 | 0.0046 |
| 0000019196  (G3UTW1)  0000019026  (G1MWU7) | IPI00578559  F1NE61; [8,9,17] | Uncharacterized protein/polymeric Ig receptor; fragment | A  B | | **S**,I  **S**,I | 21  18 | 1.5693  1.6033 | 1.5863 |
| 0000019038  (G3UTJ0) | IPI00595270  TIMP2; [8] | TIMP2 | A  B | | **S**,I  **S**,I | 5  8 | 0.0659  0.0582 | 0.0621 |
| 000000190072  (G5E7N5)  00000018037  (G3US48) | IPI00818032  F1NB81  IPI00579861  E1C566 | Uncharacterized protein/similar to protocadherin-16 | A  B | | S  S | 5  5 | 0.0004  0.0005 | 0.0005 |
| 00000019384  (H9H2J4) | IPI00577729  H9KZT2 | VH1 protein; shares several peptides with other VH1 proteins | A  B | | -  S,I | -  2 | -  0.0439 |  |
| 0000019433  (G3UUH0)  0000009709  (G1NAW6) | IPI00588868  IF4A2 | Eukaryotic initiation factor 4A-II | A  B | | S,I  S | 6  5 | 0.0005  0.0007 | 0.0006 |
| 0000019564  (G3UUU5) | IPI00580719  E1BSH4 | Uncharacterized protein/similar to core 1 β-3-galactosyltransferase-specific molecular chaperone | A  B | | S,**I**  **S**,I | 9  6 | 0.0047  0.0030 | 0.0039 |
| 0000019607  (G3UUY6) | IPI00571767  F1NY82 | IQGAP1 | A  B | | -  S | -  3 | -  0.0001 |  |
| 0000019686  (G3UV23) | IPI00571119  E1B2H8; [8] | Similar to extracellular sulfatase Sulf-2 | A  B | | S,**I**  - | 2  - | 0.0165  - |  |
| 0000019699  (G3UV35) | IPI00971408  Q98921 | HEMCAM | A  B | | S  S | 4  6 | 0.0016  0.0054 | 0.0035 |
| 0000019749  (G3UV75) | IPI00591966  RAB8A | Ras-related Rab-8A | A  B | | I  - | 2  - | 0.0002  - |  |
| 00000019777  (G5E7W7)  00000017368  (G1MYI4)  00000013820 | IPI00598461  TPM1 | Similar to tropomyosin α3 | A  B | | -  S | -  4 | -  0.0015 |  |
| **No entry!** | OC17, Q9PRS8  [47,8,9,16] | Ovocleidin-17 | A  B | | **S**,I  S | 2  2 | >0.0183  >0.0150 | >0.0167 |
|  |  |  |  | |  |  |  |  |

A, strain Converter; B, strain Big6. S, acid-soluble fraction; I, acid-insoluble fraction; major fraction (according to number of identified peptides) indicated in bold. Previous identification in chicken eggshell matrix is indicated by reference (see main text). For proteins detected in several studies the ordering of references is according to their publication date, starting with the earliest. To facilitate cross-reference to previously identified chicken eggshell proteins we have added IPI accession numbers and corresponding chicken UniProt accession numbers. In the case of overlapping sequences with shared peptides razor and unique peptides as assigned by MaxQuant (peptide numbers in brackets) were used to determine iBAQ intensities. **^1^** Collagens were predominantly identified with non-triple helical peptides and a few peptides from the junction between non-triple helical and triple helical domains because proline hydroxylation was not included among the variable modifications. However, calculation of emPAI using only non-triple helical domains confirmed the low to very low abundance of collagens in the eggshell matrix.
